# Unraveling the functional dark matter through global metagenomics

**DOI:** 10.1038/s41586-023-06583-7

**Published:** 2023-10-11

**Authors:** Georgios A. Pavlopoulos, Fotis A. Baltoumas, Sirui Liu, Oguz Selvitopi, Antonio Pedro Camargo, Stephen Nayfach, Ariful Azad, Simon Roux, Lee Call, Natalia N. Ivanova, I. Min Chen, David Paez-Espino, Evangelos Karatzas, Novel Metagenome Protein Families Consortium, Silvia G. Acinas, Nathan Ahlgren, Graeme Attwood, Petr Baldrian, Timothy Berry, Jennifer M. Bhatnagar, Devaki Bhaya, Kay D. Bidle, Jeffrey L. Blanchard, Eric S. Boyd, Jennifer L. Bowen, Jeff Bowman, Susan H. Brawley, Eoin L. Brodie, Andreas Brune, Donald A. Bryant, Alison Buchan, Hinsby Cadillo-Quiroz, Barbara J. Campbell, Ricardo Cavicchioli, Peter F. Chuckran, Maureen Coleman, Sean Crowe, Daniel R. Colman, Cameron R. Currie, Jeff Dangl, Nathalie Delherbe, Vincent J. Denef, Paul Dijkstra, Daniel D. Distel, Emiley Eloe-Fadrosh, Kirsten Fisher, Christopher Francis, Aaron Garoutte, Amelie Gaudin, Lena Gerwick, Filipa Godoy-Vitorino, Peter Guerra, Jiarong Guo, Mussie Y. Habteselassie, Steven J. Hallam, Roland Hatzenpichler, Ute Hentschel, Matthias Hess, Ann M. Hirsch, Laura A. Hug, Jenni Hultman, Dana E. Hunt, Marcel Huntemann, William P. Inskeep, Timothy Y. James, Janet Jansson, Eric R. Johnston, Marina Kalyuzhnaya, Charlene N. Kelly, Robert M. Kelly, Jonathan L. Klassen, Klaus Nüsslein, Joel E. Kostka, Steven Lindow, Erik Lilleskov, Mackenzie Lynes, Rachel Mackelprang, Francis M. Martin, Olivia U. Mason, R. Michael McKay, Katherine McMahon, David A. Mead, Monica Medina, Laura K. Meredith, Thomas Mock, William W. Mohn, Mary Ann Moran, Alison Murray, Josh D. Neufeld, Rebecca Neumann, Jeanette M. Norton, Laila P. Partida-Martinez, Nicole Pietrasiak, Dale Pelletier, T. B. K. Reddy, Brandi Kiel Reese, Nicholas J. Reichart, Rebecca Reiss, Mak A. Saito, Daniel P. Schachtman, Rekha Seshadri, Ashley Shade, David Sherman, Rachel Simister, Holly Simon, James Stegen, Ramunas Stepanauskas, Matthew Sullivan, Dawn Y. Sumner, Hanno Teeling, Kimberlee Thamatrakoln, Kathleen Treseder, Susannah Tringe, Parag Vaishampayan, David L. Valentine, Nicholas B. Waldo, Mark P. Waldrop, David A. Walsh, David M. Ward, Michael Wilkins, Thea Whitman, Jamie Woolet, Tanja Woyke, Ioannis Iliopoulos, Konstantinos Konstantinidis, James M. Tiedje, Jennifer Pett-Ridge, David Baker, Axel Visel, Christos A. Ouzounis, Sergey Ovchinnikov, Aydin Buluç, Nikos C. Kyrpides

**Affiliations:** 1 Institute for Fundamental Biomedical Research, Biomedical Science Research Center Alexander Fleming, Vari, Greece; 2 DOE Joint Genome Institute, Lawrence Berkeley National Laboratory, Berkeley, CA USA; 3 Center for New Biotechnologies and Precision Medicine, School of Medicine, National and Kapodistrian University of Athens, Athens, Greece; 4 John Harvard Distinguished Science Fellowship Program, Harvard University, Cambridge, MA USA; 5 Computational Research Division, Lawrence Berkeley National Laboratory, Berkeley, CA USA; 6 Luddy School of Informatics, Computing and Engineering, Indiana University Bloomington, Bloomington, IN USA; 7 Department of Basic Sciences, School of Medicine, University of Crete, Heraklion, Greece; 8 School of Civil and Environmental Engineering, Georgia Institute of Technology, Atlanta, GA USA; 9 Center for Microbial Ecology, Michigan State University, East Lansing, MI USA; 10 Physical and Life Sciences Directorate, Lawrence Livermore National Laboratory, Livermore, CA USA; 11 Department of Biochemistry, University of Washington, Seattle, WA USA; 12 Institute for Protein Design, University of Washington, Seattle, WA USA; 13 Howard Hughes Medical Institute, University of Washington, Seattle, WA USA; 14 Biological Computation & Process Laboratory, Chemical Process & Energy Resources Institute, Centre for Research & Technology Hellas, Thessalonica, Greece; 15 Biological Computation & Computational Biology Group, Artificial Intelligence & Information Analysis Lab, School of Informatics, Aristotle University of Thessalonica, Thessalonica, Greece; 16 Department of Electrical Engineering and Computer Sciences, University of California, Berkeley, CA USA; 17 Department of Marine Biology and Oceanography, Institut de Ciències del Mar, Barcelona, Spain; 18 Biology Department, Clark University, Worcester, MA USA; 19 AgResearch, Grasslands Research Centre, Palmerston North, New Zealand; 20 Laboratory of Environmental Microbiology, Institute of Microbiology of the Czech Academy of Sciences, Prague, Czech Republic; 21 Department of Soil Science, University of Wisconsin-Madison, Madison, WI USA; 22 Department of Biology, Boston University, Boston, MA USA; 23 Carnegie Institution for Science, Stanford, CA USA; 24 Department of Marine and Coastal Sciences, Rutgers University, New Brunswick, NJ USA; 25 Department of Biology, University of Massachusetts, Amherst, MA USA; 26 Department of Microbiology and Cell Biology, Montana State University, Bozeman, MT USA; 27 Marine Science Center, Department of Marine and Environmental Sciences, Northeastern University, Nahant, MA USA; 28 Integrative Oceanography Division, Scripps Institution of Oceanography, UC San Diego, La Jolla, CA USA; 29 School of Marine Sciences, University of Maine, Orono, ME USA; 30 Earth and Environmental Sciences, Lawrence Berkeley National Laboratory, Berkeley, CA USA; 31 Research Group Insect Microbiology and Symbiosis, Max Planck Institute for Terrestrial Microbiology, Marburg, Germany; 32 Department of Biochemistry and Molecular Biology, The Pennsylvania State University, University Park, PA USA; 33 Department of Microbiology, The University of Tennessee, Knoxville, Knoxville, TN USA; 34 School of Life Sciences, Arizona State University, Tempe, AZ USA; 35 Department of Biological Sciences, Clemson University, Clemson, SC USA; 36 School of Biotechnology and Biomolecular Sciences, UNSW Sydney, Sydney, New South Wales Australia; 37 Center for Ecosystem Science and Society, Northern Arizona University, Flagstaff, AZ USA; 38 Department of the Geophysical Sciences, University of Chicago, Chicago, IL USA; 39 Department of Microbiology and Immunology, University of British Columbia, Vancouver, British Columbia Canada; 40 Department of Biology, San Diego State University, San Diego, CA USA; 41 Department of Bacteriology, University of Wisconsin-Madison, Madison, WI USA; 42 Department of Biology, University of North Carolina at Chapel Hill, Chapel Hill, NC USA; 43 Department of Ecology and Evolutionary Biology, University of Michigan, Ann Arbor, MI USA; 44 Ocean Genome Legacy, Marine Science Center, Northeastern University, Nahant, MA USA; 45 Department of Biological Sciences, California State University, Los Angeles, CA USA; 46 Department of Earth System Science, Stanford University, Stanford, CA USA; 47 Department of Plant Sciences, University of California, Davis, Davis, CA USA; 48 Center for Marine Biotechnology and Biomedicine, Scripps Institution of Oceanography, University of California San Diego, La Jolla, CA USA; 49 Department of Microbiology and Medical Zoology, School of Medicine, University of Puerto Rico, San Juan, PR USA; 50 Lynker, Albuquerque, NM USA; 51 Department of Crops and Soil Sciences, University of Georgia, Griffin, GA USA; 52 Department of Microbiology & Immunology, University of British Columbia, Vancouver, British Columbia Canada; 53 Department of Chemistry and Biochemistry, Montana State University, Bozeman, MT USA; 54 RD3 Marine Symbioses, GEOMAR Helmholtz Centre for Ocean Research Kiel, Kiel, Germany; 55 Systems Microbiology and Natural Products Laboratory, University of California, Davis, Davis, CA USA; 56 Department of Molecular, Cell & Developmental Biology, University of California, Los Angeles (UCLA), Los Angeles, CA USA; 57 Department of Biology, University of Waterloo, Waterloo, Ontario Canada; 58 Department of Microbiology, University of Helsinki, Helsinki, Finland; 59 Marine Laboratory, Duke University, Beaufort, NC USA; 60 Department of Land Resources & Environmental Sciences, Montana State University, Bozeman, MT USA; 61 Earth and Biological Sciences Directorate, Pacific Northwest National Lab, Richland, WA USA; 62 Biosciences Division, Oak Ridge National Laboratory, Oak Ridge, TN USA; 63 Division of Forestry and Natural Resources, West Virginia University, Morgantown, WV USA; 64 Department of Chemical and Biomolecular Engineering, North Carolina State University, Raleigh, NC USA; 65 Department of Molecular and Cell Biology, University of Connecticut, Storrs, CT USA; 66 Department of Microbiology, University of Massachusetts Amherst, Amherst, MA USA; 67 School of Biological Sciences, Georgia Institute of Technology, Atlanta, GA USA; 68 Department of Plant and Microbial Biology, University of California, Berkeley, CA USA; 69 USDA Forest Service, Northern Research Station, Houghton, MI USA; 70 Department of Biology, California State University Northridge, Northridge, CA USA; 71 Université de Lorraine, INRAe, UMR 1136 Interactions Arbres/Microorganismes, INRAe-Grand Est-Nancy, Champenoux, France; 72 Department of Earth, Ocean and Atmospheric Science, Florida State University, Tallahassee, FL USA; 73 Great Lakes Institute for Environmental Research, University of Windsor, Windsor, Ontario Canada; 74 Departments of Civil and Environmental Engineering and Bacteriology, University of Wisconsin, Madison, WI USA; 75 Varigen Biosciences Corporation, Madison, WI USA; 76 Biology Department, The Pennsylvania State University, University Park, PA USA; 77 School of Natural Resources and the Environment, University of Arizona, Tucson, AZ USA; 78 BIO5 Institute, University of Arizona, Tucson, AZ USA; 79 School of Environmental Sciences, University of East Anglia, Norwich, UK; 80 Department of Microbiology & Immunology, Life Sciences Institute, The University of British Columbia, Vancouver, British Columbia Canada; 81 Department of Marine Sciences, University of Georgia, Athens, GA USA; 82 Division of Earth and Ecosystem Science, Desert Research Institute, Reno, NV USA; 83 Department of Civil & Environmental Engineering, University of Washington, Seattle, WA USA; 84 Department of Plants, Soils and Climate, Utah State University, Logan, UT USA; 85 Unidad Irapuato, Centro de Investigación y de Estudios Avanzados del IPN (Cinvestav), Irapuato, Mexico; 86 Plant and Environmental Sciences Department, New Mexico State University, Las Cruces, NM USA; 87 University of South Alabama, Mobile, AL USA; 88 New Mexico Institute of Mining and Technology, Socorro, NM USA; 89 Marine Chemistry and Geochemistry Department, Woods Hole Oceanographic Institution, Woods Hole, MA USA; 90 Department of Agronomy and Horticulture and Center for Plant Science Innovation, University of Nebraska–Lincoln, Lincoln, NE USA; 91 Department of Microbiology and Molecular Genetics, Michigan State University, East Lansing, MI USA; 92 Life Sciences Institute, University of Michigan, Ann Arbor, MI USA; 93 Departments of Medicinal Chemistry, Chemistry, and Microbiology and Immunology, University of Michigan, Ann Arbor, MI USA; 94 Division of Environmental and Biomolecular Systems, Institute of Environmental Health, Oregon Health & Science University, Portland, OR USA; 95 Animal Microbiome Analytics, Oakland, CA USA; 96 Pacific Northwest National Laboratory, Richland, WA USA; 97 Bigelow Laboratory for Ocean Sciences, East Boothbay, ME USA; 98 Departments of Microbiology and Civil, Environmental and Geodetic Engineering, Ohio State University, Columbus, OH USA; 99 Department of Earth and Planetary Sciences, University of California Davis, Davis, CA USA; 100 Max-Planck-Institute for Marine Microbiology, Bremen, Germany; 101 Department of Ecology and Evolutionary Biology, University of California Irvine, Irvine, CA USA; 102 Space Biosciences Division, NASA Ames Research Center, Moffett Field, CA USA; 103 Department of Earth Science and Marine Science Institute, University of California, Santa Barbara, CA USA; 104 Geology, Minerals, Energy and Geophysics Science Center, Menlo Park, CA USA; 105 Department of Biology, Concordia University, Montreal, Quebec Canada; 106 Department of Soil & Crop Sciences, Colorado State University, Fort Collins, CO USA; 107 Department of Forest and Rangeland Stewardship, Colorado State University, Fort Collins, CO USA

**Keywords:** Computational biology and bioinformatics, Environmental sciences, Systems biology

## Abstract

Metagenomes encode an enormous diversity of proteins, reflecting a multiplicity of functions and activities^1,2^. Exploration of this vast sequence space has been limited to a comparative analysis against reference microbial genomes and protein families derived from those genomes. Here, to examine the scale of yet untapped functional diversity beyond what is currently possible through the lens of reference genomes, we develop a computational approach to generate reference-free protein families from the sequence space in metagenomes. We analyse 26,931 metagenomes and identify 1.17 billion protein sequences longer than 35 amino acids with no similarity to any sequences from 102,491 reference genomes or the Pfam database^3^. Using massively parallel graph-based clustering, we group these proteins into 106,198 novel sequence clusters with more than 100 members, doubling the number of protein families obtained from the reference genomes clustered using the same approach. We annotate these families on the basis of their taxonomic, habitat, geographical and gene neighbourhood distributions and, where sufficient sequence diversity is available, predict protein three-dimensional models, revealing novel structures. Overall, our results uncover an enormously diverse functional space, highlighting the importance of further exploring the microbial functional dark matter.

## Abstract (web summary)

A computational approach to generate reference-free protein families from the sequence space in metagenomes reveals an enormously diverse functional space.

## Main

Metagenome shotgun sequencing has become the method of choice for studying and classifying microorganisms from various biomes^1^. With the latest advances in whole-genome sequencing technologies and the constant improvements in quality and cost efficiency, large-scale sequencing has become increasingly easier, faster and more affordable. This has led to a considerable increase in metagenomic sequencing data over the past few years, therefore making them an indispensable resource for investigating the microbial dark matter^2^.

To elucidate the genetic composition of a metagenomic sample, two major approaches are typically used, each with distinct advantages and disadvantages. In the first, sequencing reads are accurately mapped to a known, annotated set of reference genome sequences to provide a quick overview of the presence of known organisms, genes and potential functions. MG-RAST^4^ is one system that excels in this type of analysis. In the second approach, massive de novo assembly of the reads into contigs/scaffolds can provide invaluable insights into the presence of previously undescribed organisms and their genetic makeup. Recent technological advancements in assembly and binning tools^5^ have led to a significant increase in the assembled fraction of the average metagenome, coupled with a parallel exponential increase in the number of metagenome-assembled genomes (MAGs). Data management and comparative analysis systems supporting this type of data include Integrated Microbial Genomes & Microbiomes (IMG/M)^6^ and MGnify^7^.

However, both approaches share the same major limitation with respect to gene functional annotation, which relies on predicting function by homology searching against reference protein databases, such as COG^8^, Pfam^3^ and KEGG Orthology^9^. As a result, any genes predicted in assembled metagenomic data that do not map to reference protein families are typically ignored and dropped from subsequent comparative analysis. To eliminate this reliance on reference datasets and to estimate the breadth of unexplored functional diversity, referred to as the functional dark matter, an all-versus-all metagenomic comparison is required. Such a task requires considerable computational resources, yet reaching such levels of scalability remains technically challenging. Although some excellent efforts to address this issue have been recently reported^10–12^, metagenomes have not yet been comprehensively surveyed to uncover the functional dark matter.

Here we present a scalable computational approach for identifying and characterizing functional dark matter found in metagenomes. First, we identified the novel protein space present in 26,931 metagenomic datasets from IMG/M, after removing all genes with matches to the IMG database of over 100,000 reference genomes or Pfam. We next clustered the remaining sequences into protein families and explored their taxonomic and biome distributions and, where possible, predicted their tertiary (three-dimensional (3D)) structures.

## The novel protein sequence space

We initially collected all protein sequences (longer than 35 amino acid residues) from all public reference genomes and assembled metagenomes and metatranscriptomes hosted in the IMG/M platform^6^. In total, we extracted all protein sequences from 89,412 bacterial, 9,202 viral, 3,073 archaeal and 804 eukaryal genomes, resulting in a final dataset of 94,672,003 sequences. The reference genomes included in this study consisted solely of isolate genomes, not MAGs or single-amplified genomes. Similarly, for unbinned metagenomes, we extracted all predicted protein sequences from scaffolds of at least 500 bp and with lengths of at least 35 amino acids from 26,931 datasets (20,759 metagenomes and 6,172 metatranscriptomes), hereafter referred to as the environmental dataset (ED). This resulted in a non-redundant set of 8,364,611,943 predicted proteins or protein fragments. To identify the functional dark-matter component of this dataset, we first discarded any protein sequence with hits to Pfam^8^ or to any sequences from the reference genome set. The final non-redundant catalogue representing the unexplored metagenomic protein space consisted of 1,171,974,849 protein sequences (14% of the total).

## Novel protein families

**Fig. 1:**
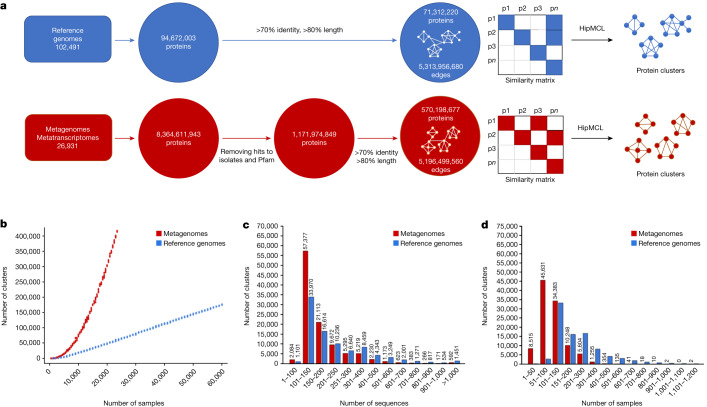
Sequence clustering overview. **a**, Clustering proteins from the reference genome (blue) and ED (red) datasets. **b**, Rarefaction curves of protein clusters for reference genome (blue) and ED (red) datasets. **c**,**d**, Bar chart visualization and comparison of cluster components per cluster for the number of sequences (**c**) and the number of genome or ED samples (**d**).

We next clustered the 1.1 billion ED proteins using a graph-based approach. For comparative purposes, we followed the same approach for the 94 million proteins from reference genomes. First, an all-versus-all similarity matrix was built for each of the two gene catalogues (that is, proteins from reference genomes and those from the ED) by calculating all significant pairwise sequence similarities. Each of the two graphs was then analysed to identify sequence-similarity-based protein clusters. For this purpose, we used HipMCL^13^, a massively parallel implementation of the original MCL algorithm^14^ that was previously developed for this scale of data and that can run on distributed-memory computers. The whole process from data retrieval to cluster generation is shown in Fig. 1a.

Although most clusters with at least 50 members (and possibly even those with at least 25 members) probably represent potentially functionally important clusters, we restricted the subsequent analysis to the larger families with at least 100 members to focus on higher-quality data as well as better candidates for predicting structures (Table 1). In total, we identified 106,198 families with at least 100 members that will be referred to as novel metagenome protein families (NMPFs) (Table 1 (right column)). For comparison, we identified 92,909 protein clusters in the corresponding set of protein clusters with at least 100 members from reference genomes. By directly comparing the two clustered sets (reference versus ED protein clusters), we observed an increase in the ED protein clusters by greater than 14-fold for clusters with at least 3 members, greater than 3-fold for clusters with at least 25 members, around a 2-fold increase for clusters with at least 50 and 75 members as well as an increase for clusters with at least 100 members (Table 1). Although the metagenome sequence space is intrinsically more fragmented compared with the reference genomes (Supplementary Methods and Supplementary Fig.  1), and a higher percentage of genes would be erroneous or incomplete (which is also one of the reasons we decided to focus all further analysis on the larger clusters), these results also suggest that much of protein sequence space remains to be explored. This is also supported by rarefaction curves generated from the ≥100-member clusters (Fig. 1b). These curves show that, as more samples become available, the cluster number increases linearly for reference genomes but exponentially, without reaching a plateau, for metagenomes.

**Table 1:** HipMCL clustering of proteins from reference genomes and metagenomes and their corresponding clusters of different protein family sizes (cumulative)

Environmental dataset					
Proteins for clustering	570,198,677				
Cluster size	≥3 members	≥25 members	≥50 members	≥75 members	≥100 members
Clusters (NMPFs)	64,149,288	1,501,861	428,910	200,075	106,198
Datasets	24,477	23,208	21,447	20,274	19,326
Scaffolds	349,547,957	71,910,494	39,593,021	27,041,114	17,280,119
Proteins	400,241,252	77,892,505	42,280,078	28,621,670	19,986,348
Percentage of proteins	70.19	13.66	7.41	5.02	3.5
**Reference genomes**					
Proteins for clustering	71,312,320				
Cluster size	≥3 members	≥25 members	≥50 members	≥75 members	≥100 members
Clusters	4,572,038	415,591	197,965	128,324	92,909
Datasets	80,896	77,611	76,294	75,145	74,134
Proteins	64,427,269	38,509,539	31,100,303	26,906,094	23,860,313
Percentage of proteins	90.34	54.00	43.61	37.73	33.46

## Biome distribution

**Fig. 2:**
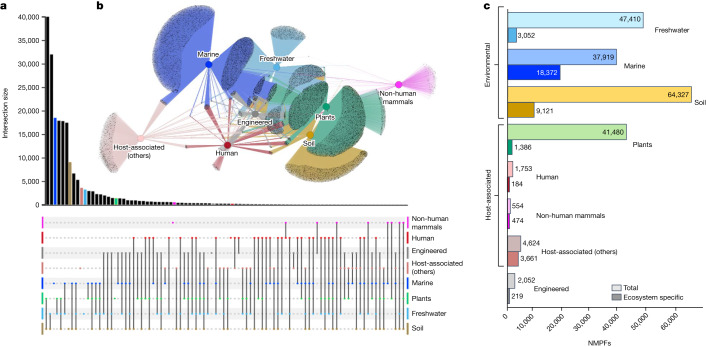
Ecosystem analysis of NMPFs. **a**, UpSet plot representation of protein clusters overlapping across the eight ecosystem types. The various intersections among different categories are represented by the chart at the bottom, with each category shown as a dot and intersecting categories connected by straight lines. The sizes of the intersection sets are represented by the vertical bar chart. Intersection sets of 15 NMPFs or higher are shown. **b**, Network representation of the protein clusters and their ecosystems. Eight ecosystem types were applied according to the GOLD ecosystem classification, represented by central, coloured nodes (hubs), whereas the grey peripheral nodes represent the protein clusters. The edges represent the protein cluster–ecosystem associations. **c**, The distribution of total versus ecosystem-type-specific NMPFs across the eight different ecosystem types.

**Extended Data Fig. 1:**
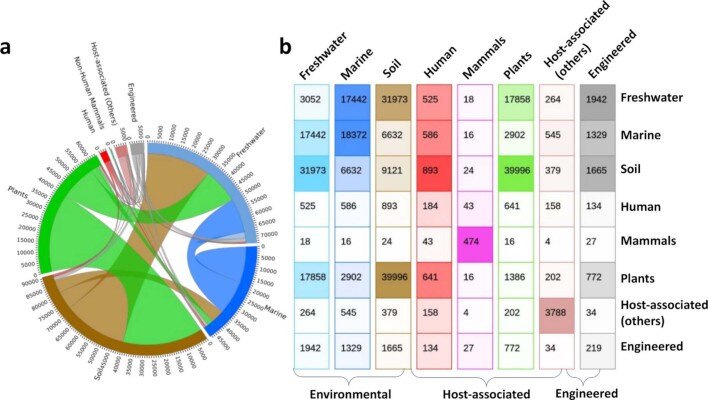
Distribution of NMPF clusters across the eight ecosystem types. (a) Circos Plot. The distribution of the ecosystems is presented in a chord-like circular diagram. The rim of the diagram represents the total size of the ecosystem types (i.e. number of NMPFs in each ecosystem), with the numbers outside the rim indicating the size scale. The intersections of categories are represented by arcs drawn between them. The size of the arc is proportional to the importance of the flow. (b) 8×8 matrix. Each cell in the matrix presents the common NMPFs in a binary combination of two ecosystems (e.g. 17,442 NMPFs are common among Marine and Freshwater ecosystems). The diagonal of the matrix displays the ecosystem-specific NMPFs. Each ecosystem column is coloured using the same colour code as Fig. 2, with the brightness of each cell being proportional to the NMPF number (brighter colour = less NMPFs).

**Extended Data Fig. 2:**
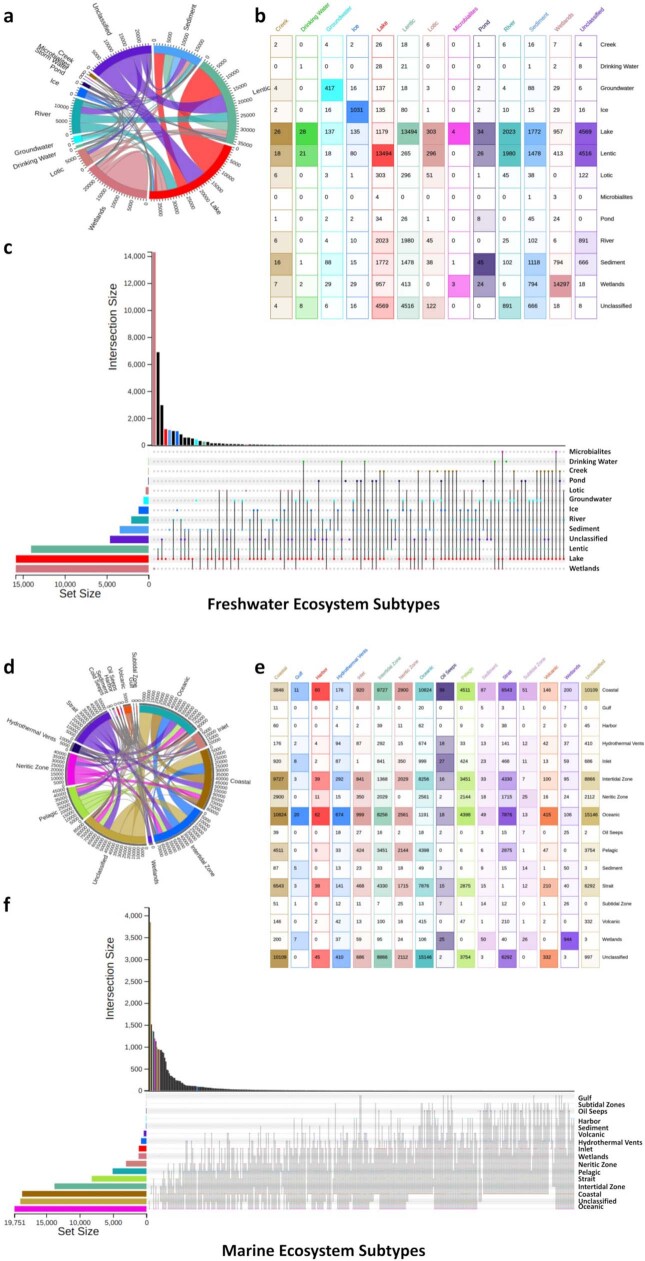
Distribution of NMPF clusters across the sub-categories of the Freshwater (top) and Marine (bottom) aquatic ecosystems. Data are shown as circos plots (a,d), colour-coded matrices (b,e) and UpSet plots (c,f).

**Extended Data Fig. 3:**
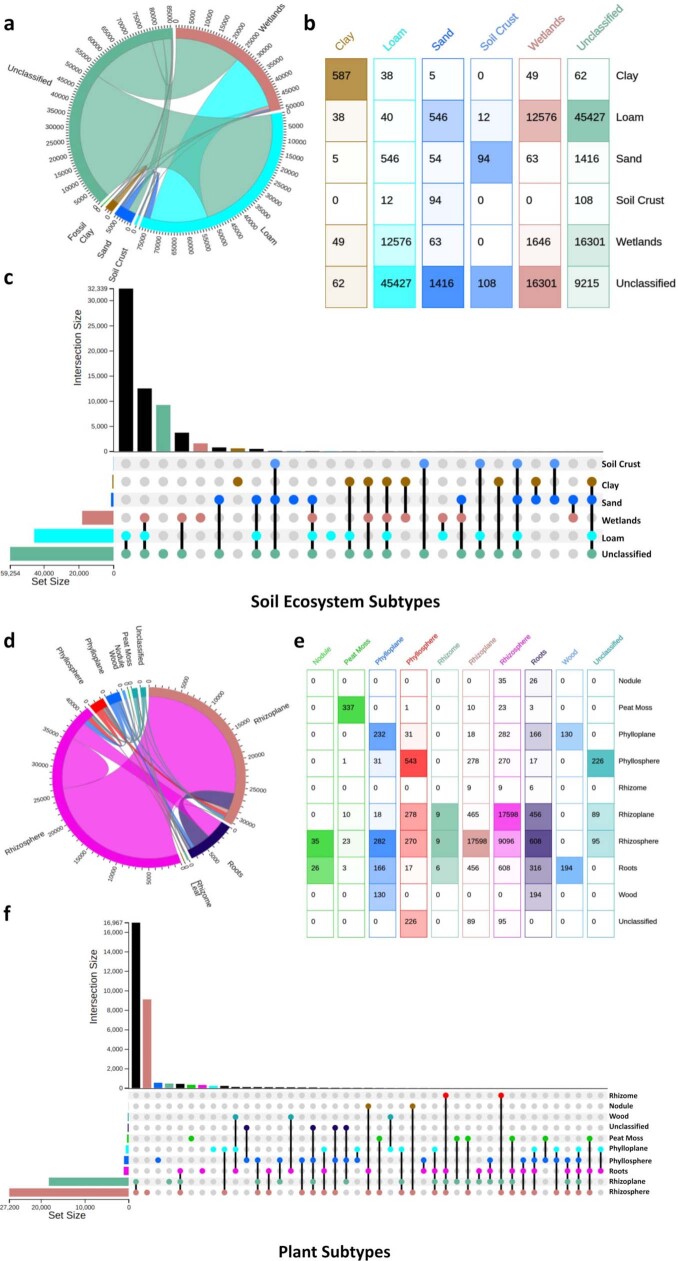
Distribution of NMPF clusters across the sub-categories of the Soil (top) and Plant (bottom) ecosystems. Data are shown as circos plots (a,d), colour-coded matrices (b,e) and UpSet plots (c,f).

**Extended Data Fig. 4:**
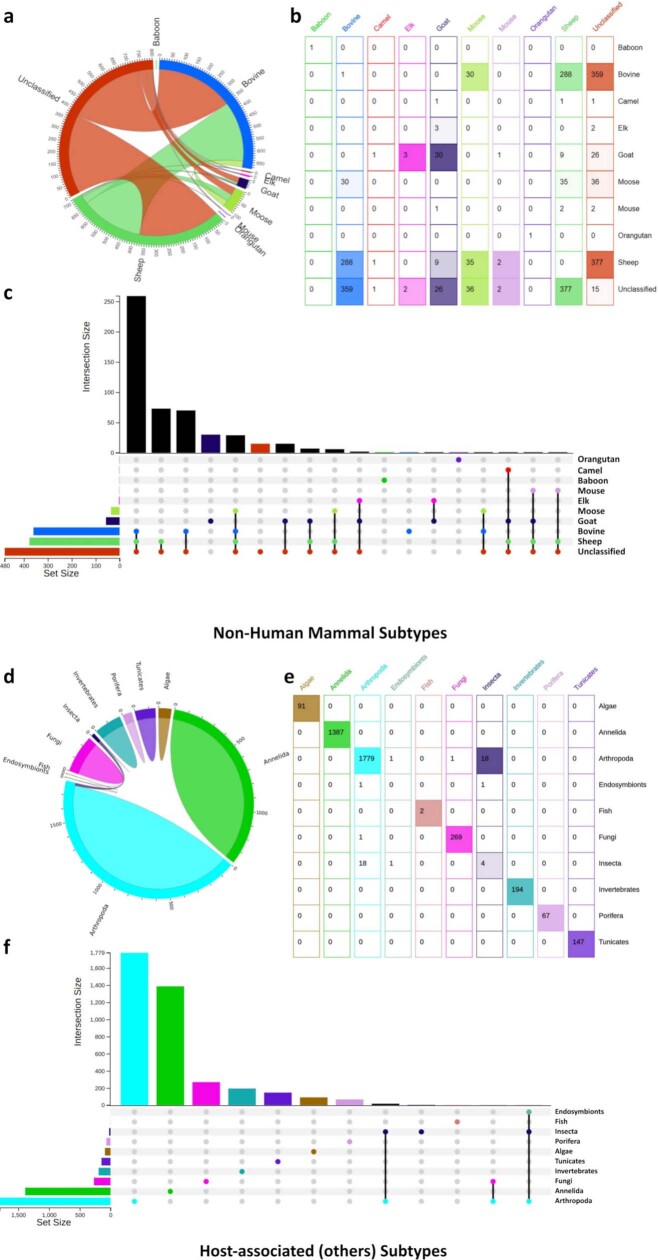
Distribution of NMPF clusters across the sub-categories of the Non-human mammal (top) and Other Host-associated (bottom) ecosystems. Data are shown as circos plots (a,d), colour-coded matrices (b,e) and UpSet plots (c,f).

**Extended Data Fig. 5:**
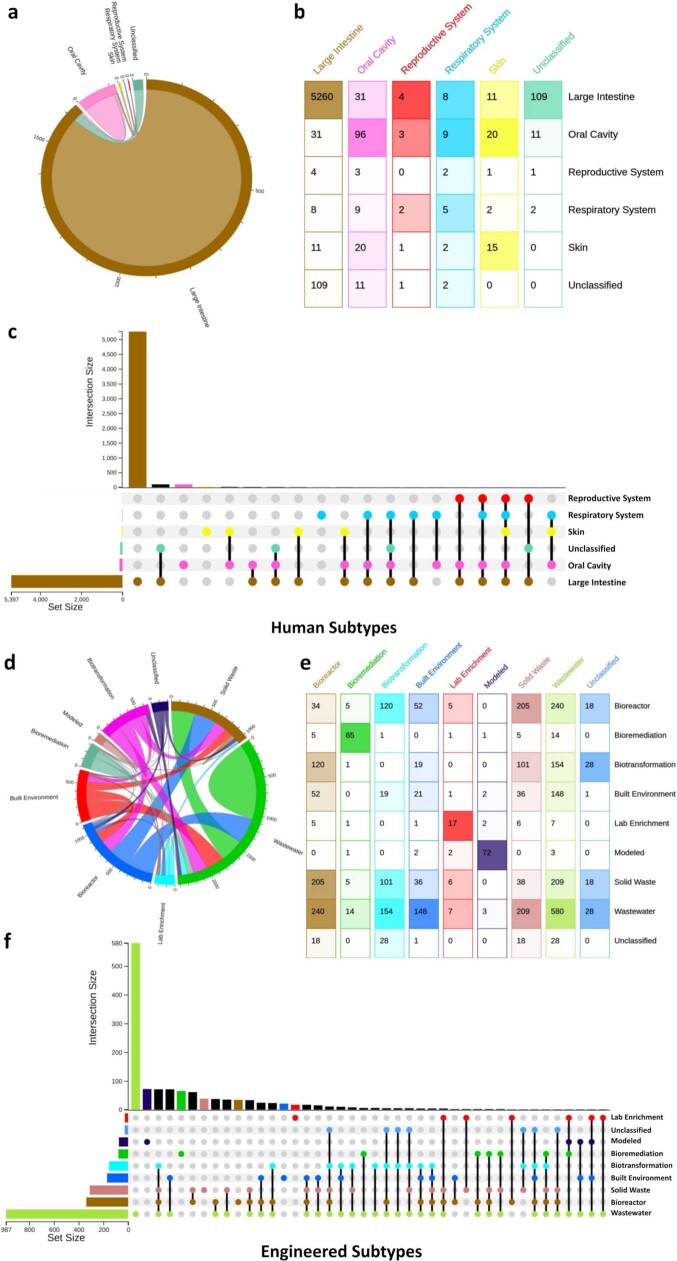
Distribution of NMPF clusters across the sub-categories of the Human tissue (top) and Engineered (bottom) ecosystems. Data are shown as circos plots (a,d), colour-coded matrices (b,e) and UpSet plots (c,f).

To determine the biome distribution landscape of the NMPFs, the corresponding metadata were collected for each sample from IMG/M^6^ using the GOLD database^15^ ecosystem classification scheme^16^ (Supplementary Table 1). The biome distribution of the NMPFs is shown in Fig. 2a,b and Extended Data Fig. 1. Here the three main GOLD ecosystems (environmental, host-associated and engineered) are further divided into eight more specific ecosystem types: freshwater, marine, soil, plants, human, non-human mammals, other host-associated and engineered. Examining the network topology, we observed minimum gene sharing within each NMPF across the three broad ecosystems, in accordance with recent observations of protein families from 13,174 metagenomes^17^, with the exception of soil/plant associations (see below). However, 7,692 NMPFs (7%) were found to have members across all of the eight ecosystem types. The properties of the top NMPFs distributed across all ecosystem types are shown in Supplementary Table 2, while the properties of the top NMPFs of each distinct ecosystem type are shown in Supplementary Tables 3 and 4. In addition to the analysis presented above, each ecosystem was further divided into subcategories for finer analysis (Extended Data Figs. 2–5 and Supplementary Fig. 2).

The largest number of NMPFs was shared between soil and plant environments (62% of the soil and 96% of plant-associated families), as would be expected due to the strong overlap of the sampling in these ecosystems (that is, most of the plant samples are from the rhizosphere) (Fig. 2a and Extended Data Fig. 3). This was followed by NMPFs shared between soil and freshwater, which could be primarily due to the assignment of wetland and sediment samples under the freshwater ecosystem classification. For the same reason, we observed a notable overlap between plants and freshwater NMPFs as well as between soil, freshwater and plant NMPFs. Conversely, only 37% of freshwater and 46% of marine NMPFs were shared with each other. Even fewer protein families were shared between ecosystem types such as human, non-human mammals and host-associated. On the other hand, a rather substantial overlap in NMPFs between human and engineered environments was observed (Fig. 2). This is not surprising, considering that engineered environments largely contain samples from human-waste-related ecosystems (such as solid waste and wastewater). Similarly, an overlap exists between freshwater and engineered environments, as well as between freshwater and host-associated types (human, non-human mammals and other host-associated), as shown in Extended Data Fig. 1. These overlaps could be indicative of phenomena such as faecal contamination of freshwater environments, leading to the co-occurrence of the same NMPFs—and, therefore, the same microbial communities—in different ecosystem types.

The percentage of ecosystem-specific NMPFs varied significantly across each of the eight ecosystem types, with the highest percentage observed for host-associated (non-human mammals) (85.6%) and host-associated (other) samples (79.2%), followed by marine (48.4%) and then soil (14.2%) samples (Fig. 2c). This is explained by the unique characteristics of the environments contained in these ecosystem categories, for example, oceanic environments of marine samples, and even more so in the case of the host-associated category, which contains a diverse array of microbiome hosts with significant biological differences (for example, arthropods and annelids) (Extended Data Fig. 4). In contrast to marine samples, freshwater samples had a very small percentage of ecosystem-type-specific families, mostly due to a large number of wetland and sediment samples with strong associations with soil, as did the plant/rhizosphere-related samples with soil samples (Fig. 2a).

Finally, to investigate the ecosystem distribution of NMPFs, the ecosystem prevalence of the most-abundant NMPFs of each ecosystem type was evaluated. The prevalence of each NMPF in an ecosystem (for example, freshwater) was calculated as the number of family ecosystem-associated datasets over the total number of ecosystem-associated datasets in the study (Supplementary Table 3). Despite the existence of NMPFs strongly associated with a particular ecosystem type (for example, >80% of NMPFs), their prevalence in the overall datasets associated with said ecosystems was rather low, with most NMPFs distributed across 5–20% of the samples associated with each ecosystem type. The only exception was the non-human-mammal-associated NMPFs, for which prevalence reached up to around 45% of the total non-human mammalian datasets.

## Taxonomic distribution

Taxonomic assignment of NMPFs was performed on the basis of the available taxonomy information of the corresponding scaffolds in IMG, for each member of the clusters^18^. In cases in which no such annotation was available, we used a combination of additional approaches to computationally infer the taxonomy of the scaffolds (Methods). Of the total 17,280,119 IMG/M scaffolds containing the NMPF members, 8,049,154 were classified as Bacteria, 382,761 as Archaea, 1,184,393 as Eukaryota and 1,406,588 as viruses, leaving 6,257,223 as unclassified.

**Fig. 3:**
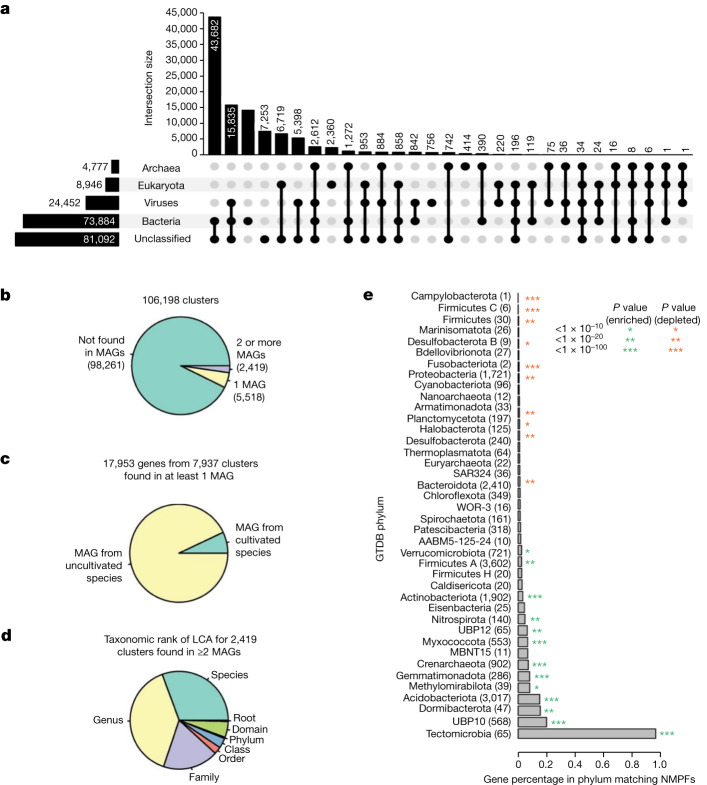
Taxonomic composition and occurrence of NMPFs in bacterial and archaeal MAGs. **a**, UpSet plot showing the domain-level taxonomic distribution of novel protein clusters. The total size of each taxonomic category is represented through the horizontal bar chart on the left. The intersections among categories are represented by the chart at the bottom, with sizes of the intersections represented by the vertical bar chart at the top. **b**,**c**, We determined whether NMPFs were found on scaffolds from the GEM catalogue (**b**) and whether they were found on scaffolds from one or more cultivated species (**c**). **d**, The taxonomic rank of the lowest common ancestor (LCA) for 2,419 clusters found in at least 2 MAGs. **e**, The percentage of genes matching a cluster from MAGs assigned to different phyla. The asterisks indicate significant *P* values from a hypergeometric test. Green, clusters enriched in the phylum; red, clusters depleted from the phylum. The number of genes matching clusters is indicated in parenthesis next to the phylum name.

**Extended Data Fig. 6:**
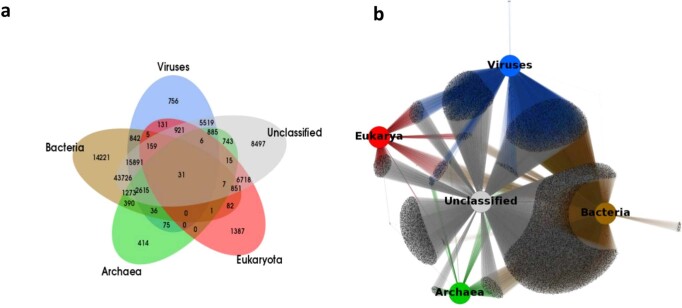
Distribution of NMPF clusters across different taxa (bacteria, archaea, eukarya, viruses, and unclassified). (a) Venn Diagram, displaying the intersections among the different taxonomy categories. (b) Network representation of the protein clusters and their taxonomic assignments. The taxa are represented by central, coloured nodes (hubs) whereas the grey peripheral nodes represent the protein clusters.

The taxonomic distribution of the NMPFs, on the basis of their corresponding scaffold taxonomic assignment, is shown in Fig. 3a and Extended Data Fig. 6. The majority of protein families included sequences with multiple taxonomic assignments (such as bacterial and unclassified, or bacterial and viral). The largest category consisted of families with bacterial/unclassified sequences, followed by viruses/unclassified and bacteria/viruses. A much smaller group of families was assigned to Eukarya and even fewer families to Archaea. Finally, 7,253 clusters had no taxonomic information at all.

As no reliable de novo eukaryotic gene predictor exists for unbinned metagenomes^19^, a lot of sequences may come from eukaryotic scaffolds that may contain translation errors (such as mistranslated introns). However, analysing the contents of these clusters (Supplementary Methods) showed that their majority include proteins from Bacteria and Archaea alongside Eukarya, with very few NMPFs containing only eukaryotic sequences (Supplementary Data 6). Moreover, more than half of these clusters are validated by metatranscriptomic data. These two observations supported the quality of the eukaryotic-containing NMPFs.

Subsequently, we evaluated whether any of the NMPF proteins (and their corresponding families) were found in any of the recently identified MAGs from the Genomes from Earth’s Microbiomes (GEM) catalogue^20^. Specifically, we examined whether any of the scaffolds containing genes of the NMPFs were binned in any of the 52,515 MAGs of the GEM catalogue. This revealed that only 17,953 genes, coming from 7,937 NMPFs (7.4% of total) (Fig. 3b,c), were found within the GEM catalogue, of which the vast majority (93%) was from uncultured species. For those families that were present in two or more MAGs, we noticed a strong narrow taxonomic distribution, with more than two-thirds being restricted to a single species or genus, and only a very small number found across multiple families, classes or phyla (Fig. 3d). NMPFs were found to be statistically enriched in several phyla common in soil environments (for example, Gemmatimonadota, Acidobacteriota, Crenarchaeota and Myxococcota) and statistically depleted from several phyla found in humans and other host-associated environments (Firmicutes, Proteobacteria and Bacteroidota; Fig. 3e). Taken together, these results reveal that a significant fraction of functional diversity remains taxonomically orphan despite improvements in sequencing throughout and large-scale MAG reconstructions.

## Metadata distribution

**Extended Data Fig. 7:**
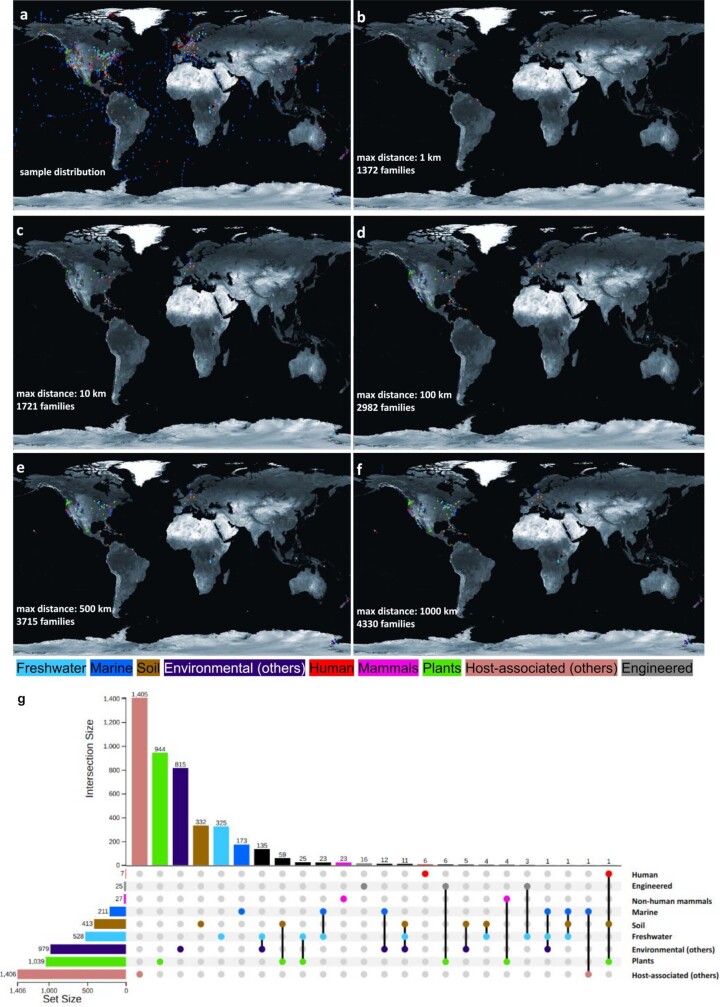
Geographical distribution of the ED samples and NMPFs. (a) Locations for all ED samples in the study with available geo-location metadata (Longitude and Latitude). (b-f) Distribution of geographically-isolated NMPF clusters, based on a cut-off distance of 1, 10, 100, 500, and 1000 Km. In all cases, dots are coloured based on the ecosystem type (blue: marine, cyan: freshwater, brown: soil, purple: other environmental, green: plants, red: human, magenta: non-human mammals, salmon pink: other host-associated, grey: engineered). (g) UpSet plot showing the distribution of the geographically-isolated NMPF clusters, based on a cut-off distance of 1000 Km (as shown in panel f). Map panels were created using data from the Natural Earth dataset (www.naturalearthdata.com).

We next examined the geographical distribution of NMPFs (Extended Data Fig. 7). A very small number of families (1,372; 1.3%) was found to have limited geographical distribution (within 1 km), and this number only moderately increased (4,330; 4%) when we allowed for a maximum distance of 1,000 km. Most of these families were found in plant, soil and freshwater ecosystems. A very small number of these families included members found in marine ecosystems or human samples as expected from the higher microbial dispersal in these ecosystems (Extended Data Fig. 7f,g).

The majority of NMPFs (64,186 or 60.44%) comprised a mixture of proteins from both metagenomes and metatranscriptomes, further validating their existence, whereas 38,292 (36.06%) of NMPFs contained proteins found exclusively in metagenomes and 3,720 (3.50%) of NMPFs contained proteins found only in metatranscriptomes (Supplementary Table 5). The percentage of families containing members from both metagenomes and metatranscriptomes steadily decreased along with the number of members per family. NMPFs found in both metagenomes and metatranscriptomes also had the widest sample distribution, that is, the clusters were found in the largest numbers of samples (Supplementary Table 6). The majority of these clusters was classified in environmental ecosystems (soil and, to a lesser extent, marine and freshwater samples) and primarily contained bacterial and unclassified sequences.

To estimate the distribution of novel protein clusters among the environmental sequencing data, we compared the number of novel proteins, extracted from each scaffold and used in this study, against the total number of genes/proteins in the respective scaffold. Most analysed scaffolds (13,407,728 or 77.59%) contained both novel and known genes (top 20 scaffolds; Supplementary Tables 7–11). A comparison of novel versus the total number of genes in these scaffolds revealed that the size of the scaffold or total number of genes per scaffold was not correlated with the number of novel genes. The largest scaffold in our study (5,123,848 bp, 4,302 genes) contained only one novel sequence. Generally, the largest scaffolds in the study contained only a limited number of novel sequences and originated from bacterial or unclassified metagenomic samples (Supplementary Table 9). Conversely, the scaffolds with the most novel sequences were of variable length (and gene count) and mostly originated from viruses (Supplementary Table 10).

**Extended Data Fig. 8:**
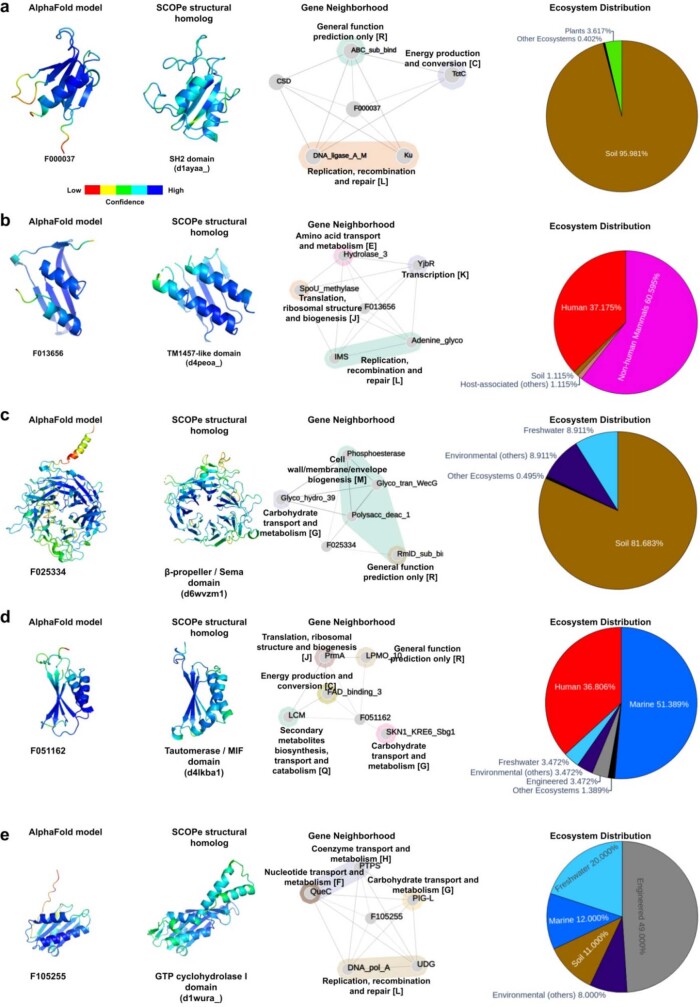
Functional annotation of NMPFs with remote structural homologues. Five example NMPFs (a-e) are shown. Annotation is performed using using structural information (left), gene co-occurrence analysis (middle), and ecosystem distribution (right). Each of the NMPFs has a high-quality 3D model with at least one remote structural homologue to SCOPe. The NMPFs’ 3D models, produced with AlphaFold, and the structures of the SCOPe domains are rendered in the same orientation and coloured based on their per-residue structure confidence (pLDDT for AlphaFold models and inverse B-factor for experimental structures). The gene neighbourhood of each NMPF is presented in the form of an association network; with nodes representing gene products (the NMPFs and their adjacent genes that encode Pfam domains) and edges representing co-occurrence in the same sequencing scaffold. Pfam domains are further grouped using their associated COG functional categories as annotation. Finally, the NMPFs’ associated ecosystems are presented in pie charts. Ecosystems with a <1% presence in the NMPFs are summed into the category “Other ecosystems”.

**Extended Data Fig. 9:**
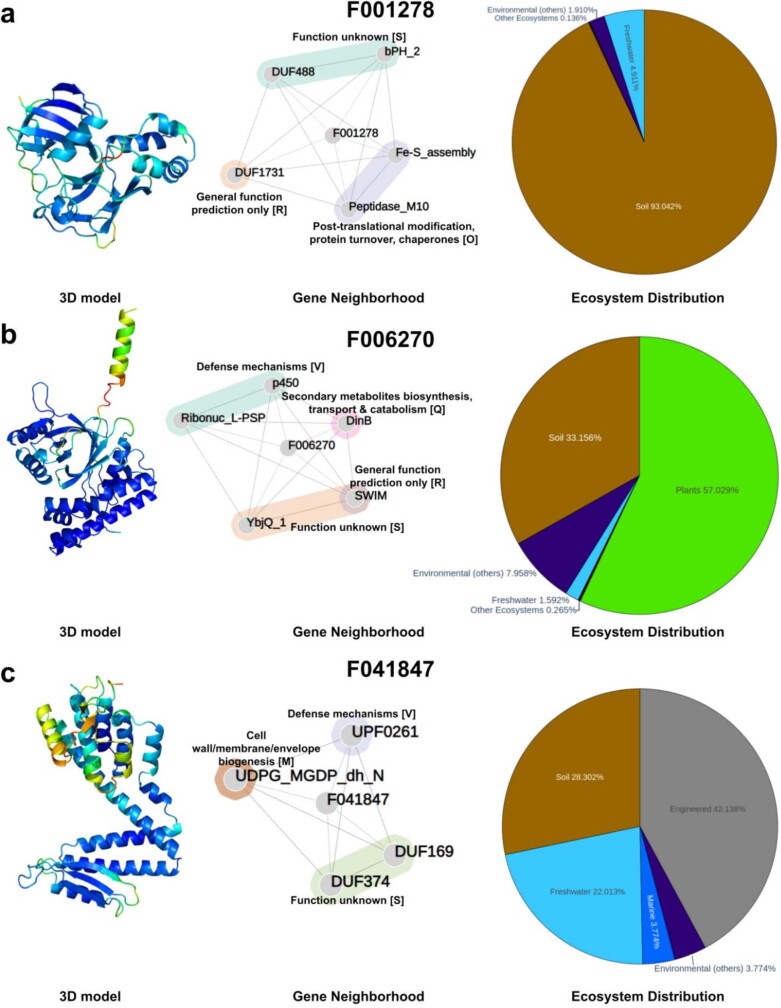
Putative functional annotation of NMPFs with potential novel structural folds. Three example NMPFs (a-c) are shown. The produced AlphaFold 3D model (left), gene co-occurrence analysis (middle) and ecosystem distribution (right) are given. 3D models are coloured based on their per-residue structure confidence (pLDDT). The gene neighbourhood of each NMPF is presented in the form of an association network; with nodes representing gene products (the NMPFs and their adjacent genes that encode Pfam domains) and edges representing co-occurrence in the same sequencing scaffold. Pfam domains are further grouped using their associated COG functional categories as annotation. Finally, the NMPFs’ associated ecosystems are presented in pie charts. Ecosystems with a <1% presence in the NMPFs are summed into the category “Other ecosystems”.

As the majority of novel proteins (14,185,414 sequences) was located next to known genes, we investigated the co-occurrence of NMPFs with neighbouring genes assigned to the same Pfam family. Additional annotation was obtained by mapping each NMPF’s co-occurring Pfam domains to their corresponding COG functional categories; this can be used to provide further information on each family’s gene neighbourhood. The distribution of NMPFs across functional categories is given in Supplementary Figs. 3–5. Conserved gene neighbourhoods suggest functional coupling^21^ and can therefore be used to provide additional lines of information for putative function prediction. Accordingly, family F004468 was found to co-occur with ribosomal proteins in 78% of scaffolds (that is, in 118 scaffolds out of the 151 scaffolds in which it was encoded), suggesting that it has a translation-related function. Similarly, family F021307 was found within a probable chloroplast ribosomal protein operon in 67% of encoding scaffolds. In total, 7,625 NMPFs were found to have greater than 50% co-occurrence with specific Pfams, while 585 families had greater than 90% co-occurrence with a Pfam family (Supplementary Data 1). These associations can also be used to predict a functional role for NMPFs; a few examples are given in Extended Data Figs. 8 and 9, in which the gene neighbourhoods of selected NMPFs are presented as association networks, combined with functional annotation from COG.

## Structural distribution

**Fig. 4:**
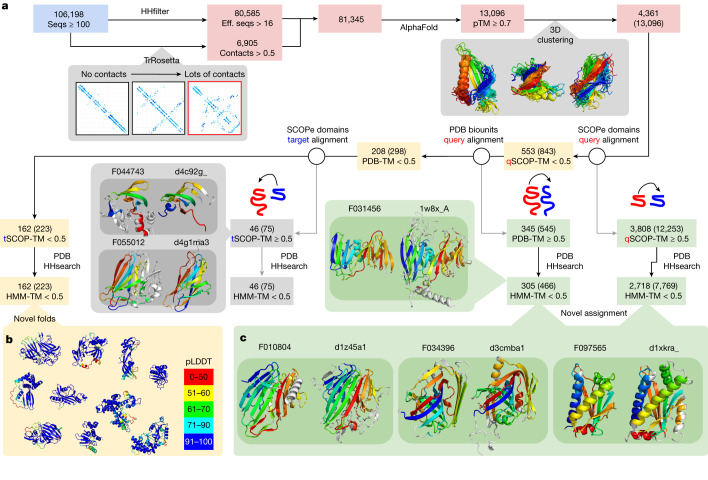
Structural characterization of the NMPFs. **a**, Protein clusters with at least 16 effective sequences (eff. seqs) or many contacts were submitted to AlphaFold. The results were filtered to include structures with high predicted confidence (pTM ≥ 0.70), which were then clustered on the basis of pairwise TM-score calculation. All of the subsequent steps of the workflow display the number of unique clusters followed by the total number of NMPFs in parentheses. As filtering was performed at the NMPF level, only the numbers in parentheses will sum, as it is possible for members of the same cluster to fall on different sides of each TM-score filtering step. Each predicted structure was aligned against SCOPe domains. Models with no hits to SCOPe were further aligned and filtered if there were any hits to full PDB assemblies or one of the SCOPe domains aligned to at least 50% of the predicted structure. The domains (from SCOPe matches) or multi-domain (from PDB matches) were further screened using HHsearch against the PDB. The PDB of the top hit was compared to the prediction. **b**, Models with no significant hits to either SCOPe or PDB were considered to be potential novel folds. pLDDT, per-residue confidence score. **c**, Models with hits to either SCOPe domains or PDB biological assemblies with no significant HHsearch hits (HMM-TM-score < 0.5) were considered to be novel assignments.

Recent breakthroughs in protein structure prediction^22^ have enabled fast and accurate structural characterization of protein sequences. Metagenomic sequences have been shown to represent a particularly rich source for the discovery of novel structures^23,24^. Here we ran AlphaFold2^22^ on NMPFs with at least 16 diverse sequences, or where TrRosetta^25^ predicted a well-structured protein (Methods). The results are summarized in Fig. 4a. Out of the 81,345 NMPFs that met the above criteria, 80,585 3D models were predicted, with 13,096 NMPFs having a high confidence (predicted TM (pTM) score > 0.700) prediction. The pTM-score integrates both the predicted confidence per position and the predicted alignment error (pAE) for every pair of positions, indicating the confidence of domain–domain orientations.

On the basis of structural clustering, these high-confidence predictions represented 4,361 unique structures. To examine the novelty or functions of these structures, we compared them to experimentally determined structures from SCOP-Extended (SCOPe)^26^ and assemblies from the Protein Data Bank (PDB)^27^. In total, 3,808 structures (12,253 NMPFs) had a significant structural overlap with at least 1 SCOPe domain (TM-score > 0.5). Of these, 2,718 (7,769 NMPFs) had a non-trivial hit, indicating that 62.3% of high-quality predictions had some similarity to at least one SCOPe domain or PDB assembly.

These novel assignments, based on structural similarity, can now be used for functional prediction of the corresponding sequences. A few examples are shown in Fig. 4c. For example, family F034396 had no hits to the PDB using HHsearch (top hit of *e*-value = 12), yet a strong hit to the PDB using a structural search of the SCOPe domain d3cmba1 (TM-score = 0.69), with the function of acetoacetate decarboxylase. Other examples with no HHsearch hits (*e*-value > 10), yet strong structural hits included F010804-d1z45a1 (TM-score = 0.73, galactose mutarotase) and F097565-d1xkra_ (TM-score = 0.73, chemotaxis). We stress that these cases should be treated as informed predictions that require experimental validation, as the same fold does not always correspond to the same function. A full list is provided in Supplementary Data 2. However, some validation and additional functional annotation can be performed by combining these novel assignments with other NMPF metadata, such as gene co-occurrence. A few examples are given in Extended Data Fig. 8.

To confirm that the remaining 553 proteins with no SCOPe hit were novel folds, a more thorough search was performed against all PDB biological assemblies, including all possible chain permutations. In total, 345 models had a hit to at least one PDB entry, of which 305 represented additional novel assignments. The remaining 208 were processed for further filtering, removing predictions of which 50% of the structure matched a SCOPe domain. Finally, 162 folds and/or domain–domain orientations from 223 NMPFs were identified as novel (Fig. 4b). A complete list of these folds is provided in Supplementary Data 3.

Although the absence of any significant structural homology precludes the reliable functional annotation for these novel folds, some hints towards their potential function can be gleaned from their associated metadata. Characteristic examples are given in Extended Data Fig. 9, showcasing the gene neighbourhood and ecosystem metadata of three NMPFs with novel structural folds.

## Discussion

Arguably, the best approach for estimating and exploring microbial functional diversity is through systematically cataloguing and exhaustively characterizing sequence-diversity space. Over the past three decades, genome sequencing of hundreds of thousands of cultured microbial strains has enabled unprecedented growth and characterization of this sequence space, revealing that sequencing efforts targeted to maximize phylogenetic diversity can lead to further discoveries and growth of currently known protein family diversity^28^. Although the exploration of the corresponding sequence-encoded functional diversity is lagging substantially^29^, the explosion in the number of identified novel protein families has, to a great extent, been accompanied by an increase in targeted functional characterization of some of those families, particularly in areas of important biotechnological applications such as the discovery of new CRISPR–Cas genes and systems^30^. The advent of metagenomics has further fuelled the rush to discover new enzymatic activities by unearthing a hidden treasure trove of untapped sequence information. Yet, aside from generating for-the-first-time important habitat-specific environmental gene catalogues^31^, most explorations of exponentially growing metagenomic sequence space have focused on expanding the diversity and characterization of previously known protein families^32^.

To alleviate this limitation and pioneer global insights into the extent of novel sequence space and, by effect, functional diversity across the realm of sequenced biomes, we have amassed close to 27,000 publicly available assembled metagenomic and metatranscriptomic datasets. From these datasets, we generated the NMPF catalogue, consisting of 106,198 metagenome protein families of 100 members or more with no sequence similarity to genes from reference microbial genomes or Pfam entries. Although these families represented a mere duplication over the number of families generated from more than 100,000 reference genomes integrated into IMG from all domains of life, far greater increases were observed in the families containing more than 25, 50 or 75 members, strongly suggesting that extensive sequence and functional diversity remains untapped. We anticipate that this diversity in unexplored microbial protein space will continue to increase over the next several years as more novel environmental samples are sequenced.

Although a much smaller number of metatranscriptomes was available for this analysis (4,739; 17.6%), we observed that the majority of NMPFs (60%) comprised proteins encoded by genes identified in both metagenomes and metatranscriptomes, indicating that most of those genes are actively expressed, further supporting the validity of those clusters. The clustering quality was also supported by the observation that 92% of clusters had members spanning 50 samples or more, while 50% of the clusters were from proteins distributed across 100 samples or more (Fig. 2d).

The identification of 7.5% of the NMPFs on the recently reconstructed MAGs of the GEM catalogue indicates that, as we continue to access the genetic content of uncultured microbial diversity, an increasing number of taxonomically orphan novel protein families will become taxonomically assigned, an important step towards their functional and ecological characterization.

There are several limitations underlying the metagenomic data and methodology used in this study. One limiting factor to consider is the short size (shorter than 5 kb) of the majority of scaffolds used in this study. However, note that, due to the required alignment coverage of at least 80%, potentially truncated sequences have to be sufficiently complete to cluster with full-length sequences (defined as located in the middle of longer scaffolds). This requirement has largely precluded the enrichment of NMPFs with fragmented proteins. However, even in the case of NMPFs with a high percentage of these suspect sequences, the clusters are found to produce stable 3D models (often with high structural quality, as evidenced by pLDDT and pTM scores), many of which have structural homologues to SCOPe domains. As a result, families containing such sequences could potentially represent protein fragments or protein domains that form parts of multi-domain sequences, or components in multimeric complexes. An additional potential limitation is the inclusion of eukaryotic sequences in the sequence dataset, which may introduce errors in the analysis. Yet, as shown (taxonomic distribution; Supplementary Methods), the contributions from eukaryotic scaffolds are relatively small, and the majority of the associated NMPFs also contain data from metatranscriptomes and/or prokaryotic taxa in sequence alignments, supporting their validity. However, until reliable eukaryotic gene predictors for metagenomes become available, eukaryotic, as well as unclassified NMPFs and sequences should be handled with care.

Overall, as more metagenomic data become available, an increasing diversity of sequences will be incorporated into NMPFs, which will then enable the generation of a much higher number of high-confidence structures, and therefore further increase the numbers of assignments to known structures as well as uncover novel folds. The identification of NMPFs opens new paths for structural genomics and challenges for fold recognition and the exploration of microbial dark matter.

## Methods

### Data collection and filtering

All publicly available metagenomes, metatranscriptomes and reference genomes were retrieved from the IMG/M database^6^ (database release, July 2019). Low-complexity regions were removed with the use of the tantan application^33^. In total, we extracted all protein sequences from 89,412 bacterial, 9,202 viral, 3,073 archaeal and 804 eukaryal genomes. This corresponded to 87,084,214 bacterial, 221,027 viral, 2,464,569 archaeal and 4,902,193 eukaryotic non-redundant proteins, resulting in a final dataset of 94,672,003 sequences. Pfam hits (v.31) were detected with the use of the hmmsearch tool (HMMER v.3.1 package)^34^ using the default trusted cut-off. Hits to proteins from reference genomes were calculated using LAST^35^. We considered a hit to be any aligned sequence at >30% identity over 70% of its length (bidirectionally between the query and the subject). A detailed workflow of the sequence selection, filtering and analysis procedure is provided the Supplementary Methods. Α full summary of the sequences contained in each metagenome and metatranscriptome dataset, including hits to Pfam and reference genomes, sequences used in clustering (see below) and the remaining unannotated sequences, is provided in Supplementary Data 4.

### Sequence clustering and analysis

Sequence clustering was performed using the HipMCL algorithm with inflation parameter 2.0 using identity scores as the input. HipMCL was chosen over other clustering solutions owing to its scalability and parallelization capabilities, as well as its ability to efficiently cluster very large datasets (Supplementary Methods). Before clustering, the all-versus-all pairwise alignments were calculated using LAST (70% sequence identity, 80% alignment coverage). The reference genome graph consisted of 71,312,220 nodes (proteins) and 5,313,956,680 edges (pairwise similarities). The graph for the ED proteins consisted of 570,198,677 nodes and 5,196,499,560 edges. Notably, during the similarity matrix construction, 23,359,783 (~24.67%) out of the 94,672,003 reference proteins and 601,776,172 (~51.34%) out of the 1,171,974,849 ED proteins remained as singletons. Using 2,500 compute nodes (170,000 compute cores) of the NERSC Cori supercomputer (Intel KNL partition), HipMCL clustered the reference protein graph in 24 min and the ED protein graph in 3 h and 20 min.

Notably, for this task, we explored several graph- and sequence-based methods such as CD-HIT^36^, UCLUST^37^, kClust^38^ or Louvain^39^ that could not perform at this scale as well as MMSeq2-linclust^40^ and SPICi^41^, which were previously proposed to scale at this data size but none performed sufficiently well for the scope of this work. A comparison among these different clustering strategies is provided in the Supplementary Methods. With regard to sequence identity, we set the cut-off at 70% to achieve a compromise between sensitivity and specificity. Although other resources have used more stringent parameters (such as MGnify^6^, in which sequence clustering is performed with a 90% sequence identity cut-off), focusing on specificity, we have chosen a more sensitivity-based approach, yet not overly sensitive, to avoid artifacts produced by noise, multi-domain effects and false positives. Regarding alignment coverage, the choice of a high-coverage threshold (80%) ensures generating better-quality sequence alignments and avoiding potential artifacts, such as partial hits involving significantly truncated/incomplete genes, pseudogenes and chimeric sequences. The overall quality of the final dataset is further improved by considering MCL clusters with 100 members or more, ensuring the preclusion of spurious gene products. An analysis of how the clustering cut-off values (specifically sequence identity) may influence the quality of the resulting clusters is also provided in the Supplementary Methods.

For each cluster, a multiple-sequence alignment (MSA) was calculated using Clustal Omega^42^. The resulting MSAs were then filtered to produce seed (non-redundant) alignments using a script written in Python and the ProDy/Evol and Biopython modules^43^ (90% sequence identity, 75% alignment coverage) (Supplementary Methods). All of the subsequent analysis steps, including the calculation of length distributions, HMM profile training and 3D structure predictions, were performed using the generated seed MSAs. HMM profiles were generated using the hmmalign utility of HMMER v.3.1 suite^34^. The representative consensus sequences for each cluster were calculated with Biopython (Supplementary Methods). Consensus sequences were searched against the whole Pfam-A^8^ database (v.33) HMM profiles with HMMER (inclusion thresholds: 7.0 total, 5.0 domain), as well as against the reference genomes using BLAST^44^ (30% identity, 50% coverage bidirectionally). Initial NMPFs of ≥100 members (113,752 clusters from 19,473 datasets with 20,211,137 scaffolds and 21,260,914 proteins) were processed for additional filtering with more stringent criteria to remove sequences with even weak similarities to Pfam-A models or genes from reference genomes, based on their calculated consensus profile sequences. This resulted in a more-stringent set of 106,198 clusters. Clusters of which the consensus sequence was found to have a hit to either Pfam-A or a reference genome were removed. A complete summary of the clustering procedure for each ED dataset is provided in Supplementary Data 3. Plot distributions were computed using R and the R/ggplot2^45^ package. For all calculations regarding NMPF sequence length, the average sequence length of each cluster’s seed MSA was calculated and used.

### Verification of protein family novelty

Additional searches were performed against the reference proteomes of RefSeq (November 2021 release) using the NMPF clusters’ consensus sequences and HMM profiles with LAST (matrix: BLOSUM62, gap open: 11, gap extend: 2) and HMMER (inclusion thresholds: 7.0 total, 5.0 domain), respectively, and applying a 70% alignment coverage cut-off. These searches showed that 5,111 clusters (around 5% of the total) obtained positive hits against 134,273 RefSeq sequences. Taxonomic annotation of these sequences showed that 75,215 (56.01%) of the positive hits were eukaryotic, 46,793 (34.85%) were bacterial, 9,296 (6.92%) were archaeal and 2,905 (2.16%) were viral, with 64 sequences having no taxonomic annotation. About half (70,554 or 52.54%) of the hits were published from 2020 onwards, with the majority of those matching genes from MAGs. Cross-examination of the RefSeq hits with UniProt (release 2021_04) records showed that 31,242 (23.27%) of the RefSeq sequences were mapped to 33,628 UniProtKB entries (453 SwissProt and 33,175 TrEMBL); these sequences correspond to only 32 NMPF clusters. The rest of the RefSeq sequences (103,031) were contained in the UniParc archive of UniProt and had no annotation evidence (either manual or automatically generated). The NMPF clusters were searched against Pfam-B, a non-annotated, computationally generated dataset of alignments for sequences not covered by Pfam-A. This resulted in 8,313 unique clusters with positive hits against 5,310 Pfam-B. Finally, the positive hits against the searches of each dataset were compiled and compared. In total, 12,846 clusters had a positive hit to RefSeq, Pfam-B or both, while the rest of the clusters (93,352) had no hits. Finally, all NMPF sequences were searched against AntiFam^46^ v.6.0, a collection of HMM profiles designed to detect potential spurious protein sequences, pseudogenes and false protein translations. Only 43 sequences were identified, with low-score hits and low alignment coverage (<50%) to two AntiFam profiles.

### Ecosystem and taxonomic annotation of protein clusters

NMPF clusters were annotated with available environmental and taxonomy metadata through their associated environmental datasets. In the case of environmental metadata, the GOLD^15^ ecosystem classification scheme^14^ was used to organize datasets into ecosystem groups (such as freshwater, marine, soil, host-associated); each protein cluster was then assigned to one or more ecosystems on the basis of the ecosystem information of the ED samples in IMG on which the protein sequences were found. In addition to GOLD, the Environment Ontology (ENVO)^47^ and Earth Microbiome Project Ontology (EMPO)^48^ were considered as potential alternative classification systems. Mapping of ED samples and NMPFs to ENVO and EMPO was performed using the metadata of the GOLD biosample project associated with each ED sample. The ecosystem assignments for all three classification systems are presented in Supplementary Data 5. Ultimately, the GOLD classification was used, as it was found to offer the most diverse options and classified all ED samples and NMPFs. From the 19,326 environmental samples that included the NMPFs, 14,540 (75.24%) were environmental (for example, soil, freshwater), 3,867 (20.01%) were host-associated (for example, human, plants) and 919 (4.76%) came from engineered environments (for example, wastewater, industrial wastes).

In a similar manner, the initial taxonomic annotation for the clusters was performed using the NCBI taxonomy information of the scaffolds contained in each IMG/M dataset, where available. Note that the majority of the scaffolds used in this study were too short and therefore remained unclassified taxonomically. Furthermore, there is very little information on the taxonomy of viral scaffolds. To alleviate these issues, annotations for scaffolds >5 kb that had previously been identified as viral and included in version 3.0 of IMG/VR^49^ were used. Moreover, scaffolds 1–5 kb in length were analysed using DeepVirFinder (v.1.0)^50^, and the generated *P* values were subsequently converted to *q* values using the R package qvalue^51^ to obtain estimates of the false-discovery rate. Scaffolds with *q* ≤ 0.001 were retained as putative viral scaffolds. Unclassified scaffolds were further analysed using two eukaryotic sequence detection tools, Whokaryote^52^ and EukRep^53^. Furthermore, the NMPF clusters were searched against the Tara Oceans collection of eukaryotic MAGs^54^. Finally, all remaining unclassified scaffolds were taxonomically assigned using the MMseqs2 taxonomy tool^40,55^, performing six-frame translation searches against UniRef50^56^ and assigning each analysed scaffold to the lowest common ancestor of the best hits for each frame. The taxonomic assignments of the NMPF clusters were based on the source scaffolds and are given in Supplementary Data 6. A detailed description of the taxonomic annotation and analysis is given in the Supplementary Methods.

Distribution analysis of the protein clusters across ecosystems and NCBI taxa was performed by creating and visualizing networks with Gephi^57^ using the Yifan Hu algorithm^58^ to generate the layout. As the resulting networks, when taking into account all clusters, were very dense, an association threshold was used to filter the data for better clarity, keeping only the edges where at least 2% of the members of each cluster were assigned to a certain ecosystem or phylogeny. Additional analysis was performed by creating circos plots and distribution matrices, produced using the R/chorddiag^59^ and Processing/P5 libraries, respectively. Bar plots were also created in R, using the R/ggplot2^45^ and R/plotly^60^ libraries. Visualizations of geographical distribution were created using maps from the Natural Earth public domain repository (https://www.naturalearthdata.com/).

### Sequence quality control

The quality of the predicted protein sequences used in the analysis was evaluated by taking into account the predicted gene coordinates and gene density of the source ED scaffolds. In particular, evaluation was performed for scaffolds identified as eukaryotic from the IMG/M pipeline, as well as scaffolds with no taxonomic annotation and low density, as the latter is often indicative of potential eukaryotic contigs, or contigs featuring alternative genetic codes. Furthermore, the distance of each NMPF sequence from its respective scaffold ends was evaluated to detect potentially shortened/truncated genes. Based on the above, a number of metrics have been established to assess the quality of NMPF clusters. Details on the analysis are provided in the Supplementary Methods. The quality assessment of NMPFs is presented in Supplementary Data 7.

### Protein cluster co-occurrence with Pfams

The co-occurrence of NMPFs with known protein domains was determined by performing searches for the existence of Pfam protein domains in the analysed scaffolds containing both novel and known protein-coding genes. The translated sequences of the known genes for each scaffold were searched against the HMM profiles of Pfam using HMMER and the HMM profiles’ default trusted cut-off. All positive hits were assigned to their respective scaffolds and, in turn, to NMPFs containing novel sequences from these scaffolds, as potential co-occurring domains. The co-occurrence frequency percentage of each Pfam domain for each NMPF was calculated, defined as the number of scaffolds containing this domain over the total number of scaffolds associated with the NMPF. The Pfam domains were subsequently mapped to COG^7^ domains and their functional categories. No associations to Pfam were observed for 7,885 NMPFs; for the rest of the clusters, the top five Pfam and COG hits based on their frequency are reported in Supplementary Data 1. Moreover, the gene neighbourhoods of selected NMPFs were visualized as association networks, built with NORMA (v.2.0)^61^, and using COG functional categories to provide annotations.

#### Protein fold prediction

##### Multiple-sequence alignment

The query sequence for the MSA was determined by taking the central or pivot sequence of the seed MSA. Query sequences were defined in each MSA by performing pairwise distance calculations, creating an all-against-all distance matrix, and selecting the sequence with the minimum Hamming distance. The MSAs were then recalculated using the central sequence as a guide, filtering to remove sequences poorly aligned to the query (cut-offs set at 90% for sequence identity and 80% for alignment coverage), as well as poorly aligned positions (low column occupancy). The final MSAs were considered for further analysis if they had more than 16 effective sequences. Calculations were performed using Python and the TensorFlow2^62^, SciKit^63^ and Biopython libraries.

##### TrRosetta for initial screening

For the initial pass, each of the putative protein families was analysed using TrRosetta^25^ to obtain a distogram. Notably, a distogram is a tensor that contains the predicted distance distribution for every pair of residues. By summing the distances of less than 8 Å, a distogram can be converted into a contact map, indicating the probability of contact. The mean of the top probabilities has been shown to be highly correlated with structure accuracy^26^ and can be used to filter for proteins that are probably well structured. This metric is very fast to compute and enables us to quickly scan through 106,198 examples. MSAs with at least 0.5 average probability were selected for AlphaFold prediction, alongside the MSAs with enough effective sequences.

##### AlphaFold for final prediction

As none of the NMPFs match known protein families (Pfam) or structures in the PDB, AlphaFold2^22^ was run with no template input. Five models were generated per run and the model with the best pLDDT average was selected for downstream evaluation.

##### Searching for structural homologues

Before any structural search, regions with low predicted confidence (pLDDT < 0.7) were removed. To test whether there is any structural similarity to experimentally determined structures, a TMalign^64^ search was performed against every domain in SCOPe^27^ (21 October 2021, v.2.0.8), an annotated version of the SCOP^65^ database of protein domains. To test if hits (TM-score > 0.5) were novel assignments (not easily inferred from distant sequence homologues), the TMalign score was also computed for the structure of the top HHsearch hit. Finally, the TM-score could be low owing to the length difference between the target and query. This can happen because the query is multi-domain; to account for this, an additional search was performed against the entire PDB^28^ (accessed on 17 December 2021) using MMalign^66^. To further confirm that any hits to PDB were non-trivial, we compared the predicted structure to the PDB structure of the top hit from an HMM–HMM alignment search, using HHsearch^67^. The predicted structures with non-trivial hits to SCOPe that were supported by HHsearch results are referred to as novel assignments.

##### Clustering

For clustering, the all-versus-all TMalign score was computed for all predicted structures with pTM > 0.7 trimmed to regions with pLDDT > 0.7. Clusters are defined as the connected components of a network, where edges are defined by TM-score > 0.6 for both target-to-query and query-to-target alignment.

### Reporting summary

Further information on research design is available in the Nature Portfolio Reporting Summary linked to this article.

## Online content

Any methods, additional references, Nature Portfolio reporting summaries, source data, extended data, supplementary information, acknowledgements, peer review information; details of author contributions and competing interests; and statements of data and code availability are available at 10.1038/s41586-023-06583-7.

### Supplementary information

Supplementary Information Supplementary Methods, Supplementary Results, Supplementary Figs. 1–13 and Supplementary Tables 1–13.

Reporting Summary

Supplementary Data 1 Top co-occurring Pfam domains for NMPF clusters.

Supplementary Data 2 NMPF structural models and structural/functional assignments, based on searches with TMalign, MMalign and HHsearch against SCOPe and PDB.

Supplementary Data 3 Potential novel structural folds. Sheet 1 displays the list of novel folds, including their statistics (number of effective sequences, TrRosetta and AlphaFold scores) and TMalign/MMalign comparison results to SCOPe and PDB. Sheet 2 shows the top five co-occurring Pfam domains of the NMPFs associated with these folds.

Supplementary Data 4 Contribution of NMPFs in the annotation of metagenomes and example recruitment for future updates. Sheet 1 presents a full summary of all the IMG/M datasets used in this study. Each dataset’s genomic content is presented, including the numbers of previously annotated protein-coding genes (hits to Pfam or to isolate genomes); genes clustered to NMPFs in the present study and remaining unannotated genes. Sheet 2 contains a breakdown of the MCL clustering, displaying which genes were kept in NMPFs and which were removed. Sheets 3 and 4 present the same results, grouped for each ecosystem type. Sheets 5 and 6 present the results of an example NMPF enrichment by recruiting sequences from 360 metagenomic/metatranscriptomic samples per NMPF (sheet 5) and dataset (sheet 6).

Supplementary Data 5 Ecosystem classification in the GOLD, EMPO and ENVO classification schemes. Sheet 1 displays the ecosystem assignments of each ED sample used in the analysis. Sheet 2 displays the top ecosystem assignment of each NMPF cluster in the three schemes. In cases in which an ED sample or NMPF has no assignment to ENVO or EMPO, it was marked as unclassified.

Supplementary Data 6 Taxonomic annotation of NMPF clusters.

Supplementary Data 7 Quality assessment of NMPF clusters.

Supplementary Data 8 IMG datasets

## Data Availability

All of the analysed datasets along with their corresponding sequences are available from the IMG system (http://img.jgi.doe.gov/). A list of the datasets used in this study is provided in Supplementary Data 8. All data from the protein clusters, including sequences, multiple alignments, HMM profiles, 3D structure models, and taxonomic and ecosystem annotation, are available through NMPFamsDB, publicly accessible at www.nmpfamsdb.org. The 3D models are also available at ModelArchive under accession code ma-nmpfamsdb.

## Code availability

Sequence analysis was performed using Tantan (https://gitlab.com/mcfrith/tantan), BLAST (https://blast.ncbi.nlm.nih.gov/Blast.cgi), LAST (https://gitlab.com/mcfrith/last), HMMER (http://hmmer.org/) and HH-suite3 (https://github.com/soedinglab/hh-suite). Clustering was performed using HipMCL (https://bitbucket.org/azadcse/hipmcl/src/master/). Additional taxonomic annotation was performed using Whokaryote (https://github.com/LottePronk/whokaryote), EukRep (https://github.com/patrickwest/EukRep), DeepVirFinder (https://github.com/jessieren/DeepVirFinder) and MMseqs2 (https://github.com/soedinglab/MMseqs2). 3D modelling was performed using AlphaFold2 (https://github.com/deepmind/alphafold) and TrRosetta2 (https://github.com/RosettaCommons/trRosetta2). Structural alignments were performed using TMalign (https://zhanggroup.org/TM-align/) and MMalign (https://zhanggroup.org/MM-align/). All custom scripts used for the generation and analysis of the data are available at Zenodo (10.5281/zenodo.8097349).
