# Supplementary material for: Unraveling the functional dark matter through global metagenomics
Source: Nature. 2023 Oct 11;622(7983):594–602. doi: 10.1038/s41586-023-06583-7 (PMC10584684; doi:10.1038/s41586-023-06583-7)
Supplement: Supplementary file 1 — Supplementary Methods, Supplementary Results, Supplementary Figs. 1–13 and Supplementary Tables 1–13. [file 41586_2023_6583_MOESM1_ESM.docx]

# Supplementary Material

## Supplementary File contents

- **Supplementary File S1 (this file):** Supplementary Methods & Results text, Supplementary Figures S1-S13, Supplementary Tables S1-S13.
- **Supplementary File S2.xlsx:** Top co-occurring Pfam domains for NMPF clusters.
- **Supplementary File S3.xlsx:** NMPF structural models and structural/functional assignments, based on searches with TM-align, MM-align, and HH-search against SCOPe and PDB.
- **Supplementary File S4.xlsx:** Potential novel structural folds. Sheet #1 displays the list of novel folds, including their statistics (number of effective sequences, TrRosetta and AlphaFold scores) and TMalign/MMalign comparison results to SCOPe and PDB. Sheet # 2 shows the Top-5 co-occurring Pfam domains of the NMPFs associated with these folds.
- **Supplementary File S5.xlsx:** Contribution of NMPFs in the annotation of metagenomes and example recruitment for future updates. Sheet #1 presents a full summary of all the IMG/M datasets used in this study. Each dataset's genomic content is presented, including the numbers of previously annotated protein-coding genes (hits to Pfam or to isolate genomes); genes clustered to NMPFs in the present study, and remaining unannotated genes. Sheet #2 contains a breakdown of the MCL clustering, displaying which genes were kept in NMPFs and which were removed. Sheets #3 and #4 present the same results, grouped for each ecosystem type. Sheets #5 and #6 present the results of an example NMPF enrichment by recruiting sequences from 360 metagenomic/metatranscriptomic samples per NMPF (sheet #5) and dataset (sheet #6).
- **Supplementary File S6.xlsx:** Ecosystem classification in the GOLD, EMPO and ENVO classification schemes. Sheet #1 displays the ecosystem assignments of each ED sample used in the analysis. Sheet # 2 displays the top ecosystem assignment of each NMPF cluster in the three schemes. In cases where an ED sample or NMPF has no assignment to ENVO or EMPO, it is marked as “Unclassified”.
- **Supplementary File S7.xlsx:** Taxonomic annotation of NMPF clusters.
- **Supplementary File S8.xlsx:** Quality assessment of NMPF clusters.

## Supplementary Methods

### Sequence collection, filtering, and clustering


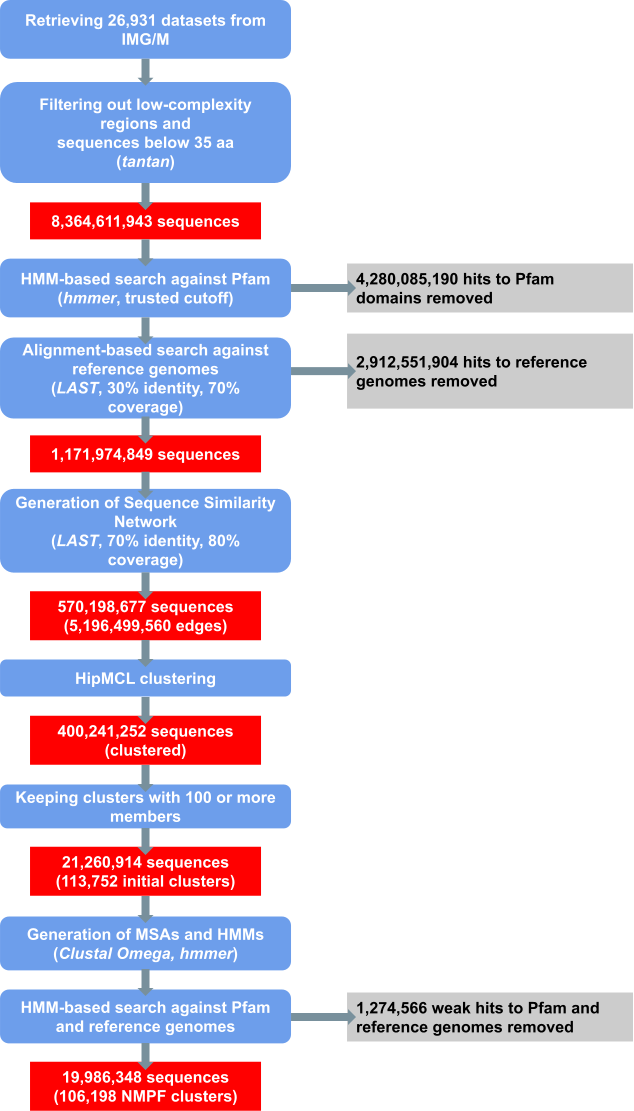


**Fig. M1.** Workflow of the sequence collection, filtering, and clustering procedures. Operational steps are shown in blue. Sequence datasets in each step are shown in red. Sequences that were filtered out are shown in grey.

### Evaluation of the clustering strategy

#### Comparison of clustering algorithms

The main bottleneck in this type of analysis was the clustering part, as most of the state-of-the-art algorithms were unable to cope with this number of sequences (1.32 Billion). To this end, we tried several graph-based and non-graph-based approaches.

In the case of non-graph-based approaches, we tried kClust[^1^](https://www.zotero.org/google-docs/?9j3dit), UCLUST[^2^](https://www.zotero.org/google-docs/?g2IFfm), CD-HIT[^3^](https://www.zotero.org/google-docs/?GZpuMX), and MMSeqs-2.0[^4^](https://www.zotero.org/google-docs/?p1Delh). CD-HIT and UCLUST scale as O(NK), where K is the final number of clusters. In protein sequence clustering K is typically of similar size to N and therefore the total runtime scales almost quadratically with N. Therefore, these options were unsuitable for our analysis.

On the other hand, MMSeqs-2.0 comes with the linclust algorithm, an option to cluster sequences in linear time O(N) but in a heuristic way. MMSeqs-2.0 has been extensively tested in clustering the Metaclust database [^5^](https://www.zotero.org/google-docs/?wAb0LG) consisting of 424 million representative sequences. It has also been used to cluster 1.59 billion sequence fragments at 50% identity with at least 90% alignment coverage and with a maximum E-value of 10e−3 The clustering step found 424 million clusters within 10 h on a server with two 14-core Intel Xeon E5-2680 v4 CPUs (2.4 GHz) and 762 GB RAM.

Despite the great computational efficiency, since linclust is a heuristic and non-exhaustive algorithm, which has hard-coded strict initial filtering to eliminate possible pairwise comparisons, our tests showed that MMSeqs-2.0 tends to create very few and tight clusters leaving the majority of the initial sequences as singletons. This may be due to the requirement that the sequences share at least one identical k-mer substring to be considered for clustering. Another reason is that our analysis was performed before the linclust publication.

Despite the great variety of graph-based clustering algorithms available today, only a few can cope with networks of millions of nodes and edges. For example, SPICi[^6^](https://www.zotero.org/google-docs/?gGHhQ7) is a fast, local clustering algorithm that detects densely connected communities within a network. It is one of the fastest graph-based clustering algorithms with O(VlogV+E) time and O(E) memory asymptotic performance, where V and E are the number of vertices and edges of the network respectively. While SPICi has great performance, it is tailored to analyze dense networks. Louvain[^7^](https://www.zotero.org/google-docs/?9fZNSZ) is a greedy clustering method for identifying communities in large-scale networks and while the exact computational complexity of the method is not known, evidence points to O(VlogV) time performance. Molecular Complex Detection (MCODE)[^8^](https://www.zotero.org/google-docs/?pFLLYA) finds densely connected regions in large protein–protein interaction (PPI) networks with polynomial time complexity O(VEd3), where d is the vertex size of the average vertex neighborhood in the input graph. Restricted neighborhood search clustering (RNSC) uses stochastic local search and tries to achieve an optimal clustering cost by assigning cost functions to the set of clusters of a graph, requiring O(V^2) memory. Affinity-propagation[^9^](https://www.zotero.org/google-docs/?SXY01s) is a clustering algorithm based on the concept of “message passing” between data points and achieves a performance of O(kV^2), where k is the number of iterations.

From all of the aforementioned clustering algorithms, only SPICi was found able to handle our amount of sequences (1.32 billion). However, SPICI is density-based as it tries to detect highly connected regions in a graph and this is the main reason why we did not choose this approach as due to the fragmented nature of metagenomes, our clusters look more like connected components and come with relatively low clustering-coefficient. A star-like cluster for example would be missed by SPICi thus leading to information loss.

Despite the continuous active research in the field and new methods appearing in the literature, the Markov Clustering algorithm (MCL)[^10^](https://www.zotero.org/google-docs/?bjPMFb) has been one of the most successful algorithms. MCL uses random walks to detect clustered structures in graphs with a mathematical bootstrapping procedure and was initially used to detect protein families and protein interaction modules from sequence similarity information. As most of the aforementioned methods (including MCL) struggle to cope with the exponential increase of biological data volumes, a massive parallelization is required.

Recently, we presented HipMCL[^11^](https://www.zotero.org/google-docs/?vPPXFr), a scalable distributed-memory parallel implementation of the MCL algorithm which, in contrast to previous work, takes advantage of the aggregate memory available in all computing nodes. The unprecedented scalability of HipMCL stems from the use of state-of-the-art parallel algorithms for sparse matrix manipulation. HipMCL is written using the MPI and OpenMP programming interfaces, with the principal aim to speed up graph clustering and efficiently detect clusters on a very large scale. Notably, MCL’s core has remained intact, thus making HipMCL the first exhaustive state-of-the-art parallel implementation of the original MCL algorithm. For reference, the HipMCL implementation allowed us to cluster a network of 300 million nodes and ~17 billion edges in only ~6 hours using ~136,000 cores, using infrastructure at the Lawrence Berkeley National Laboratory, USA.

For a direct comparison, we downloaded the Uniref50[^12^](https://www.zotero.org/google-docs/?nOEkE4) database (52 Million sequences) and clustered it with all three algorithms at 50% identity, and 70% alignment coverage. MMSEQ reported 10K clusters consisting of 200K sequences with a size of 10 members or more. SPICi identified 58K clusters consisting of 2M sequences with a density of 0.3 or alternatively 47K clusters with 1.2M proteins for density=0.5. HipMCL generated 491K clusters with a size of 10 members or more consisting of 13.6M sequences (default inflation parameter = 2). For the last two options, the similarity matrix was generated using the LAST alignment (*lastal*) application.

#### Clustering with different identity cutoff values

To investigate the potential effects of using a more relaxed or stringent sequence identity cutoff, clustering benchmarks were performed for a sample sequence set at three different identity levels: 30% (minimum sequence identity for homology modeling)[^13^](https://www.zotero.org/google-docs/?PUFqhs) 70% (current study) and 90% (used by MGnify[^14^](https://www.zotero.org/google-docs/?HU9sPh)). The sample dataset included 2 million sequences (approx. 10%), randomly selected from the total NMPF sequence dataset (19,986,348 sequences). These sequences were grouped into clusters using the same procedure as described in the methods: all-versus-all pairwise alignments were calculated using LAST[^2^](https://www.zotero.org/google-docs/?BpMt8j) with a minimum 80% alignment coverage and were used to prepare sequence similarity networks (SSNs).

The properties of each SSN were analyzed using *igraph*, while clustering was performed with HipMCL. The resulting clusters with 100 or more members were kept and were used to produce multiple sequence alignments (MSAs) and Hidden Markov Models (HMMs). Finally, the clusters were tested on their structure modeling and sequence recruiting capabilities. An overview of the results is given in Table M1.

As shown from the produced SSNs, the 30% and 70% identity thresholds result in clustering the majority of sequences from the dataset (1,996,221 out of 2,000,000). Conversely, applying a very high identity threshold (90%) leads to 431,709 sequences being left out of the network. In addition, the 90% threshold produces a significantly more sparse SSN, as evidenced both by the number of edges (sequence pairs with identity >= the cutoff) and the network’s density (one order smaller than the 30% and 70% SSNs).

Performing clustering with HipMCL also shows significant discrepancies among the three identity thresholds. Generally speaking, small sequence identity cutoff values produce larger clusters and fewer singletons, while higher cutoff values result in significantly more singletons and small clusters. The 30% cutoff produced a total of 159,910 clusters, 3407 of which had 100 or more members. The intermediate, 70% identity threshold produced 185,388 clusters, with 2753 having 100 or more members. The 90% threshold is especially stringent, as it produces only 11 clusters with 100 or more members, containing a total of 1,316 sequences; instead, the majority of the sequences are organized in singletons (1-2 members) and small groups (3-24 members). Focusing on the clusters with 100 or more members, it can be observed that clustering with a 30% sequence identity cutoff results in generally larger clusters (up to 839 members). However, the length of the sequences included in the clusters is more diverse, as the average cluster length ranges from 42 to 449 residues. Conversely, the 90% cutoff produces significantly smaller clusters (up to 298 members), but more consistent in average length (64-298 residues). Finally, the 70% cutoff shows intermediate results, in terms of both cluster size (up to 696 members) and average sequence length (54-254 residues).

The gene recruitment capabilities of the produced clusters were tested by searching their HMMs against two datasets; the complete NMPF sequence dataset in this study, and a sample set of 42,898,470 sequences derived from 100 randomly selected IMG/M datasets. Searches were performed using HMMER (hmmsearch)[^15^](https://www.zotero.org/google-docs/?DvV7ZF), considering hits with a minimum alignment coverage of 70%. The results show that the recruitment capabilities of the 70% and 30% cutoff clusters are comparable, both for the NMPF sequences and for the IMG/M datasets, while the 9% cutoff clusters result in significantly smaller sets of recruited sequences.

**Table M1.** Clustering analysis for an example dataset of 2 million sequences, with sequence identity cutoffs at 30%, 70%, and 90%.

| **Sequence Identity cutoff** | **30%** | **70%** | **90%** | |
| --- | --- | --- | --- | --- |
| ***Sequence Similarity Network properties*** | | | | |
| ***Nodes (total sequences in adjacency matrix)*** | 1,996,221 | 1,996,221 | 1,568,291 | |
| ***Edges (alignment coverage >= 80%)*** | 39,774,424 | 35,123,128 | 10,583,159 | |
| ***Density*** | 1.996e-05 | 1.763e-05 | 8.606e-06 | |
| ***Average Path Length*** | 6.975 | 5.330 | 6.156 | |
| ***Clustering coefficient*** | 0.513 | 0.519 | 0.598 | |
| ***HipMCL clustering results*** | | | | |
| ***Total clusters*** | 159,910 | 185,388 | 298,107 | |
| ***Singletons (1-2 members)*** | 23,674 | 34,529 | 116,534 | |
| ***3 - 24 members*** | 78,842 | 95,803 | 176,497 | |
| ***25 - 49 members*** | 39,168 | 38,726 | 4,717 | |
| ***50 - 74 members*** | 11,030 | 10,290 | 321 | |
| ***75 - 99 members*** | 3,789 | 3,287 | 27 | |
| ***>= 100 members*** | 3,407 | 2,753 | 11 | |
| ***Cluster properties for MCL clusters with 100 members or more*** | | | | |
| ***Sequences in clusters with >=100 members*** | 492,438 | 386,996 | 1,316 | |
| **No. of sequences per cluster (range)** | 100 - 839 | 100 - 696 | 100 - 298 | |
| **Average cluster seq. length (range)** | 42 - 449 | 54 - 254 | 64 - 298 | |
| ***Recruitment Results for MCL clusters with 100 members or more*** | | | | |
| ***Recruitment from 100 metagenomes***  ***(42,898,470 sequences)*** | 77,958 | 60,321 | 119 | |
| ***Recruitment from NMPFs dataset***  ***(19,931,768 sequences)*** | 5,860,529 | 5,072,052 | 47,719 | |
| ***3D model predictions with AlphaFold2*** | | | | |
| **Clusters with enough effective sequences for AlphaFold**  **(Neff > 16)** | 2,590 / 3,407 | 1,926 / 2,753 | 1 / 11 | |
| **Clusters with high-quality AlphaFold 3D models (pTM-score > 0.70)** | 103 / 3,407  (3.02%) | 281 / 1,926  (14.59%) | 0 / 11  (0.00%) | |

Finally, the structure modeling capability of the clusters was estimated by calculating each cluster’s pivot sequence and associated effective sequences, in the same manner as done for NMPFs (see Methods section “Protein fold recognition”). Clusters having the minimum number of effective sequences for AlphaFold[^16^](https://www.zotero.org/google-docs/?N80gMB) (Neff > 16) were considered capable of modeling and were used as input to AlphaFold. The quality of the produced 3D models was then evaluated using the pTM-score, with models having pTM values > 0.70 considered as high confidence.

For the 70% cutoff, 1,926 of 2,753 clusters had enough effective sequences to predict AlphaFold models, 281 of which can be considered as high quality (14.59% of total models). Interestingly, this percentage is in line with the equivalent proportion of high-quality 3D models over the total for the full NMPF dataset in this study (13,096 out of 80,585, or 16.25%). Increasing the threshold to 90% led to only one cluster with enough effective sequences, which produced a low-quality model (pTM = 0.243). Conversely, lowering the identity threshold to 30% increases the number of modeling-capable clusters to 3,407. However, the final number of high-quality predictions was significantly smaller (103 out of 3,407 or 3.02%). This discrepancy is most likely a result of the lower sequence identity in the MSAs of the 30% cutoff, which may rely more on sequence co-evolution patterns rather than known folds in AlphaFold’s training set, but may also predict lower confidence interactions.

### Taxonomy assignment and annotation

#### Taxonomy evidence from IMG/M

The initial taxonomic annotation was performed using the NCBI taxonomy information of the scaffolds contained in each IMG/M dataset, where available. This assignment was based on the analysis of the scaffolds by the original authors, following the DOE JGI metagenome workflow. A detailed description of the pipeline is given by *Clum et al, 2021*[^17^](https://www.zotero.org/google-docs/?PAvhkx)*.* Briefly, the initial taxonomic assignment of each scaffold to the three domains of life (Bacteria, Archaea, Eukaryota) is performed alongside the prediction of its RNA-coding genes, by running tRNAscan[^18^](https://www.zotero.org/google-docs/?cRnoh2) for tRNAs and INFERNAL/cmsearch[^19^](https://www.zotero.org/google-docs/?RiZXak) against Rfam[^20^](https://www.zotero.org/google-docs/?UQyrnl) HMM profiles for the rest of the RNAs and keeping the results with the best score. For protein-coding genes, the pipeline uses multiple predictors (mostly Prodigal[^21^](https://www.zotero.org/google-docs/?dhxQpA) and GeneMark[^22^](https://www.zotero.org/google-docs/?nfSyrv)) and, again, keeps the most reliable prediction. Additional (more detailed) taxonomic assignment can be performed on a per scaffold basis, depending on the existence of hits to known proteins from reference databases. 2,121,004 out of 17,280,119 scaffolds were found to have a taxonomic assignment in IMG/M. However, it should be noted that the majority of the scaffolds used in this study were too short and thus remained taxonomically unclassified. In addition, there is very little information on the taxonomy of viral scaffolds in IMG/M itself.

#### Annotation of viral scaffolds with IMG/VR and DeepVirFinder

For the identification of viral scaffolds, a combination of existing taxonomic assignments from IMG/VR[^23^](https://www.zotero.org/google-docs/?LjO4cd) and machine learning predictions was applied. For scaffolds with lengths > 5 kbs, searches were performed against the IMG/VR database, and all scaffolds identified as viral were retrieved and used. For scaffolds with lengths below 5 kbs, the viral prediction was performed using DeepVirFinder[^24^](https://www.zotero.org/google-docs/?JYQb01) v. 1.0, a reference-free and alignment-free machine learning implementation of the original VirFinder method, designed for the analysis of short contigs. the generated p-values were subsequently converted to q-values using the *R/qvalue* package, in order to obtain estimates of the false-discovery rate. Scaffolds with q ≤ 0.001 were retained as putative viral scaffolds. Overall, the use of IMG-VR and DeepVirFinder produced 577,806 scaffolds identified as viral.

#### Evaluation of eukaryotic scaffolds and alternative-coded metagenomes

The aforementioned steps resulted in 2,698,810 scaffolds with taxonomic annotation, leaving 14,581,381 unclassified scaffolds. These unclassified scaffolds may contain erroneously predicted protein sequences, such as genes encoded with alternative genetic codes, or mistranslated eukaryotic genes. To a large extent, these artifacts are handled both by the gene calling procedures of the IMG/M pipeline and by the sequence length and identity thresholds used in this study. With regard to alternative genetic codes, it should be clarified that the IMG/M pipeline utilizes Prodigal. Prodigal incorporates alternative genetic codes 4 and 25 (Tenericutes and Gracilibacteria) and has models for UGA-reassigned sequences, successfully predicting full-length genes in most UGA-reassigned contigs above 3 kb. As far as eukaryotic genes are concerned, it is important to note that eukaryotic sequences don't assemble well in metagenomes; this means that most unidentified eukaryotic contigs were likely filtered out during sequence collection from IMG/M (minimum scaffold length 500 bps, minimum sequence length 35 aa). Moreover, even though gene finders can erroneously predict genes corresponding to longer exons, due to the variability of intron-exon structure across eukaryotic lineages these longer exons code for protein fragments of variable length, which were eliminated by the alignment coverage cutoff (80%) during clustering.

Despite the aforementioned observations, it is important to investigate whether the inclusion of any falsely-translated sequences may have had an impact on the analysis. In the absence of a taxonomic annotation, one solution was to investigate the gene density per scaffold; Generally speaking, low density can be a signature of eukaryotic contigs, or contigs featuring alternative genetic codes. 3,847,272 out of 17,280,119 scaffolds (22.26%) were found to have a density of 50% or lower. The distribution of these scaffolds among NMPFs is shown in the figure below; the horizontal axis shows the % content from low-density scaffolds in each NMPF, while the vertical axis shows the number of NMPFs (in thousands). As shown, the majority of NMPFs (100,639 of 106,198) have only minor (below 50%) contributions from low-density scaffolds.


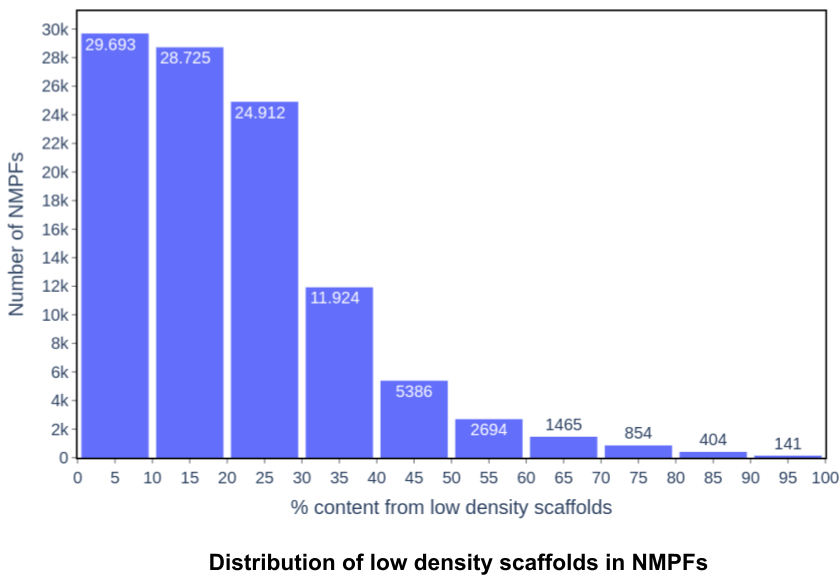


**Fig. M2.** Distribution of genes from low-density scaffolds among NMPFs.

To test for the existence of alternatively coded genes, we re-ran gene calling for the low-density Scaffolds using Prodigal, as in the IMG/M pipeline, and Prodigal-gv, a modified fork designed to improve gene calling for giant viruses and viruses that use alternative genetic codes (<https://github.com/apcamargo/prodigal-gv>). The results of Prodigal-gv were compared to the original results from the IMG/M pipeline (Table M2). 204,788 out of 3,847,272 low-density scaffolds were found to have different genetic codes with prodigal-gv compared to standard prodigal and IMG/M. These encoded 365,052 genes in the original pipeline, 214,408 of which were included in our analysis, distributed over 26,864 NMPFs. With prodigal-gv, 369,548 genes were encoded from these scaffolds. To investigate whether the application of different genetic codes resulted in significant differences in the produced genes included in the NMPF clusters, pairwise sequence alignments were performed between the 214,408 NMPF sequences and the sequences produced by Prodigal-gv. Results showed 316,202 alternative-coded genes have hits to 211,073 NMPF sequences, with an average sequence identity of 84.00 +/- 10.37 % and a minimum alignment coverage of 80%.

**Table M2.** Overview of the differences in gene calling results for low-density scaffolds, using standard models with Prodigal and alternative models with Prodigal-gv.

| **Gen. code change**  **(Prodigal *→* Prodigal-gv)^1^** | **Affected scaffolds** | **Original genes**  **(Prodigal)** | **Original genes included in NMPFs** | **Alternative-coded genes (Prodigal-gv)** |
| --- | --- | --- | --- | --- |
| ***11→4*** | 52,943 | 75,279 | 54,630 | 76,352 |
| ***11→15*** | 47,022 | 94,538 | 50,048 | 92,658 |
| ***4→11*** | 99,734 | 187,430 | 104,444 | 192,667 |
| ***4→15*** | 5,089 | 7,805 | 5,286 | 7,871 |
| ***Total*** | 204,788 | 365,052 | 214,408 | 369,548 |

^1^ Genetic codes are referenced by their number designations by NCBI. Each field in the column references the change of genetic code from standard Prodigal to Prodigal-gv.

Overall, the analysis of low-density scaffolds indicates that they have a minor presence in the majority of NMPFs, with only a few clusters containing more than 50% of genes from these contigs. A small subset (204,788 out of 3,847,272 scaffolds) was found to have different gene products when investigated for the existence of alternative-coded genes; however, comparison of the latter with the original gene calling results showed high (>80%) sequence identity. Taken together, these results suggest that despite the potential existence of alternative-coded genes in the NMPFs, their impacts on the analysis can be considered minor.

Furthermore, to investigate the existence of potential eukaryotic scaffolds, all unclassified datasets were analyzed with two specialized eukaryotic metagenome predictors, Whokaryote[^25^](https://www.zotero.org/google-docs/?Dx1x1C) and EukRep[^26^](https://www.zotero.org/google-docs/?l5qnyv). Whokaryote is a random forest classifier that uses manually selected features based on fundamental differences in gene structure between eukaryotes and prokaryotes, such as intergenic distance, contig gene density, and the existence of ribosome-binding motifs. EukRep is a k-mer-based Support Vector Machine (SVM) classifier trained on binned data, that can be used to annotate metagenomes as eukaryotic. 105,486 scaffolds were predicted as eukaryotic by Whokaryote, while EukRep predicted 289,806 scaffolds, which also included all positive results from Whokaryote. It is important to note that both tools heavily depend on scaffold length, and have been found to underperform for sequences below 5000 bps. As a result, the majority of the analyzed scaffolds remained unclassified by these two methods.

#### Mapping to eukaryotic metagenome-assembled genomes (MAGs)

Searches of the NMPFs was performed against the recently published dataset of eukaryotic metagenome-assembled genomes (MAGs) by *Delmont et al, 2022*[^27^](https://www.zotero.org/google-docs/?tZG0HY). This particular dataset was compiled by analyzing metagenomic datasets from the Tara Oceans project and currently contains more than 700 eukaryotic MAGs, assembled from 280 billion metagenomic reads. More importantly, the dataset’s MAGs contain genes that were predicted through sequence homology against reference genomes and proteomes, metatranscriptomic mapping, and ab initio gene calling. The latter is especially important, as sequences with no homologs to reference sets would be expected to have potential matches to NMPF clusters from our dataset. It should be noted however, that even though the Tara Ocean project is a great example of the assembly of eukaryotic MAGs, it's more of an exception rather than a general trend, because (a)) there were special sample fractionation steps to enrich eukaryotic components, and (b) only a subset of eukaryotic planktonic diversity could be assembled (eukaryotes with large genomes and eukaryotes with high levels of microdiversity couldn't be assembled). Searches were performed using HMM queries with HMMER, applying an alignment coverage cutoff of 70%. A total of 3,950 of NMPFs had hits to sequences from the eukaryotic MAGs, 2,011 of which were primarily eukaryotic (i.e. Eukaryota were the most abundant taxonomy group), while 1,781 were primarily unclassified (> 80% of their sequences had no taxonomic annotation). In contrast, very few hits were detected featuring bacterial, archaeal, or viral NMPFs (83, 11, and 64 hits, respectively). As the 1,781 unclassified families identified in the searches are likely to be eukaryotic, all unclassified scaffolds associated with them were also classified as eukaryotic, leading to the annotation of 269,737 scaffolds.

#### Taxonomy assignment using UniRef50

As a final annotation step, all unclassified scaffolds, including the sequences predicted as eukaryotic by Whokaryote and EukRep, were taxonomically assigned using the taxonomy classification tool implemented in MMseqs2 (*mmseqs taxonomy* version 14-7e284)[^28^](https://www.zotero.org/google-docs/?cFM1Pm) and the UniRef50[^12^](https://www.zotero.org/google-docs/?GKeBog) database. For each scaffold, a six-frame translation was performed and all potential ORFs were searched against UniRef50, with a sensitivity of 2.4 and an E-value cutoff of 10^-6^ (1e-6). Mapping to UniRef50 resulted in taxonomic assignment for a total of 8,323,327/14,581,381 (57.08%) of the previously unclassified scaffolds for bacteria, archaea, eukaryotes, and viruses. Notably, all scaffolds identified as eukaryotic through UniRef50 mapping also included the eukaryotic scaffolds predicted by EukRep and Whokaryote. In addition, an overlap of 9,995 scaffolds was observed between UniRef50-based mapping and the results from mapping to eukaryotic MAGs; for these scaffolds, the UniRef50 results were kept, as they were deeper-level classifications and were deemed more trustworthy. The assignments by UniRef50 were appended to the assignments of IMG/M, IMG/VR, and DeepVirFinder, resulting in a final set of 8,049,154 bacterial 382,761 archaeal, 1,184,393 eukaryotic and 1,406,588 viral scaffolds, leaving 6,257,223 scaffolds with no classification.

#### Taxonomic distribution of NMPFs

The taxonomic distribution of NMPFs was calculated by estimating the percentage distribution of each taxonomic group (Bacteria, Archaea, Eukaryota, Viruses, and Unclassified) in the sequences of each cluster. For each NMPF, the representative taxonomy was also defined in the following manner: if the top taxonomy group was bacterial, archeal, viral, or eukaryotic, that group was chosen as the representative taxonomy. If the majority of the sequences in the NMPF were unclassified, but the second most prominent group had a presence of 20% or more in the family, that group was selected as the representative taxonomy (e.g. for a family with 60% Unclassified, 30% bacterial and 10% archaeal sequences, the representative taxonomy is Bacteria). Finally, if unclassified sequences constituted more than 80% of the NMPF, the family was described as Unclassified.The results are presented in Supplementary File S7.

#### Detection of ribosome binding site motifs

As an additional test, The presence of potential Ribosome Binding Site (RBS) motifs was in the sequences of the scaffolds, and the derived NMPFs were analyzed. RBS motifs are usually found upstream of the starting codon in the 5’-UTR region and are commonly associated with prokaryotic sequences, as well as viral sequences from bacteriophages. RBS detection was based on the prediction of the equivalent feature by Prodigal for all NMPF genes in the scaffolds. Subsequently, the percentage of RBS-containing genes in each cluster was calculated. An NMPF was defined as “RBS-containing” if 20% or more of its sequences came from genes with RBS motifs. A distribution of RBS-containing NMPFs is given in the table below:

**Table M3.** Distribution of RBS-containing NMPF clusters.

| **Top NMPF Taxonomy** | **Total NMPFs** | **RBS-containing (>= 20% of NMPF genes)** |
| --- | --- | --- |
| Bacteria | 59,784 | 40,562 |
| Archaea | 2,843 | 1,851 |
| Eukaryota | 6,471 | 2,077 |
| Viruses | 13,961 | 9,755 |
| Unclassified | 23,139 | 9,575 |

As shown, significant portions of bacterial, archaeal and viral NMPFs have at least 20% of RBS-containing genes. For eukaryotic NMPFs, the number of RBS-containing NMPFs is low (2,077); further examination showed that in these clusters, RBS-containing genes were mostly contributed by scaffolds from other taxonomic groups (bacteria, archaea, etc).

### NMPF enrichment with metagenome sequences

To evaluate how useful can the results of this study can be in further elucidating the “dark matter” of metagenomic protein sequences, we selected a subset of the ED datasets used in the analysis (180 metagenomes and 180 metatranscriptomes covering all environments), and performed test queries on all their sequences, using the HMMs of the NMPFs. Queries were performed using the *hmmsearch* utility from HMMER (inclusion thresholds: 7.0 total, 5.0 domain), applying a 70% alignment coverage cutoff to detect hits. The results are presented in Sheets #5 and #6 of Supplementary File S5 for each NMPF and each ED dataset, respectively. The recruitment of additional sequences through these searches resulted in a total enrichment of ~170% for the number of NMPF sequences (32,365,364 new sequences), in 99,458 out of 106,198 NMPFs. While these results are only representative, they display how much of the currently uncharacterized sequence space can be annotated using the models generated by this study. A full analysis, including sequences from all the ED datasets in IMG/M, will be presented in a future study.

### Sequence quality control

#### Distribution of eukaryotic sequences in NMPFs

972,088 scaffolds are classified as eukaryotic. The sequences of these scaffolds are distributed among 11,551 NMPFs, 8,946 of which contain more than 2% eukaryotic sequences, as shown in Fig. 3. However, the NMPFs in which eukaryotic taxa constitute the primary, representative taxonomy, are only 7,930. More than half of primarily eukaryotic NMPFs (5,256) contain sequences from metatranscriptomes. The transcriptomic evidence essentially validates the eukaryotic sequences in these NMPFs. What is more, most (6,963 out of 8,946) eukaryotic-containing NMPFs are mixed with sequences from other taxonomic groups. This co-occurrence of eukaryotic sequences alongside sequences from other taxa, coupled with the overall quality of their multiple sequence alignments thanks to the threshold used in clustering (70% identity, 80% coverage), is a further indication of their validity.

#### Identification of potentially truncated genes

Identifying complete genes in metagenomic data is a very challenging task [^29,30^](https://www.zotero.org/google-docs/?Zrk49d). Unfortunately, using only genes with valid start and stop codons is not going to eliminate the majority of potential gene fragments. Finding the correct start site is challenging even when annotating complete genomes, and when dealing with fragmented sequences, both gene finders used by the IMG/M pipeline (Prodigal and GeneMark) may often pick incorrect start sites. Therefore all genes that don't have another gene between their start/stop and the edge of the contig are suspect and may be gene fragments.

With the aforementioned caveat in mind, one potential way of tracking possibly truncated genes is by considering the distance of their start/end position from the scaffold ends. Generally speaking, genes located in the center of a contig have a higher probability of being complete, while genes located close to the ends could potentially be truncated. A distribution of complete genes vs potentially truncated genes (i.e. near contig ends) is presented in the figure below (Fig. M3). As shown, approximately 10.4 million sequences in our dataset can be classified as “possibly truncated”, as they were located within 10 bases from one or both ends of their scaffold (Fig. M3-a). However, this can be attributed to the features of the scaffolds themselves; as 16,457,299 of the scaffolds are very short (length < 5 kbs); at the same time, 10,378,119 of these scaffolds contain only 1 or 2 genes in total; thus increasing the possibility of their genes being near the ends, and, therefore, possibly truncated. With regards to the families themselves, 28,801 NMPFs have more than 90% of their sequences near contig ends (Fig. M3-b). However, they follow the same trends as the rest of the NMPFs concerning their length, as shown by an inspection of the clusters’ length for both groups (Fig. M3-c). As a final evaluation of the effects that potentially truncated genes may have had on the properties of the clusters, we evaluated them by running searches against a set of 360 metagenomes and compared the length of the clusters against the length of their sequence hits. As shown by the scatter plots in Fig. M3-d and M3-e, the distribution of the sequence hit lengths is very similar for NMPFs with >90 % and < 90% genes near the scaffold genes. Taken together, these results indicate that the increased presence of genes near contig ends for these NMPFs does not alter their properties drastically, compared to the rest of the dataset.


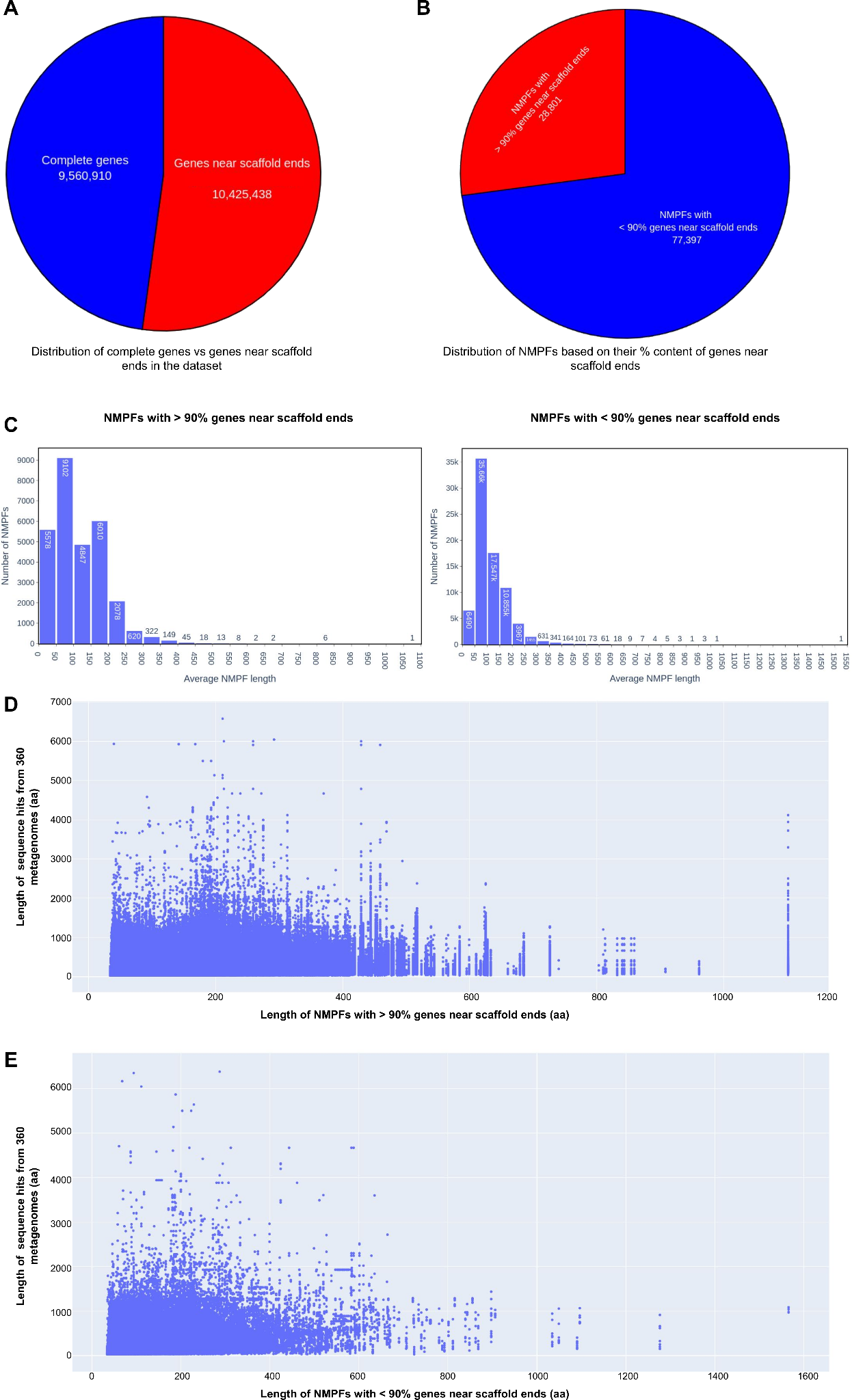


**Fig. M3.** Evaluation of the contributions from genes near scaffold ends to the analysis. A) Distribution of genes in the dataset based on their distance from the scaffold ends. B) Distribution of genes near scaffold ends in the NMPF clusters. Two groups are shown; NMPFs with >90% genes near ends and with < 90%. C) Distribution of average cluster length for NMPFs with >90% (left) and < 90% (right) genes near scaffold ends. D-E) Cluster lengths vs sequence hit lengths for the enrichment trials of NMPFs against 360 metagenomes, for NMPFs with > 90% genes near scaffold ends (D) and <90% near scaffold ends (E).

Notably, out of 28,801 NMPFs with more than 90% genes near scaffold ends, 20,696 clusters are represented in metatranscriptomes, indicating that they contain actively expressed genes. At the same time, 10,249 of these clusters contain sequences from genes with valid RBS motifs (notably, 3,029 of these NMPFs are metagenome-only families), meaning that they very likely are not truncated, at least as far as the start codon (and N-terminus for proteins) is concerned. Finally, 23,235 out of 28,801 “potentially truncated” NMPFs have a 3D structure available, indicating that the source sequences are capable of at least folding into a structural domain.

#### Investigation of NMPF sequence length with respect to scaffold length

The majority of the scaffolds (~16.5 out of ~17.28 million) in this study are short (< 5,000 bps); in fact, 10,210,177 scaffolds have sequence lengths between 500 and 1,000 bps. Despite their small length, a significant number of these scaffolds actually contain multiple genes (2 or more), either exclusively NMPF sequences or NMPF sequences coupled with other genes. A distribution of the scaffolds used in the study with respect to their length and number of genes, is given in the table below:

**Table M4.** Distribution of scaffolds based on scaffold length and number of contained ORFs.

| **Scaffold Length range (bps)** | **Total Scaffolds** | **Single gene scaffolds** | **Multi-gene scaffolds** |
| --- | --- | --- | --- |
| 500 - 1,000 | 10,210,177 | 3,401,543 | 6,808,634 |
| 1,000 - 2,000 | 4,267,821 | 282,593 | 3,985,228 |
| 2,000 - 5,000 | 1,979,301 | 15,297 | 1,964,004 |
| 5,000 - 7,000 | 288,097 | 351 | 287,746 |
| 7,000 - 10,000 | 191,687 | 183 | 191,504 |
| > 10,000 | 343,036 | 203 | 342,833 |

A distribution of gene lengths, both for NMPF and for non-NMPF sequences in the analyzed scaffolds is shown in the bar chart below:


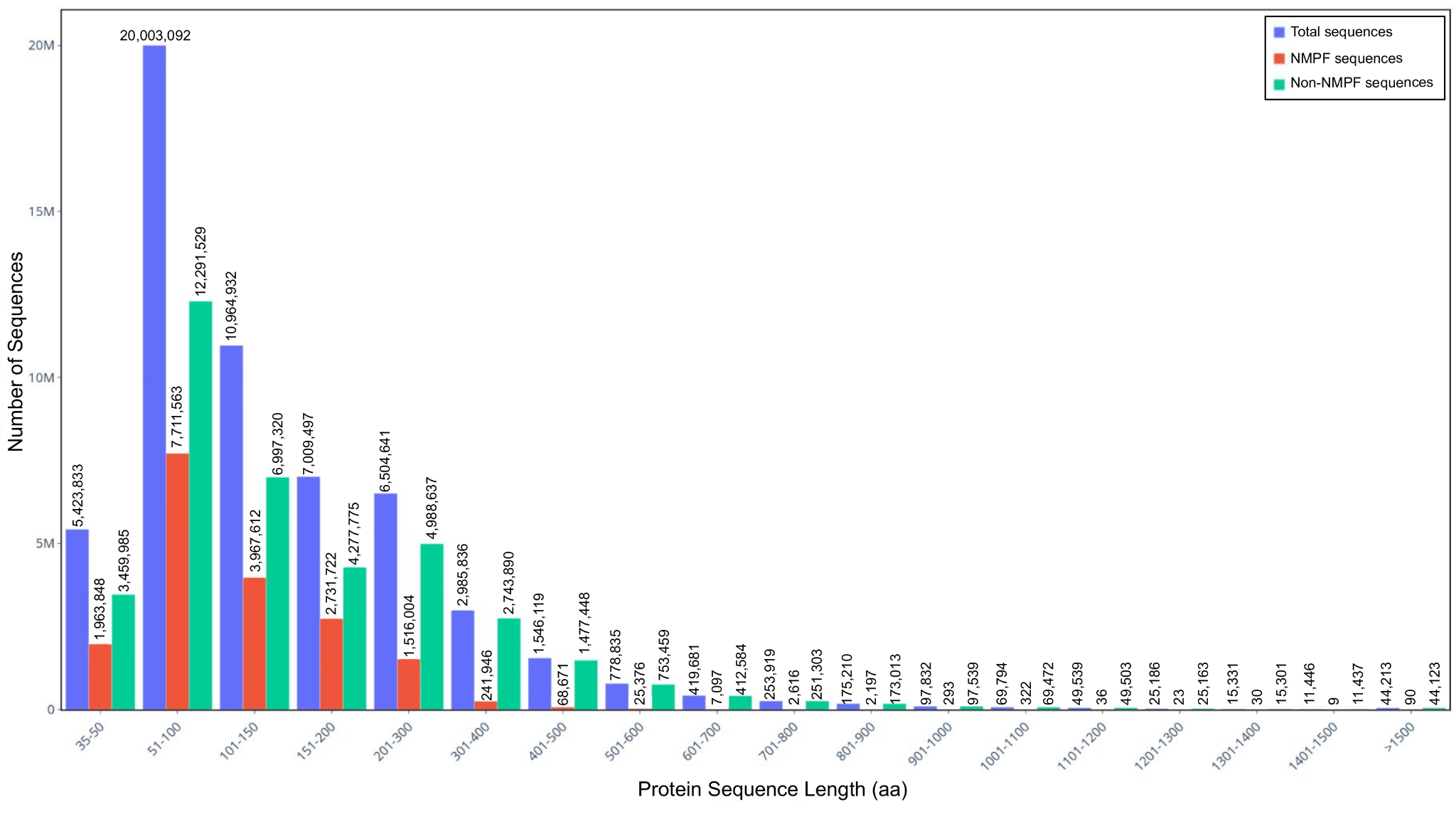


**Fig. M4.** Bar chart distribution of protein sequence length vs. number of sequences for all scaffolds. The horizontal axis shows the sequence length in amino acids, while the vertical axis shows the number of sequences in each length range. Measurements are given for all scaffold sequences (blue), NMPF sequences (red), and non-NMPF sequences (green).

The bar chart shows the distribution of sequence length vs number of sequences for all sequences in the scaffolds (blue), NMPF sequences (red), and non-NMPF sequences (green), i.e. sequences with hits to Pfam or reference genomes, as well as any sequence not clustered by HipMCL during the creation of the NMPF clusters. As shown, the majority of protein sequences in the scaffolds of our dataset tend to be short in length. A number of longer sequences also exist, but they are significantly fewer compared to short sequences. Breaking down the aforementioned length distribution, by also considering the source scaffolds’ sequence length, further reveals the importance of scaffold length, as shown in the following charts:


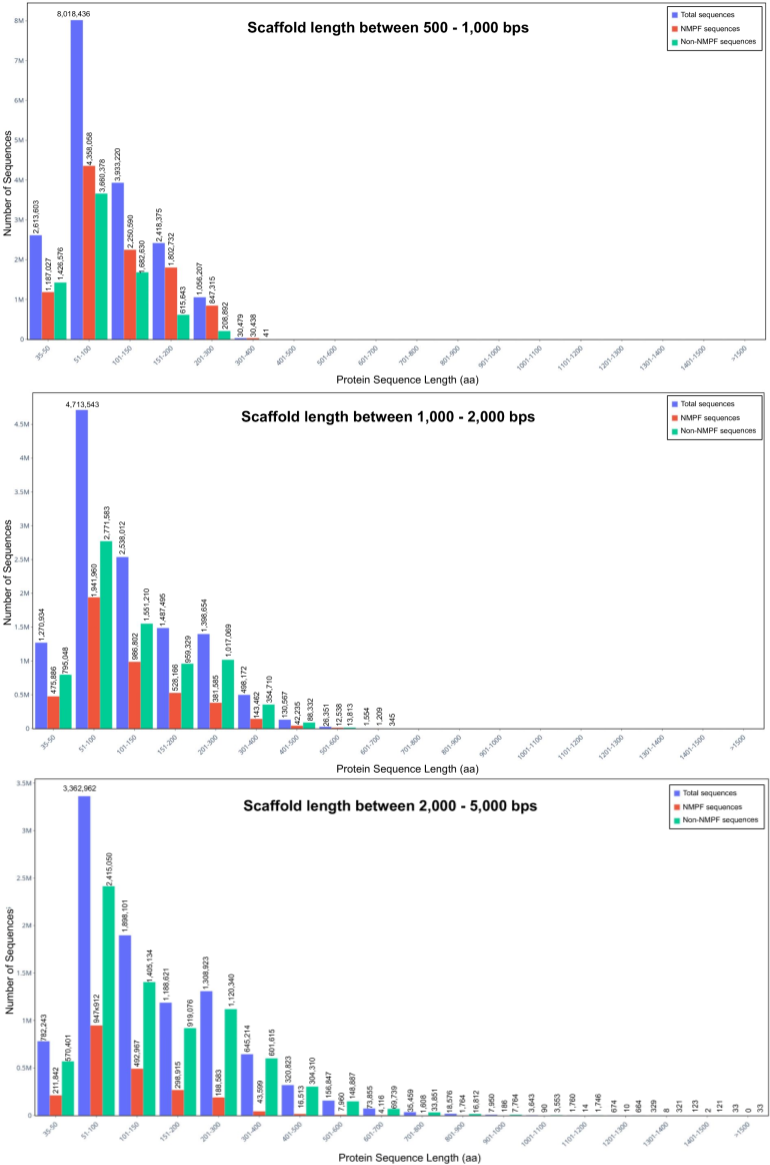


**Fig. M5.** Bar chart distribution of protein sequence length vs. number of sequences for short (below 5000 bps) scaffolds. Representations follow the same rules as in Fig. M4.


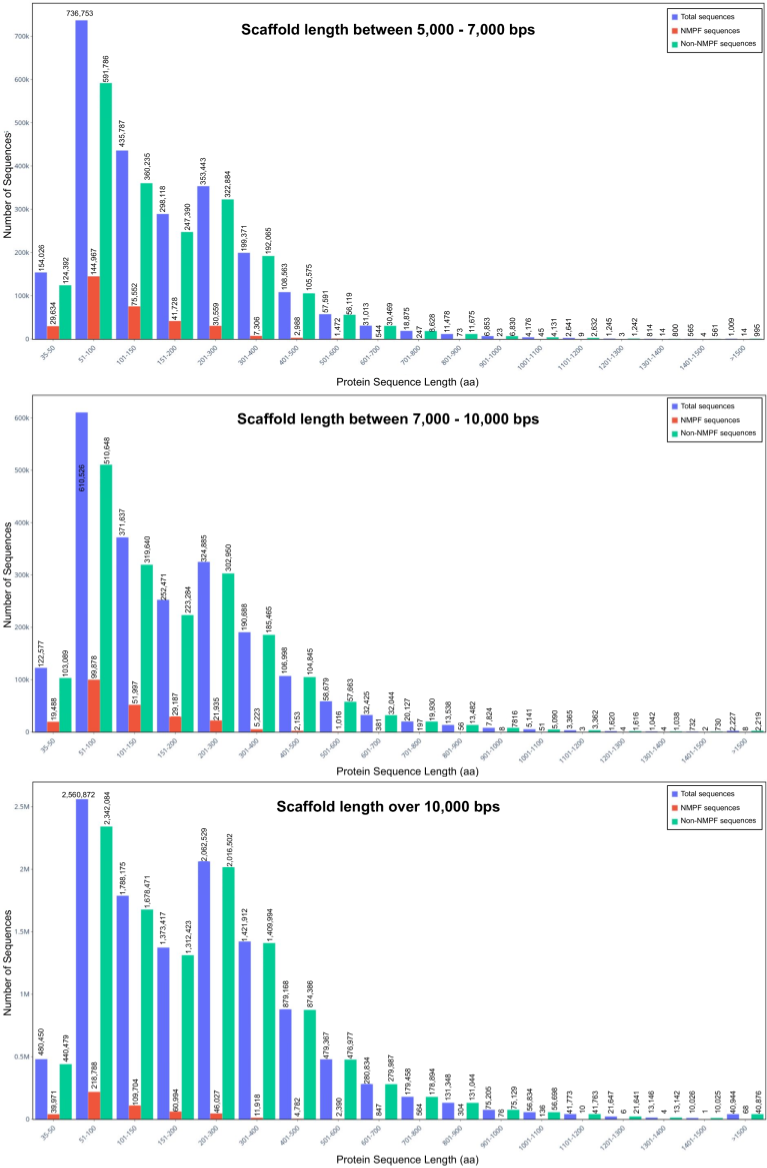


**Fig. M6.** Bar chart distribution of protein sequence length vs. number of sequences for longer (over 5000 bps) scaffolds. Representations follow the same rules as in Fig. M5.

**Table M5.** Distribution of short genes in different scaffold length categories.

| **Scaffold Length range (bps)** | **Total Genes** | **Short genes (35-100 residues)** |
| --- | --- | --- |
| 500 - 1,000 | 18,950,993 | 11,511,981 (60.75%) |
| 1,000 - 2,000 | 12,517,690 | 6,436,125 (51.42%) |
| 2,000 - 5,000 | 10,105,604 | 4,443,978 (43.98%) |
| 5,000 - 7,000 | 2,477,553 | 954,585 (38.53%) |
| 7,000 - 10,000 | 2,179,960 | 786,435 (36.08%) |
| > 10,000 | 12,155,745 | 3,299,565 (27.14%) |

As it can be observed, short scaffolds (in the 500-1000, 1000-2000 and 2000-5000 bps ranges) contain almost exclusively short protein sequences, while longer sequences mostly appear in longer scaffolds. Considering that the latter form the minority of the analyzed scaffold set, it is not surprising that longer sequences are also under-represented. Still, one can still observe that even in large scaffolds, short genes (both NMPF and non-NMPF) constitute the majority of the ORFs, again, demonstrating that short gene length is characteristic for metagenomic sequence data.

The distributions presented above also reveal the importance of another factor, namely, the strategy for sequence selection and clustering. As shown, a lot of the longer sequences in the scaffolds are not part of the NMPF clusters. These non-NMPF sequences include hits to Pfam domains or genes from reference genomes, which were discarded, as well as sequences that were left out during clustering, either as singletons or as parts of clusters with less than 100 members.

#### Establishment of NMPF quality metrics

Based on the analyzed features of the NMPF clusters, a number of quality metrics have been established, to assess and evaluate the quality of each NMPF based on its features. These metrics are presented in Supplementary File S8 and include the following:

1. Metatranscriptome representation

2. Top NMPF taxonomy

3. Percentage of NMPF genes with valid RBS motifs

4. Percentage of NMPF genes near scaffold ends

5. 3D model prediction with AlphaFold2 (and its associated pTM score) and

6. Number of source GOLD sequencing projects.

The combined consideration of these metrics can help evaluate the overall quality of each NMPF cluster.

### Multiple Sequence Alignments

Python script used to generate seed Multiple Sequence Alignments (MSA) and consensus sequences. The script accepts a single-column list of FASTA-formatted MSA files as input and produces FASTA-formatted files for the seed MSA and consensus sequence of each input alignment.

| #!/usr/bin/env python3  # generate_seed.py  # run as:  # python3 generate_seed.py list_of_fastas  # where ‘list_of_fastas’ contains the names of the input MSAs in this format:  # F105000.fasta  # F045821.fasta  # F000388.fasta  from sys import argv,stderr  import multiprocessing as mp  from Bio import AlignIO  from Bio.Align import AlignInfo  from prody import *  #global variables to be changed by the user  SEQ_ID=0.9 #sequence identity cutoff, 90%  COV=0.75 # alignment coverage, 75%  CPUS=16 # number of CPUs to use for multiprocessing  def create_seed_and_consensus(aln_file):  #parse the input MSA  msa=parseMSA(aln_file, format="FASTA")  name=aln_file.rstrip(".fasta")  #produce seed alignment based on SEQ_ID and COV  seed_msa=refineMSA(msa, seqid=SEQ_ID, rowocc=COV)  #if for some reason, the applied COV cut-off removes ALL sequences,  #recalculate the alignment using only the SEQID and warn the user  if seed_msa.numSequences()==0:  print("Applying alignment coverage %s resulted in empty alignment, using only the seq-id cutoff instead..." %COV, file=stderr)  seed_msa=refineMSA(msa, seqid=SEQ_ID)  writeMSA("%s_seed.fasta" %name, seed_msa)  #parse the seed MSA as an AlignIO object  alignment=AlignIO.read("%s_seed.fasta" %name,"fasta")  #calculate the alignment summary  summary=AlignInfo.SummaryInfo(alignment)  #create a consensus sequence  cons=summary.dumb_consensus()  #write the consensus in a fasta file  cons_out=open("%s_consensus.fasta" %name,"w")  cons_out.write(">%s_consensus\n%s\n"%(name,cons))  cons_out.close()  print(">%s_consensus\n%s"%(name,cons), file=stderr)  #code to run the create_seed_and_consensus method in parallel  pool = mp.Pool(CPUS)  jobs = []  f=open(argv[1],"r")  files=f.readlines()  f.close()  dataset=[i.rstrip() for i in files]  run=pool.map(create_seed_and_consensus, dataset) |
| --- |

## Supplementary Results

### Sequence collection & clustering

In total, we identified 106,198 families with ≥100 members that will be referred to as Novel Metagenome Protein Families (NMPFs) (Table 1; last column). For comparison, we identified 92,909 protein clusters in the corresponding set of protein clusters with ≥100 members from reference genomes. By directly comparing the two clustered sets (reference vs ED protein clusters), we observed an increase of the ED protein clusters by >14-fold for clusters with ≥3 members, >3-fold for clusters with ≥25 members, a ~2-fold increase for clusters with ≥50 and ≥75 members as well as an increase for clusters with ≥100 members (Table 1).

While the metagenome sequence space is intrinsically more fragmented than that of the reference genomes, and by effect, a higher percentage of genes would be erroneous or incomplete (which is also one of the reasons we decided to focus further analysis on the larger clusters), these results also suggest that much of protein sequence space remains to be explored. This is also supported by rarefaction curves generated from the ≥100 member clusters (Figure 1b). These curves show that as more samples became available, the cluster number increased linearly for reference genomes and exponentially, without reaching a plateau, for metagenomes.

Further comparison of clustered proteins revealed additional differences between clusters from the ED and reference genome datasets (Figure 1c-d & Suppl. Figure S1). The majority of ED clusters consisted of a relatively small number of sequences (100-150 per cluster for approx. 54% of all clusters) (Figure 1c), found on an equally small number of scaffolds (100-150) and distributed across similarly small numbers of samples (50-100) . By contrast, reference genome clusters were typically associated with a larger number of genome samples (100-300). Another difference was observed in the sequence length of the cluster members; the majority of ED clusters contained shorter sequences/fragments (less than 100 residues), whereas the sequence lengths of the reference genome clusters ranged from 100 to 500 residues for most clusters. This discrepancy in sequence length can be attributed to the length of the source scaffolds. Most of the ED clusters were composed of proteins coming from scaffolds of various lengths below 5 kbs. This, in turn, leads to shorter protein sequences compared to the equivalent results from reference genomes.

To evaluate the extent of the currently unexplored functional dark matter space that can be annotated by the profiles of these new clusters, we selected a subset of the ED datasets used in the analysis (180 metagenomes and 180 metatranscriptomes covering all environments), and performed test queries on their sequences, using the HMMs of the NMPFs (see Supplementary Methods). The recruitment of additional sequences through these searches resulted in a total enrichment of more than 200x (32,365,364 new sequences over the 18,890 NMPF genes originally contained in these datasets), for 99,458 out of 106,198 NMPFs (Supplementary File S5). While these results are only representative, they clearly demonstrate that a significantly larger uncharacterized sequence space can be annotated using the models generated by this study. A full-scale enrichment across all the ED datasets in IMG/M, will be presented in future studies.

### Habitat distribution

Each habitat was further divided into subcategories. The corresponding distributions are depicted as Circos plots in Fig. S2. In freshwater and marine environments, protein clusters are, in general, uniformly distributed among the various subtypes (e.g. lake, river, etc.), with the majority of the clusters being shared among the environments. In soil, a significant proportion of the clusters are unclassified, meaning that they are widely distributed in a large array of environments. In plants, the majority of the clusters belong in the closely related rhizosphere and rhizoplane environments. Zooming in on the human subcategories, we can see that the vast majority of clusters are classified into the large intestine, with the oral cavity being the second largest subcategory. This, however, can be attributed to the nature of the human metagenome samples, which were mostly collected from the gut microbiome. In mammals, a significant subset of clusters come from livestock species (sheep, bovine, etc.), while most clusters remain unclassified. In the other host-associated categories, almost half of the clusters are grouped into Arthropoda and Annelida. Finally, the engineered habitat clusters follow a distribution pattern similar to the freshwater and marine environments.

### Metadata distribution

3,872,485 (22.41%) of the scaffolds contained only novel protein genes (novel-specific scaffolds). Furthermore, 3,700,126 of these were single-gene scaffolds (containing only one gene/protein). The number of proteins in the rest of the novel-specific scaffolds ranged from 2 to 9, with most (164,133) scaffolds containing 2 genes, and only one scaffold containing 9. As they are composed of exclusively novel protein genes, they are unclassified with regard to their phylogeny. These scaffolds are mostly metagenomic, with very few metatranscriptomes, and are primarily associated with environmental habitats (freshwater, marine, and soil) and, to a lesser extent, host-associated categories like human, mammals, or plants (Supplementary Tables S7 & S8).

### Structure Distribution

We have run AlphaFold2 on NMPFs with at least 16 diverse sequences, or where TrRosetta predicted a well-structured protein (see Methods). The results are summarized in Figure 4a. Of the 81,345 NMPFs that met the above criteria, 80,585 3D models were predicted, with 13,096 NMPFs having a high confidence (pTM-score>0.700) prediction. The predicted TM-score integrates both the predicted confidence per position and the predicted alignment error (pAE) for every pair of positions, indicating the confidence of domain-domain orientations.

Based on structural clustering, these high-confidence predictions represented 4,361 unique structures. To explore the novelty or functions of these structures, we compared them to experimentally determined structures from SCOPe and assemblies from the Protein Data Bank (PDB). 3,808 structures (12,253 NMPFs) had a significant structural overlap with at least one SCOPe domain (TMscore>0.5). Of these, 2,718 (7,769 NMPFs) had a non-trivial hit, indicating that 62.3 % of high-quality predictions had some similarity to at least one SCOPe domain or PDB assembly.

These novel assignments, based on structural similarity, can now be used for functional prediction of the corresponding sequences. A few examples are shown in Figure 4c. For example, family F034396 had no hits to the PDB using HHsearch (top hit of e-value:12), yet a strong hit to the PDB using a structural search of the SCOPe domain d3cmba1 (TMscore:0.69), with the function of acetoacetate decarboxylase. Other examples with no HHsearch hits (e-value>10), yet strong structural hits included: F010804-d1z45a1 (TMscore:0.73, galactose mutarotase), F097565-d1xkra_ (TMscore:0.73, chemotaxis) and F001800-d3ejva1 (TMscore:0.62, NTF2). We stress that these cases should be treated as informed predictions that require experimental validation since the same fold does not always correspond to the same function. For a full list, see Supplementary File S3. However, some validation and additional functional annotation can be performed by combining these novel assignments with other NMPF metadata, such as gene co-occurrence. A few examples are given in Supplementary Figure S18. For instance, family F025334 adopts a 7-bladed β-propeller fold similar to the Sema domain (d62vzm1); such domains are typically observed in the peripheral membrane or lipid-anchored proteins and are used to bind to the lipid bilayer. An investigation of F025334’s gene neighborhood, presented as an association network and annotated using COG functional categories, shows that the NMPF is co-expressed with Pfam domains associated with cell wall/membrane/envelope biogenesis; this indicates that F025334 may participate in these processes by binding to the cell membrane. Similarly, family F000037 is structurally homologous to the SH2 domain, a fold associated with kinase-related pathways and the recognition of phosphorylated Tyr residues. The family’s gene neighborhood consists of Pfam domains implicated in processes regulated by kinases (DNA replication, recombination, and repair or energy production), in agreement with the function inferred from structural homology.

To confirm that the remaining 553 proteins with no SCOPe hit were novel folds, a more thorough search was performed against all PDB biological assemblies (biounits), including all possible chain permutations, using MMalign30. 345 had a hit to at least one PDB entry, of which 305 represented additional novel assignments. The remaining 208 were subjected to further filtering, removing predictions where 50% of the structure matched a SCOPe domain. Finally, 162 folds and/or domain-domain orientations from 223 NMPFs, were identified as novel (Figure 4b). A complete list of these folds is provided in Supplementary File S4.

While the absence of any significant structural homology precludes the reliable functional annotation for these novel folds, some hints towards their potential function can be gleaned from their associated metadata. Characteristic examples are given in Supplementary Figure S19, showcasing the gene neighborhood and ecosystem metadata of three NMPFs with novel structural folds. Family F001278 is primarily found in Soil ecosystems and co-occurs with two domains associated with post-translational modifications and protein turnover (Peptidase M10 and Fe-S assembly), indicating that this fold may participate in protein translation, perhaps as a part of a chaperone. On the other hand, F006270 is primarily found in plant (and, to a lesser extent, soil) ecosystems; its gene neighborhood contains domains associated with functions typically associated with membranes, such as defense mechanisms or substrate transport, indicating that this fold may attach to membranes. Finally, F041847 co-occurs with domains associated with defense mechanisms and cell wall/membrane/envelope biogenesis, indicating that the fold may also participate in these functions.

### Limitations of the study

There are a number of limitations underlying the metagenomic data and methodology used in this study. One limiting factor to consider is the short size (below 5 kbs) of the majority of scaffolds used in this study. This inadvertently introduces a bias in the gene-calling procedure toward shorter genes, compared to the equivalent sequences from reference genomes. At the same time, it leads to ambivalence with regard as to whether genes retrieved from these scaffolds can be considered as complete or truncated, as their end positions are often close to the contig ends. However, it should be noted that due to the required alignment coverage of at least 80%, potentially truncated sequences have to be sufficiently complete in order to cluster with full-length sequences (defined as located in the middle of longer scaffolds). This requirement has largely precluded the enrichment of NMPFs with fragmented proteins. However, even in the case of NMPFs with a high percentage of these suspect sequences, the clusters are found to produce stable 3D models (often with high structural quality, as evidenced by pLDDT and pTM scores), many of which have structural homologs to SCOPe domains. As a result, families containing such sequences could potentially represent protein fragments or protein domains that form parts of multi-domain sequences, or components in multimeric complexes.

An additional potential limitation may be the inclusion of eukaryotic sequences in the sequence dataset, which may introduce errors in the analysis. Yet, as shown (Taxonomic Distribution, see also Supplementary Methods), the contributions from eukaryotic scaffolds are relatively minor, and the majority of the associated NMPFs also contain data from metatranscriptomes and/or prokaryotic taxa in sequence alignments, supporting their validity. However, until reliable eukaryotic gene predictors for metagenomes become available, eukaryotic, as well as unclassified NMPFs and sequences should be handled with care.

Finally, one important issue may stem from the choice of using a strict sequence identity threshold during clustering (70%), to increase the confidence that all proteins within each cluster have the same function. This resulted in a large number of clusters with a small number of closely related sequences. For structure prediction, this threshold may be too strict, since aligned proteins with as low as 30% identity often share the same structure, even if their functions differ. This may have inadvertently biased our most confident structure predictions towards known folds in AlphaFold's training set, resulting in a seemingly small number of novel folds. By lowering the threshold, we would expect the clusters to increase in size and sequence diversity, allowing AlphaFold to rely more on the coevolution patterns in the multiple sequence alignments, as opposed to fold recognition for structure prediction. However, lowering the threshold may also introduce artifacts from the inclusion of spurious proteins or pseudogenes, leading to lower-quality sequence alignments and, therefore, low-quality 3D models. An analysis of the effects of changing the sequence identity threshold is presented in Supplementary Methods, conducted for a sample subset of the NMPF sequences. Lowering the identity threshold to 30% during clustering produces more clusters with sufficient sequence diversity; however, the number of high-quality AlphaFold predictions drops as compared to 70% cutoff. This observation indicates that despite the increase in the total number of clusters amenable to modeling, an equivalent increase in high-quality AlphaFold predictions is not guaranteed. A more comprehensive investigation of the effects of clustering parameters in 3D structure modeling will be explored in the future.

### Supplementary Figures


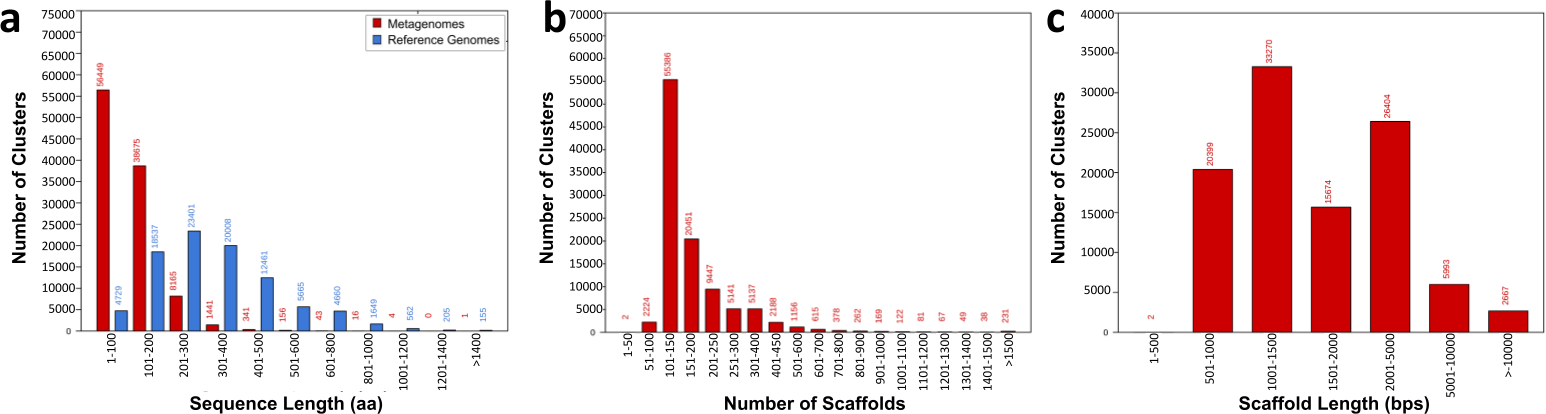


**Fig. S1.** a) Number of clusters vs sequence length for metagenomes/NMPFs (red) and reference genomes (blue). b) Number of NMPF clusters vs the number of source scaffolds. c) Number of NMPF clusters vs scaffold length.


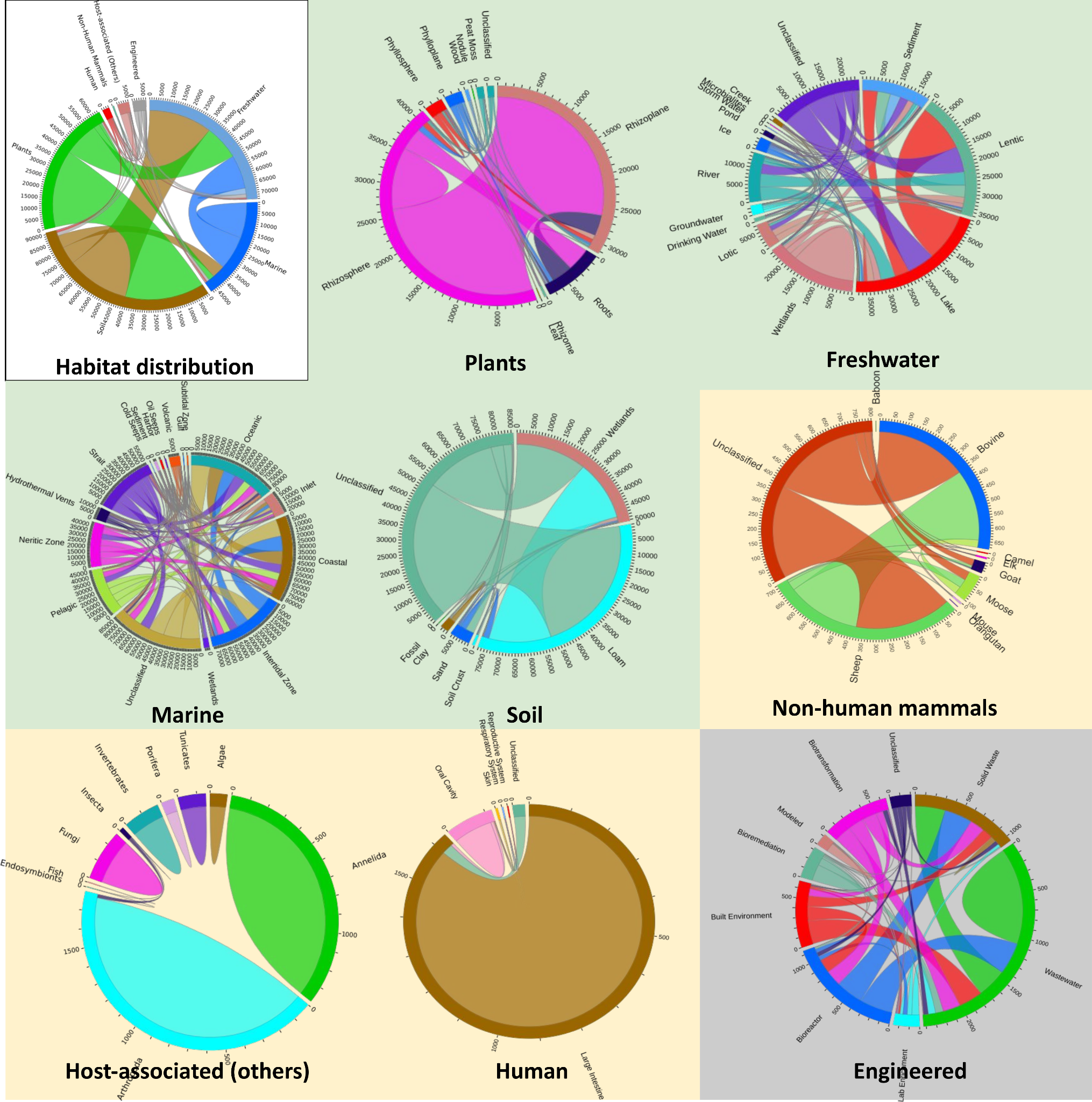


**Fig. S2.** Circos plots of the protein clusters overlapping across the subcategories of each main ecosystem type.


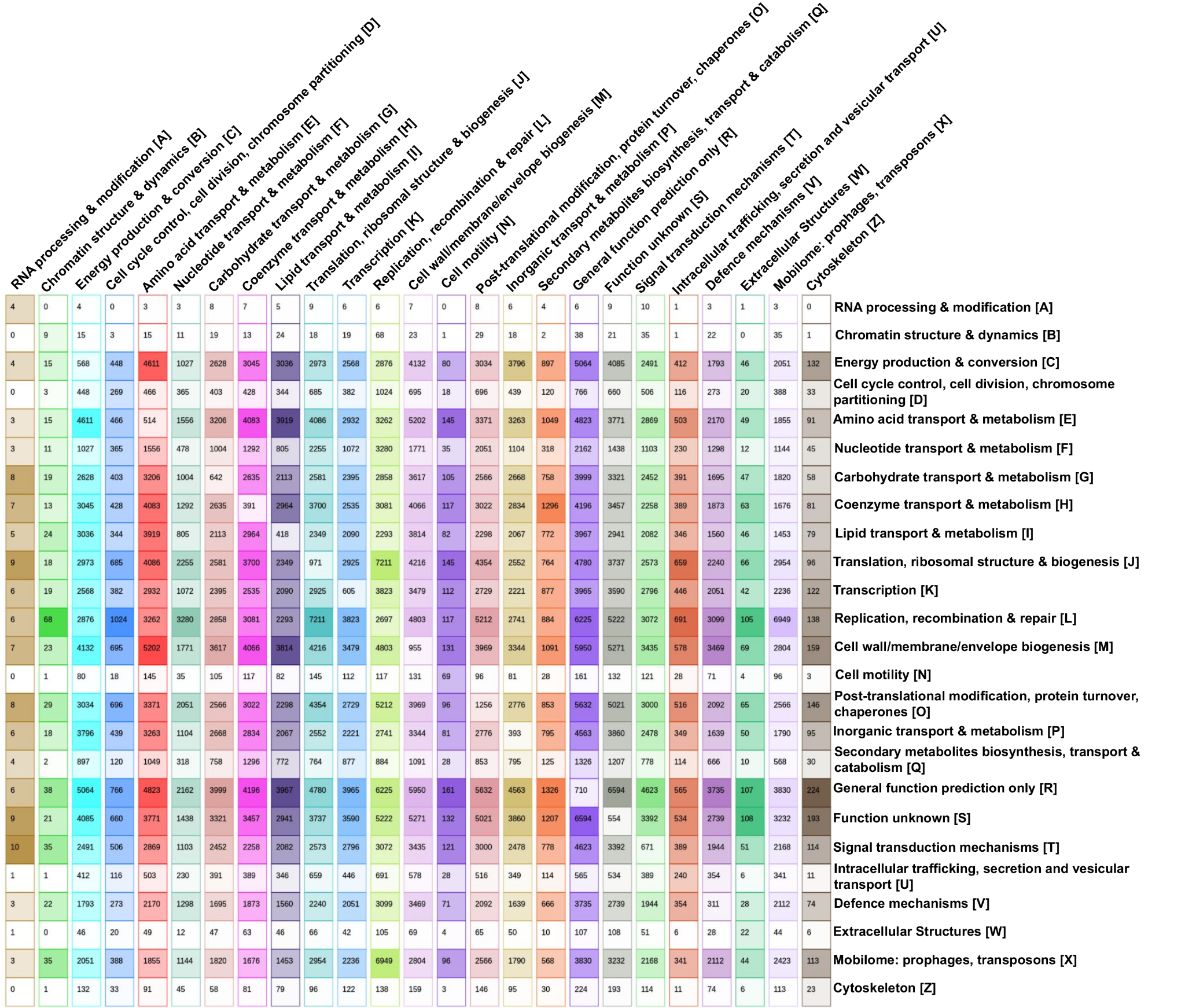


**Fig. S3.** Distribution of NMPF clusters across COG functional categories, as inferred from gene neighborhood analysis, in a 25x25 matrix. Each cell in the matrix presents the common NMPFs in a binary combination of two categories. The diagonal of the matrix displays the category-specific NMPFs.


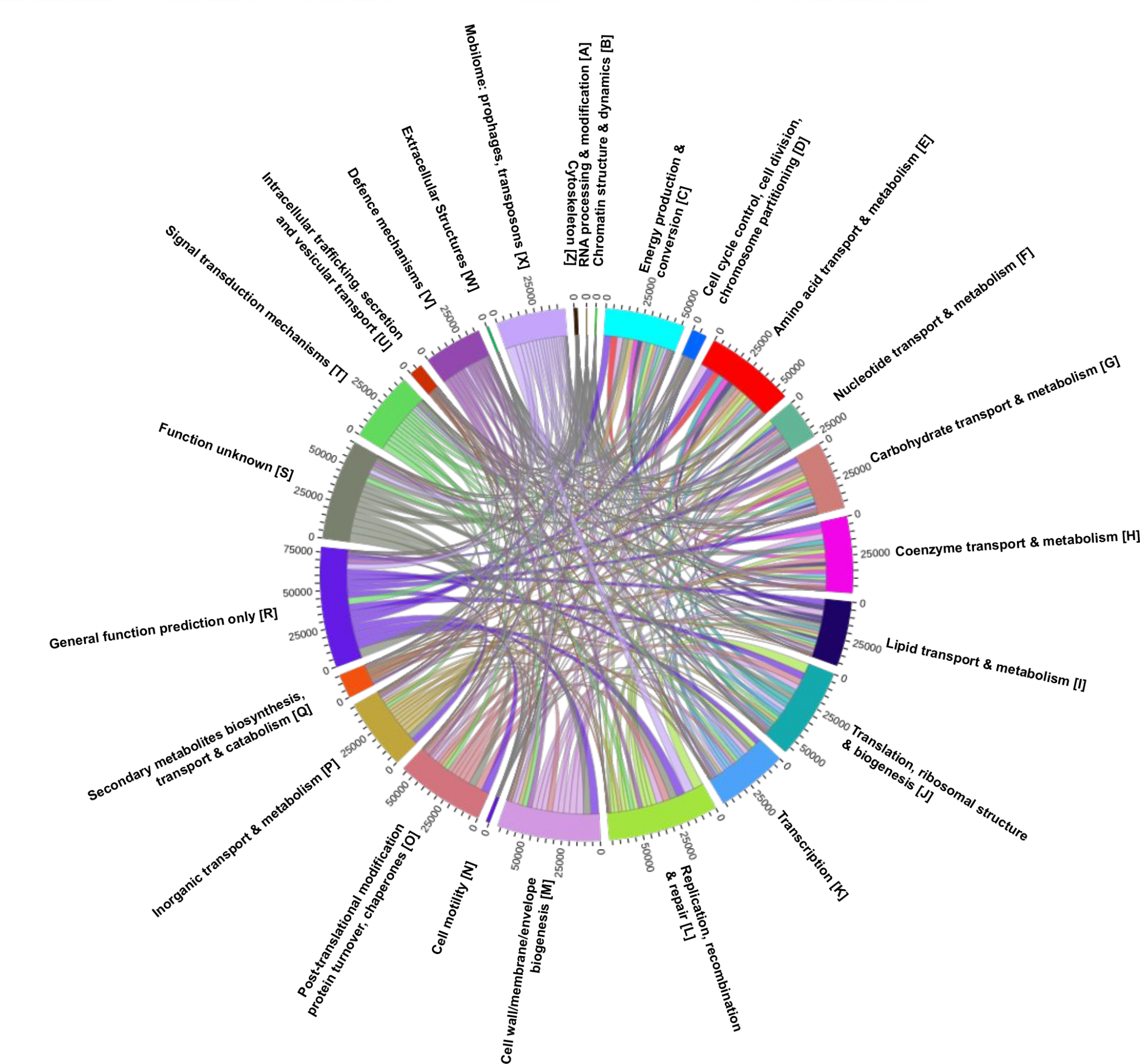


**Fig. S4.** Distribution of NMPF clusters across COG functional categories, as inferred from gene neighborhood analysis, in a circos plot. The distribution of the categories is presented in a chord-like circular diagram. The rim of the diagram represents the total size of the categories (i.e. number of NMPFs), with the numbers outside the rim indicating the size scale. The intersections of categories are represented by arcs drawn between them. The size of the arc is proportional to the importance of the flow.


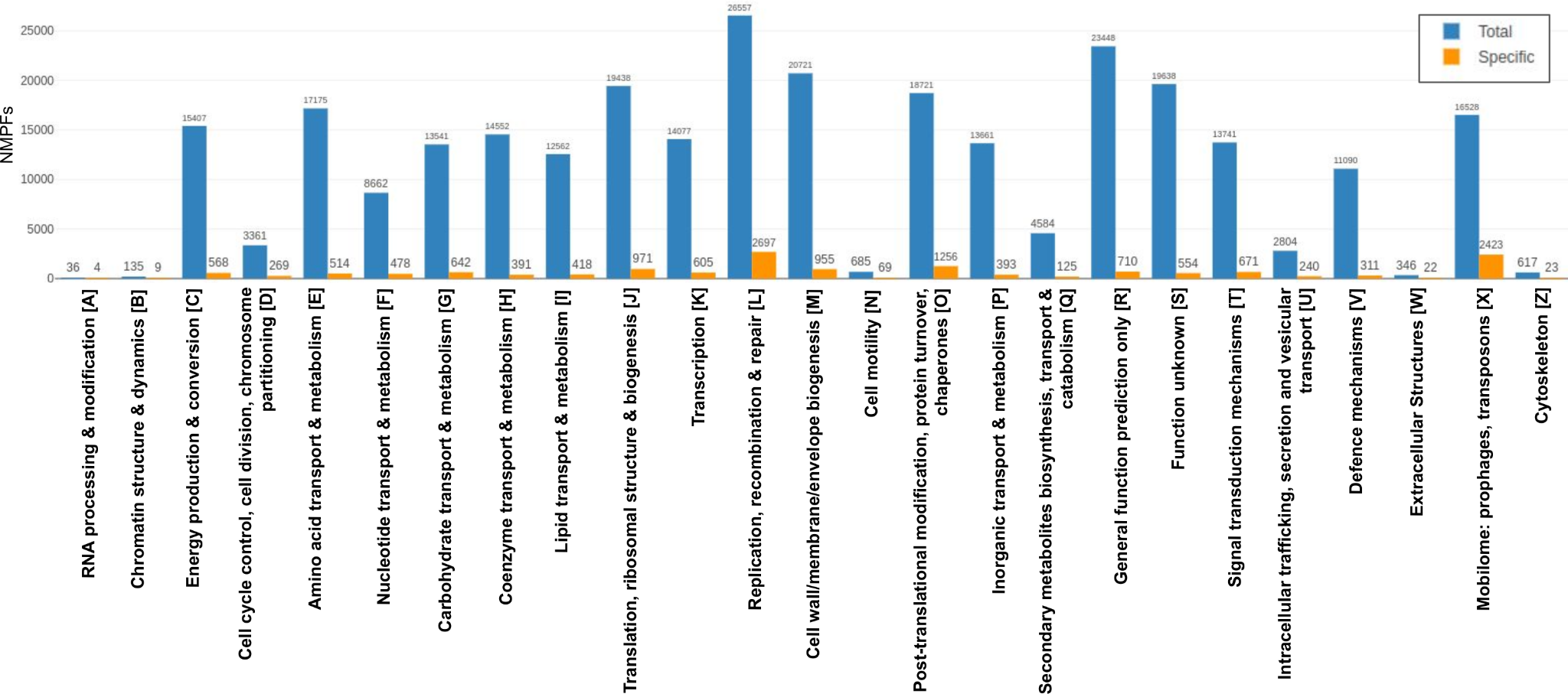


**Fig. S5.** Distribution of total vs specific NMPFs across COG functional categories, as inferred from gene neighborhood analysis. Each category is represented by a blue bar for the total number of associated NMPFs and an orange bar for the category-specific NMPFs, i.e. NMPFs whose gene neighborhood is associated only with this specific functional category. The vertical axis represents the number of NMPFs.

### Supplementary Tables

**Table S1.** Biome distribution in the IMG/M datasets used in this study.

| **Main Ecosystems** | **Ecosystem type** | **Number of Datasets** | **% of Total** |
| --- | --- | --- | --- |
| Environmental (14,540) (75.24%) | Freshwater | 2,277 | 11.78 |
|  | Marine | 3,230 | 16.71 |
|  | Soil | 3,692 | 19.10 |
|  | Other environmental types (air, hot springs etc.) | 5,341 | 27.64 |
| Host-associated (3,867)  (20.01%) | Human | 2,188 | 11.32 |
|  | Non-human mammals | 188 | 0.97 |
|  | Plants | 872 | 4.51 |
|  | Other host-associated (insects, fungi etc.) | 619 | 3.20 |
| Engineered (919)  (4.76%) | Engineered | 919 | 4.76 |
| TOTAL |  | 19,326 | 100 |

**Table S2.** Features of the top 10 NMPFs (largest number of sequences) that are distributed across all ecosystem categories.

| **NMPF** | **Datasets** | **Sequences** | **Dataset type** | **Most common ecosystem** | **Taxonomy** | **3D model**  **(pTM-score)** | **Top SCOPe / PDB biol. assembly hit** | **Top co-occurring Pfam domains** | **Top co-occurring COG functional categories** |
| --- | --- | --- | --- | --- | --- | --- | --- | --- | --- |
| **F000025** | 362 | 5578 | MetaG / MetaT | Soil | Unclassified | Y  (0.82) | LolA-like prokaryotic lipoproteins and lipoprotein localization factor  (SCOPe: d2w7qa_) | Med31, Peptidase_S10, LIM, Ribosomal_S3Ae, RCC1_2 | Translation, ribosomal structure and biogenesis [J], Transcription [K], Amino acid transport and metabolism [E] |
| **F000029** | 12 | 5053 | MetaG | Plants | Eukarya / Unclassified | Y  (0.25) | N/A  (pTM<0.7) | RVT3, Transposase_28 | N/A |
| **F000035** | 46 | 4444 | MetaG | Host-associated | Bacteria / Eukarya / Viruses / Unclassified | Y  (0.35) | N/A  (pTM<0.7) | RVT_1, 7tm_1, Dimer_Tnp_hAT, Pkinase, ion_tans | Signal transduction mechanisms [T], Amino acid transport and metabolism [E], General function prediction only [R], Lipid transport and metabolism [I] |
| **F000036** | 1128 | 4352 | MetaG / MetaT | Soil | Bacteria / Unclassified | Y  (0.50) | N/A  (pTM<0.70) | Glucosamine_iso, GerE, PilZ, Glucokinase, G6PD_C | Carbohydrate transport and metabolism [G], Cell wall/membrane/envelope biogenesis [M], Signal transduction mechanisms [T] |
| **F000037** | 512 | 4230 | MetaG / MetaT | Soil | Bacteria / Viruses / Unclassified | Y  (0.73) | SH2 domain  (SCOPe: d1ayaa_) | ABC_sub_bind, DNA_ligase_A_M, CSD, Ku, TctC | General function prediction only [R], Replication, recombination and repair [L], Energy production and conversion [C] |
| **F000042** | 987 | 3762 | MetaG / MetaT | Soil | Bacteria / Unclassified | Y  (0.67) | N/A  (pTM<0.70) | Hsp20, CSD, HTH_Crp_2, DUF2934, OEP | Pos-ttranslational modification, protein turnover, chaperones [O], Cell wall/membrane/envelope biogenesis [M], Lipid transport and metabolism [I], Translation, ribosomal structure and biogenesis [J], Energy production and conversion [C] |
| **F000043** | 844 | 3655 | MetaG / MetaT | Soil | Bacteria / Unclassified | Y  (0.78) | UBC-like RWD domain  (SCOPe: d1ukxa1) | DNA_topoisoIV, DNA_gyraseB_C, Toprim, DNA_gyraseA_C, HATPase_C | Replication, recombination and repair [L], General function prediction only [R], Nucleotide transport and metabolism [F] |
| **F000048** | 689 | 3365 | MetaG / MetaT | Marine | Viruses / Unclassified | Y  (0.57) | N/A  (pTM<0.7) | Spectrin, PRP21_like_P,  Costars, Chloroa_b-bind, NAD_binding_1 | Cell cycle control, cell division, chromosome partitioning [D] |
| **F000057** | 624 | 3033 | MetaG / MetaT | Marine | Bacteria / Viruses / Unclassified | Y  (0.54) | N/A  (pTM<0.7) | 2OG-FeII_Oxy_3, Laminin_G_3, Sulfotransfer_3, T4_gp9_10, Sulfotransfer_1 | Lipid transport and metabolism [I], Intracellular trafficking, secretion, and vesicular transport [U], Cell cycle control, cell division, chromosome partitioning [D], Replication, recombination and repair [L], Translation, ribosomal structure and biogenesis [J] |
| **F000065** | 823 | 2788 | MetaG / MetaT | Soil | Bacteria / Unclassified | Y  (0.52) | N/A  (pTM<0.7) | HTH_Crp_2, Nramp, Response_reg, CsbD, SnoaL_2 | Inorganic ion transport and metabolism [P], Function unknown [S], Signal transduction mechanisms [T], Coenzyme transport and metabolism [H] |

**Table S3.** Features of the top 20 most prevalent clusters for each ecosystem category (largest number of ecosystem-related datasets per family against the total ecosystem-related datasets).

| **NMPF** | **Dataset Type** | **No. of Sequences** | **Total Family Datasets** | **Most common habitat datasets** | **Most common Habitat percentage in Family (%)** | **Family prevalence in most common habitat (%)** | **Taxonomy** | **3D model** |
| --- | --- | --- | --- | --- | --- | --- | --- | --- |
| ***Freshwater ecosystems*** | | | | | | | | |
| F000324 | MetaG / MetaT | 1299 | 478 | 408 | 85.36 | 17.92 | Unclassified / Bacteria / Viruses | Y |
| F000684 | MetaG / MetaT | 938 | 506 | 399 | 78.85 | 17.52 | Unclassified / Bacteria / Viruses | Y |
| F000450 | MetaG / MetaT | 1126 | 445 | 378 | 84.94 | 16.60 | Unclassified / Bacteria / Viruses | Y |
| F000370 | MetaG / MetaT | 1221 | 468 | 369 | 78.85 | 16.21 | Unclassified / Bacteria / Eukarya / Viruses | Y |
| F000258 | MetaG / MetaT | 1443 | 448 | 359 | 80.13 | 15.77 | Unclassified / Bacteria / Viruses | Y |
| F000652 | MetaG / MetaT | 959 | 392 | 357 | 91.07 | 15.68 | Unclassified / Viruses | Y |
| F000237 | MetaG / MetaT | 1498 | 827 | 354 | 42.81 | 15.55 | Unclassified / Viruses | Y |
| F000311 | MetaG / MetaT | 1326 | 484 | 349 | 72.11 | 15.33 | Unclassified / Bacteria / Viruses | Y |
| F000934 | MetaG / MetaT | 828 | 422 | 347 | 82.23 | 15.24 | Unclassified / Bacteria / Viruses | Y |
| F000473 | MetaG / MetaT | 1097 | 426 | 328 | 77.00 | 14.40 | Unclassified / Bacteria / Viruses | Y |
| F000141 | MetaG / MetaT | 1941 | 387 | 323 | 83.46 | 14.19 | Unclassified / Bacteria / Viruses | Y |
| F000369 | MetaG / MetaT | 1222 | 426 | 322 | 75.59 | 14.14 | Unclassified / Bacteria / Viruses | N |
| F000346 | MetaG / MetaT | 1253 | 402 | 321 | 79.85 | 14.10 | Unclassified / Bacteria / Viruses | Y |
| F000376 | MetaG / MetaT | 1216 | 425 | 321 | 75.53 | 14.10 | Unclassified / Bacteria / Viruses | Y |
| F000264 | MetaG / MetaT | 1424 | 453 | 319 | 70.42 | 14.01 | Unclassified / Bacteria / Viruses | Y |
| F000857 | MetaG / MetaT | 858 | 390 | 319 | 81.79 | 14.01 | Unclassified / Bacteria / Viruses | N |
| F000263 | MetaG / MetaT | 1424 | 453 | 316 | 69.76 | 13.88 | Unclassified / Viruses | Y |
| F000716 | MetaG / MetaT | 923 | 406 | 311 | 76.60 | 13.66 | Unclassified / Bacteria / Viruses | Y |
| F001106 | MetaG / MetaT | 776 | 387 | 309 | 79.84 | 13.57 | Unclassified / Bacteria / Viruses | N |
| F000368 | MetaG / MetaT | 1223 | 417 | 309 | 74.10 | 13.57 | Unclassified / Bacteria / Viruses | Y |
| ***Marine ecosystems*** | | | | | | | | |
| F000075 | MetaG / MetaT | 2622 | 1195 | 701 | 58.66 | 21.70 | Unclassified | N |
| F000055 | MetaG / MetaT | 3096 | 901 | 542 | 60.16 | 16.78 | Unclassified | Y |
| F000107 | MetaG / MetaT | 2222 | 726 | 509 | 70.11 | 15.76 | Unclassified / Viruses | Y |
| F000021 | MetaG / MetaT | 6082 | 704 | 468 | 66.48 | 14.49 | Unclassified | Y |
| F000200 | MetaG / MetaT | 1633 | 634 | 466 | 73.50 | 14.43 | Unclassified / Bacteria / Viruses | Y |
| F000048 | MetaG / MetaT | 3365 | 689 | 454 | 65.89 | 14.06 | Unclassified / Viruses | Y |
| F000155 | MetaG / MetaT | 1877 | 841 | 448 | 53.27 | 13.87 | Unclassified | N |
| F000088 | MetaG / MetaT | 2436 | 736 | 438 | 59.51 | 13.56 | Unclassified | Y |
| F000073 | MetaG / MetaT | 2639 | 624 | 436 | 69.87 | 13.50 | Unclassified | Y |
| F000051 | MetaG / MetaT | 3266 | 638 | 432 | 67.71 | 13.37 | Unclassified / Viruses | Y |
| F000352 | MetaG / MetaT | 1247 | 543 | 401 | 73.85 | 12.41 | Unclassified / Bacteria / Viruses | Y |
| F000049 | MetaG / MetaT | 3277 | 657 | 400 | 60.88 | 12.38 | Unclassified / Eukarya / Viruses | N |
| F000052 | MetaG / MetaT | 3223 | 556 | 399 | 71.76 | 12.35 | Unclassified / Viruses | Y |
| F000302 | MetaG / MetaT | 1337 | 564 | 397 | 70.39 | 12.29 | Unclassified / Bacteria / Viruses | Y |
| F000039 | MetaG / MetaT | 4032 | 554 | 384 | 69.31 | 11.89 | Unclassified / Viruses | Y |
| F000287 | MetaT | 1370 | 514 | 381 | 74.12 | 11.80 | Unclassified / Viruses | N |
| F000139 | MetaT | 1948 | 487 | 372 | 76.39 | 11.52 | Unclassified / Viruses | Y |
| F000379 | MetaG / MetaT | 1211 | 548 | 369 | 67.34 | 11.42 | Unclassified / Bacteria / Viruses | N |
| F000068 | MetaG / MetaT | 2731 | 503 | 369 | 73.36 | 11.42 | Unclassified | Y |
| F000711 | MetaG / MetaT | 925 | 488 | 361 | 73.98 | 11.18 | Unclassified / Viruses | N |
| ***Soil ecosystems*** | | | | | | | | |
| F000036 | MetaG / MetaT | 4352 | 1128 | 595 | 52.75 | 16.12 | Unclassified / Bacteria | Y |
| F000043 | MetaG / MetaT | 3655 | 844 | 539 | 63.86 | 14.60 | Unclassified / Bacteria | Y |
| F000042 | MetaG / MetaT | 3762 | 987 | 499 | 50.56 | 13.52 | Unclassified / Bacteria | Y |
| F000105 | MetaG / MetaT | 2228 | 896 | 493 | 55.02 | 13.35 | Unclassified / Bacteria | Y |
| F000063 | MetaG / MetaT | 2803 | 863 | 481 | 55.74 | 13.03 | Unclassified / Bacteria | Y |
| F000151 | MetaG / MetaT | 1897 | 779 | 473 | 60.72 | 12.81 | Unclassified / Bacteria | Y |
| F000159 | MetaG / MetaT | 1863 | 681 | 469 | 68.87 | 12.70 | Unclassified / Bacteria | Y |
| F000092 | MetaG / MetaT | 2385 | 669 | 436 | 65.17 | 11.81 | Unclassified / Bacteria / Viruses | Y |
| F000268 | MetaG / MetaT | 1411 | 807 | 425 | 52.66 | 11.51 | Unclassified / Bacteria | Y |
| F000157 | MetaG / MetaT | 1872 | 706 | 420 | 59.49 | 11.38 | Unclassified / Bacteria | Y |
| F000174 | MetaG / MetaT | 1764 | 703 | 415 | 59.03 | 11.24 | Unclassified / Bacteria | Y |
| F000218 | MetaG / MetaT | 1552 | 710 | 414 | 58.31 | 11.21 | Unclassified / Bacteria | Y |
| F000072 | MetaG / MetaT | 2651 | 804 | 411 | 51.12 | 11.13 | Unclassified / Bacteria / Viruses | Y |
| F000165 | MetaG / MetaT | 1824 | 594 | 401 | 67.51 | 10.86 | Unclassified / Bacteria | Y |
| F000209 | MetaG / MetaT | 1592 | 778 | 399 | 51.29 | 10.81 | Unclassified / Bacteria | Y |
| F000171 | MetaG / MetaT | 1783 | 667 | 399 | 59.82 | 10.81 | Unclassified / Bacteria | Y |
| F000083 | MetaG / MetaT | 2471 | 536 | 395 | 73.69 | 10.70 | Unclassified / Bacteria | Y |
| F000132 | MetaG / MetaT | 1972 | 691 | 394 | 57.02 | 10.67 | Unclassified / Bacteria | Y |
| F000364 | MetaG / MetaT | 1229 | 716 | 389 | 54.33 | 10.54 | Unclassified / Bacteria | Y |
| F000198 | MetaG / MetaT | 1648 | 708 | 389 | 54.94 | 10.54 | Unclassified / Bacteria | Y |
| ***Human ecosystems*** | | | | | | | | |
| F018385 | MetaG | 235 | 197 | 126 | 63.96 | 5.76 | Unclassified / Bacteria / Viruses | N |
| F049707 | MetaG | 146 | 134 | 120 | 89.55 | 5.48 | Unclassified / Bacteria / Viruses | N |
| F059076 | MetaG | 134 | 118 | 109 | 92.37 | 4.98 | Unclassified / Bacteria | N |
| F030786 | MetaG | 184 | 171 | 109 | 63.74 | 4.98 | Unclassified / Bacteria | N |
| F022002 | MetaG | 216 | 110 | 106 | 96.36 | 4.84 | Unclassified / Bacteria | N |
| F064818 | MetaG | 128 | 123 | 106 | 86.18 | 4.84 | Unclassified / Bacteria | N |
| F000085 | MetaG / MetaT | 2467 | 532 | 105 | 19.74 | 4.80 | Unclassified | Y |
| F036281 | MetaG | 170 | 163 | 102 | 62.58 | 4.66 | Unclassified / Bacteria / Viruses | N |
| F027205 | MetaG | 195 | 171 | 100 | 58.48 | 4.57 | Unclassified / Bacteria / Viruses | N |
| F047127 | MetaG | 150 | 147 | 100 | 68.03 | 4.57 | Unclassified / Bacteria | N |
| F063778 | MetaG | 129 | 106 | 99 | 93.40 | 4.52 | Unclassified / Bacteria | N |
| F101359 | MetaG | 102 | 100 | 93 | 93.00 | 4.25 | Unclassified / Bacteria | N |
| F085821 | MetaG | 111 | 98 | 92 | 93.88 | 4.20 | Unclassified / Bacteria | N |
| F051212 | MetaG | 144 | 144 | 90 | 62.50 | 4.11 | Unclassified / Bacteria | N |
| F054111 | MetaG | 140 | 119 | 90 | 75.63 | 4.11 | Unclassified / Bacteria | N |
| F095632 | MetaG | 105 | 97 | 89 | 91.75 | 4.07 | Unclassified / Bacteria / Viruses | N |
| F043991 | MetaG | 155 | 138 | 89 | 64.49 | 4.07 | Unclassified / Bacteria / Viruses | N |
| F042387 | MetaG | 158 | 156 | 88 | 56.41 | 4.02 | Unclassified / Bacteria | N |
| F057446 | MetaG | 136 | 135 | 87 | 64.44 | 3.98 | Unclassified / Bacteria | N |
| F040685 | MetaG | 161 | 124 | 87 | 70.16 | 3.98 | Unclassified / Bacteria | N |
| ***Mammal ecosystems*** | | | | | | | | |
| F006970 | MetaG / MetaT | 361 | 90 | 89 | 98.89 | 47.34 | Unclassified | N |
| F002486 | MetaG / MetaT | 555 | 88 | 88 | 100.00 | 46.81 | Unclassified | N |
| F006460 | MetaG / MetaT | 372 | 92 | 88 | 95.65 | 46.81 | Unclassified | N |
| F003473 | MetaG / MetaT | 484 | 87 | 87 | 100.00 | 46.28 | Unclassified / Bacteria | N |
| F008062 | MetaG / MetaT | 340 | 88 | 85 | 96.59 | 45.21 | Unclassified | N |
| F008407 | MetaG / MetaT | 333 | 83 | 83 | 100.00 | 44.15 | Unclassified | N |
| F008930 | MetaG / MetaT | 325 | 83 | 83 | 100.00 | 44.15 | Unclassified / Eukarya | N |
| F005942 | MetaG / MetaT | 385 | 81 | 81 | 100.00 | 43.09 | Unclassified / Eukarya | N |
| F004268 | MetaG / MetaT | 446 | 81 | 81 | 100.00 | 43.09 | Unclassified | N |
| F008732 | MetaG / MetaT | 329 | 81 | 81 | 100.00 | 43.09 | Unclassified / Eukarya | N |
| F008855 | MetaG / MetaT | 327 | 83 | 81 | 97.59 | 43.09 | Unclassified | N |
| F012836 | MetaG / MetaT | 277 | 81 | 80 | 98.77 | 42.55 | Unclassified | N |
| F008287 | MetaG / MetaT | 335 | 80 | 80 | 100.00 | 42.55 | Unclassified | N |
| F004717 | MetaG / MetaT | 426 | 80 | 80 | 100.00 | 42.55 | Unclassified | N |
| F008288 | MetaG / MetaT | 335 | 79 | 79 | 100.00 | 42.02 | Unclassified | N |
| F019925 | MetaG / MetaT | 227 | 79 | 79 | 100.00 | 42.02 | Unclassified | N |
| F004661 | MetaG / MetaT | 428 | 79 | 79 | 100.00 | 42.02 | Unclassified | N |
| F015040 | MetaG / MetaT | 258 | 79 | 78 | 98.73 | 41.49 | Unclassified | N |
| F004857 | MetaG / MetaT | 421 | 78 | 78 | 100.00 | 41.49 | Unclassified / Bacteria / Eukarya | N |
| F003062 | MetaG / MetaT | 509 | 77 | 77 | 100.00 | 40.96 | Unclassified | Y |
| ***Plant ecosystems*** | | | | | | | | |
| F000224 | MetaG / MetaT | 1528 | 670 | 144 | 21.49 | 16.51 | Unclassified / Bacteria / Eukarya | Y |
| F000268 | MetaG / MetaT | 1411 | 807 | 126 | 15.61 | 14.45 | Unclassified / Bacteria | Y |
| F001033 | MetaG / MetaT | 799 | 557 | 126 | 22.62 | 14.45 | Unclassified / Bacteria / Archaea | N |
| F000553 | MetaG / MetaT | 1032 | 501 | 125 | 24.95 | 14.33 | Unclassified / Bacteria | Y |
| F001131 | MetaG / MetaT | 768 | 407 | 125 | 30.71 | 14.33 | Unclassified / Bacteria | Y |
| F000569 | MetaG / MetaT | 1018 | 759 | 125 | 16.47 | 14.33 | Unclassified / Bacteria | N |
| F000116 | MetaG | 2132 | 730 | 125 | 17.12 | 14.33 | Unclassified / Bacteria | Y |
| F000493 | MetaG / MetaT | 1078 | 418 | 120 | 28.71 | 13.76 | Unclassified / Bacteria / Eukarya / Viruses | Y |
| F001203 | MetaG / MetaT | 748 | 282 | 119 | 42.20 | 13.65 | Unclassified / Bacteria | Y |
| F000690 | MetaG / MetaT | 935 | 463 | 118 | 25.49 | 13.53 | Unclassified / Bacteria | Y |
| F000382 | MetaG / MetaT | 1210 | 419 | 117 | 27.92 | 13.42 | Unclassified / Bacteria / Eukarya / Viruses | Y |
| F002895 | MetaG | 522 | 300 | 117 | 39.00 | 13.42 | Unclassified / Bacteria | N |
| F003272 | MetaG / MetaT | 496 | 305 | 116 | 38.03 | 13.30 | Unclassified / Bacteria | N |
| F000135 | MetaG / MetaT | 1961 | 816 | 116 | 14.22 | 13.30 | Unclassified / Bacteria | Y |
| F000616 | MetaG / MetaT | 983 | 425 | 115 | 27.06 | 13.19 | Unclassified / Bacteria / Viruses | Y |
| F000463 | MetaG / MetaT | 1107 | 646 | 113 | 17.49 | 12.96 | Unclassified / Bacteria | Y |
| F001251 | MetaG / MetaT | 737 | 400 | 113 | 28.25 | 12.96 | Unclassified / Bacteria | Y |
| F000638 | MetaG | 968 | 117 | 111 | 94.87 | 12.73 | Unclassified | Y |
| F000466 | MetaG / MetaT | 1105 | 506 | 109 | 21.54 | 12.50 | Unclassified / Bacteria | N |
| F001288 | MetaG / MetaT | 730 | 367 | 109 | 29.70 | 12.50 | Unclassified / Bacteria | N |
| ***Other host-associated ecosystems*** | | | | | | | | |
| F000950 | MetaG | 822 | 89 | 88 | 98.88 | 14.22 | Unclassified / Eukarya / Viruses | Y |
| F000488 | MetaG | 1084 | 83 | 83 | 100.00 | 13.41 | Unclassified / Eukarya / Viruses | Y |
| F008983 | MetaG | 324 | 78 | 78 | 100.00 | 12.60 | Unclassified / Eukarya | N |
| F005805 | MetaG | 389 | 76 | 76 | 100.00 | 12.28 | Unclassified / Eukarya / Viruses | N |
| F008928 | MetaG | 325 | 76 | 76 | 100.00 | 12.28 | Unclassified / Eukarya / Viruses | N |
| F008285 | MetaG | 335 | 75 | 75 | 100.00 | 12.12 | Unclassified / Eukarya / Viruses | N |
| F003922 | MetaG | 461 | 74 | 74 | 100.00 | 11.95 | Unclassified / Eukarya / Viruses | Y |
| F000084 | MetaG | 2468 | 122 | 74 | 60.66 | 11.95 | Unclassified | Y |
| F024689 | MetaG | 204 | 69 | 69 | 100.00 | 11.15 | Unclassified / Eukarya | N |
| F000202 | MetaG | 1620 | 111 | 68 | 61.26 | 10.99 | Unclassified / Viruses | Y |
| F012417 | MetaG | 280 | 64 | 64 | 100.00 | 10.34 | Unclassified / Eukarya / Viruses | N |
| F035582 | MetaG | 171 | 63 | 63 | 100.00 | 10.18 | Unclassified / Eukarya | N |
| F031724 | MetaG | 181 | 63 | 63 | 100.00 | 10.18 | Unclassified / Eukarya / Viruses | Y |
| F000721 | MetaG | 922 | 98 | 62 | 63.27 | 10.02 | Unclassified / Viruses | Y |
| F065291 | MetaG | 127 | 61 | 61 | 100.00 | 9.85 | Unclassified / Eukarya / Viruses | N |
| F013487 | MetaG | 270 | 61 | 61 | 100.00 | 9.85 | Unclassified / Viruses | N |
| F049334 | MetaG | 146 | 61 | 61 | 100.00 | 9.85 | Unclassified / Eukarya / Viruses | N |
| F010439 | MetaG | 303 | 60 | 60 | 100.00 | 9.69 | Unclassified / Eukarya / Viruses | N |
| F065290 | MetaG | 127 | 59 | 59 | 100.00 | 9.53 | Unclassified / Eukarya | N |
| F023497 | MetaG | 209 | 59 | 59 | 100.00 | 9.53 | Unclassified / Eukarya | Y |
| ***Engineered ecosystems*** | | | | | | | | |
| F000388 | MetaG / MetaT | 1201 | 425 | 155 | 36.47 | 16.87 | Unclassified / Bacteria / Viruses | Y |
| F005744 | MetaG / MetaT | 391 | 169 | 153 | 90.53 | 16.65 | Unclassified / Viruses | N |
| F003987 | MetaG / MetaT | 458 | 161 | 146 | 90.68 | 15.89 | Unclassified / Bacteria / Archaea / Viruses | Y |
| F004383 | MetaG / MetaT | 440 | 164 | 145 | 88.41 | 15.78 | Unclassified / Archaea / Viruses | Y |
| F013091 | MetaG / MetaT | 274 | 145 | 144 | 99.31 | 15.67 | Unclassified | N |
| F020914 | MetaG / MetaT | 221 | 142 | 139 | 97.89 | 15.13 | Unclassified | N |
| F015738 | MetaG / MetaT | 252 | 147 | 133 | 90.48 | 14.47 | Unclassified / Archaea | N |
| F012026 | MetaG / MetaT | 284 | 130 | 127 | 97.69 | 13.82 | Unclassified / Bacteria / Viruses | N |
| F015605 | MetaG / MetaT | 253 | 132 | 126 | 95.45 | 13.71 | Unclassified | N |
| F031111 | MetaG / MetaT | 183 | 119 | 119 | 100.00 | 12.95 | Unclassified / Bacteria | N |
| F070158 | MetaG / MetaT | 123 | 118 | 117 | 99.15 | 12.73 | Unclassified / Eukarya / Viruses | N |
| F011593 | MetaG / MetaT | 289 | 125 | 115 | 92.00 | 12.51 | Unclassified / Bacteria / Viruses | N |
| F021528 | MetaG / MetaT | 218 | 123 | 115 | 93.50 | 12.51 | Unclassified / Viruses | N |
| F017851 | MetaG / MetaT | 238 | 126 | 114 | 90.48 | 12.40 | Unclassified / Archaea | N |
| F022893 | MetaG / MetaT | 212 | 115 | 112 | 97.39 | 12.19 | Unclassified / Bacteria / Viruses | N |
| F031881 | MetaG / MetaT | 181 | 114 | 111 | 97.37 | 12.08 | Unclassified / Viruses | N |
| F009968 | MetaG / MetaT | 310 | 128 | 110 | 85.94 | 11.97 | Unclassified / Bacteria / Viruses | N |
| F029768 | MetaG / MetaT | 187 | 117 | 110 | 94.02 | 11.97 | Unclassified / Viruses | N |
| F016798 | MetaG / MetaT | 244 | 112 | 110 | 98.21 | 11.97 | Unclassified / Bacteria / Viruses | N |
| F018007 | MetaG / MetaT | 237 | 144 | 110 | 76.39 | 11.97 | Unclassified / Bacteria / Viruses | N |

**Table S4.** Features of the top 10 largest ecosystem-specific clusters in (largest number of sequences).

| **Cluster Number** | **No. of Datasets** | **No. of Scaffolds** | **No. of Sequences** | **Dataset type** | **Taxonomy** | **Available 3D model** |
| --- | --- | --- | --- | --- | --- | --- |
| ***Freshwater*** | | | | | | |
| **F000320** | 83 | 1305 | 1306 | MetaG/MetaT | Unclassified | Y |
| **F000350** | 112 | 1248 | 1248 | MetaG | Eukarya / Viruses / Bacteria / Unclassified | N |
| **F000449** | 81 | 1126 | 1126 | MetaG/MetaT | Viruses / Unclassified | Y |
| **F000815** | 168 | 879 | 879 | MetaG/MetaT | Bacteria / Viruses / Unclassified | N |
| **F000924** | 104 | 832 | 832 | MetaG | Eukarya / Viruses / Unclassified | N |
| **F000951** | 167 | 822 | 822 | MetaG/MetaT | Unclassified | N |
| **F001003** | 85 | 808 | 808 | MetaG/MetaT | Viruses / Unclassified | N |
| **F001030** | 102 | 800 | 800 | MetaG | Eukarya / Viruses / Unclassified | N |
| **F001098** | 144 | 778 | 778 | MetaG/MetaT | Unclassified | N |
| **F001258** | 75 | 735 | 735 | MetaG | Viruses / Unclassified | N |
| ***Marine*** | | | | | | |
| **F000067** | 196 | 2732 | 2732 | MetaG/MetaT | Viruses / Unclassified | N |
| **F000196** | 314 | 1657 | 1657 | MetaT | Unclassified | N |
| **F000361** | 35 | 1230 | 1231 | MetaG/MetaT | Eukarya / Viruses / Bacteria / Unclassified | Y |
| **F000423** | 211 | 1156 | 1156 | MetaG/MetaT | Bacteria / Viruses / Unclassified | Y |
| **F000467** | 207 | 1104 | 1104 | MetaT | Viruses / Unclassified | N |
| **F000480** | 330 | 1090 | 1090 | MetaT | Viruses / Unclassified | N |
| **F000538** | 209 | 1043 | 1043 | MetaG/MetaT | Bacteria / Unclassified | Y |
| **F000567** | 225 | 1019 | 1020 | MetaG/MetaT | Bacteria / Viruses / Unclassified | N |
| **F000615** | 297 | 979 | 984 | MetaG/MetaT | Bacteria / Viruses / Unclassified | Y |
| **F000639** | 204 | 968 | 968 | MetaG | Bacteria / Viruses / Unclassified | Y |
| ***Soil*** | | | | | | |
| **F001925** | 70 | 440 | 616 | MetaG | Viruses / Unclassified | Y |
| **F001980** | 209 | 608 | 608 | MetaG/MetaT | Bacteria / Viruses / Archaea / Unclassified | N |
| **F002354** | 100 | 356 | 567 | MetaG | Bacteria / Viruses / Archaea / Unclassified | Y |
| **F002367** | 154 | 414 | 566 | MetaG/MetaT | Viruses / Unclassified | N |
| **F002498** | 204 | 553 | 553 | MetaG/MetaT | Bacteria / Arcahea / Unclassified | N |
| **F002607** | 188 | 543 | 543 | MetaG/MetaT | Bacteria / Unclassified | N |
| **F002941** | 196 | 518 | 518 | MetaG/MetaT | Bacteria / Viruses / Archaea / Unclassified | N |
| **F002983** | 192 | 515 | 515 | MetaG/MetaT | Bacteria / Arcahea / Unclassified | N |
| **F003036** | 132 | 511 | 511 | MetaG/MetaT | Eukarya / Unclassified | Y |
| **F003305** | 171 | 494 | 494 | MetaG/MetaT | Bacteria / Viruses / Archaea / Unclassified | N |
| ***Plant*** | | | | | | |
| **F000056** | 9 | 2971 | 3057 | MetaG | Unclassified | Y |
| **F000129** | 110 | 1935 | 1994 | MetaG | Unclassified | Y |
| **F000130** | 106 | 1627 | 1990 | MetaG | Viruses / Unclassified | Y |
| **F000422** | 80 | 1157 | 1157 | MetaG | Eukarya / Unclassified | Y |
| **F000433** | 27 | 1141 | 1147 | MetaG | Eukarya / Bacteria / Unclassified | Y |
| **F000434** | 88 | 1147 | 1147 | MetaG | Bacteria / Viruses / Unclassified | Y |
| **F000541** | 93 | 1042 | 1042 | MetaG | Unclassified | Y |
| **F000593** | 90 | 1000 | 1000 | MetaG | Bacteria / Viruses / Unclassified | Y |
| **F000755** | 77 | 905 | 905 | MetaG | Unclassified | Y |
| **F000907** | 95 | 841 | 841 | MetaG | Eukarya / Bacteria / Unclassified | Y |
| ***Human Gut*** | | | | | | |
| **F022002** | 110 | 153 | 216 | MetaG | Bacteria / Unclassified | N |
| **F030786** | 171 | 184 | 184 | MetaG | Bacteria / Unclassified | N |
| **F033081** | 149 | 178 | 178 | MetaG | Bacteria / Unclassified | N |
| **F039147** | 164 | 164 | 164 | MetaG | Bacteria / Unclassified | N |
| **F040149** | 157 | 162 | 162 | MetaG | Bacteria / Unclassified | N |
| **F040685** | 124 | 139 | 161 | MetaG | Bacteria / Unclassified | N |
| **F041208** | 160 | 160 | 160 | MetaG | Bacteria / Unclassified | N |
| **F041827** | 127 | 143 | 159 | MetaG | Bacteria / Unclassified | N |
| **F042387** | 156 | 158 | 158 | MetaG | Bacteria / Unclassified | N |
| **F042910** | 157 | 157 | 157 | MetaG | Bacteria / Unclassified | N |
| ***Human Oral*** | | | | | | |
| **F022002** | 110 | 153 | 216 | MetaG | Bacteria / Unclassified | N |
| **F030786** | 171 | 184 | 184 | MetaG | Bacteria / Unclassified | N |
| **F033081** | 149 | 178 | 178 | MetaG | Bacteria / Unclassified | N |
| **F040149** | 157 | 162 | 162 | MetaG | Bacteria / Unclassified | N |
| **F040685** | 124 | 139 | 161 | MetaG | Bacteria / Unclassified | N |
| **F041827** | 127 | 143 | 159 | MetaG | Bacteria / Unclassified | N |
| **F042387** | 156 | 158 | 158 | MetaG | Bacteria / Unclassified | N |
| **F043235** | 156 | 156 | 156 | MetaG | Bacteria / Unclassified | N |
| **F043990** | 145 | 155 | 155 | MetaG | Bacteria / Unclassified | N |
| **F045567** | 149 | 152 | 152 | MetaG | Bacteria / Viruses / Unclassified | N |

**Table S5.** Classification of protein clusters into Metagenome (MetaG)-only, Metatranscriptome (MetaT)-only and Mixed MetaG/MetaT, based on their source environmental datasets for all cluster size cut-offs (≥3, ≥25, ≥50, ≥75 and ≥100 members).

| **Cluster size** | **≥3 members** | **≥25 members** | **≥50 members** | **≥75 members** | **≥100 members** |
| --- | --- | --- | --- | --- | --- |
| Total Clusters (NMPFs) | 64,149,288 | 1,501,861 | 428,910 | 200,075 | 106,198 |
| MetaG-only | 53,585,767 | 918,568 | 211,928 | 82,859 | 38,292 |
| MetaT-only | 4,503,437 | 64,323 | 14,810 | 6,717 | 3,720 |
| Mixed MetaG/MetaT | 6,060,084 | 518,970 | 202,172 | 110,499 | 64,186 |

**Table S6.** Features of the top 10 clusters with the widest dataset distribution (largest number of datasets).

| **Cluster** | **Datasets** | **Total Prevalence** | **Habitat Specific Prevalence** | **Sequences** | **Dataset type** | **Most common habitat** | **Taxonomy** | **3D model** |
| --- | --- | --- | --- | --- | --- | --- | --- | --- |
| F000075 | 1195 | 6% | 21.70% | 2622 | MetaG/MetaT | Marine | Unclassified | N |
| F000036 | 1128 | 5.7% | 16.12% | 4352 | MetaG/MetaT | Soil | Bacteria / Unclassified | Y |
| F000042 | 987 | 5% | 13.52% | 3762 | MetaG/MetaT | Soil | Bacteria / Unclassified | Y |
| F000055 | 901 | 4.6% | 16.78% | 3096 | MetaG/MetaT | Marine | Unclassified | Y |
| F000105 | 896 | 4.6% | 13.35% | 2228 | MetaG/MetaT | Soil | Bacteria / Unclassified | Y |
| F000063 | 863 | 4.4% | 13.03% | 2803 | MetaG/MetaT | Soil | Bacteria / Unclassified | Y |
| F000043 | 844 | 4.3% | 14.60% | 3655 | MetaG/MetaT | Soil | Bacteria / Unclassified | Y |
| F000155 | 841 | 4.3% | 13.97% | 1877 | MetaG/MetaT | Marine | Unclassified | N |
| F000078 | 829 | 4.2% | 10.46% | 2550 | MetaG/MetaT | Soil | Bacteria / Unclassified | Y |
| F000237 | 827 | 4.2% | 15.55% | 1498 | MetaG/MetaT | Freshwater | Unclassified | Y |

**Table S7**. Distribution of scaffolds with only novel protein genes.

| **Gene Count** | **Number of Scaffolds** | **Sample Categories** | **Associated Habitats** |
| --- | --- | --- | --- |
| 1 | 3700126 | Metagenome (67.40%), Metatranscriptome (32.60% ) | Soil (40.96%), Marine (26.48%), Freshwater (11.52%), Plants (9.33%), Host-associated (5.21%) Other Environmental (4.46%), Mammals (1.17%), Engineered (0.44%), Human (0.43%) |
| 2 | 164133 | Metagenome (94.27%), Metatranscriptome (5.73%) | Soil (37.62%), Marine (21.73%), Freshwater (21.57%), Plants (8.09%), Other Environmental (6.73%), Host-associated (3.61%), Engineered (0.52%), Human (0.09%), Mammals (0.03%) |
| 3 | 7417 | Metagenome (96.63%), Metatranscriptome (3.37%) | Marine (30.71%), Freshwater (29.94%), Soil (23.07%), Other Environmental (8.13%), Plants (5.60%), Host-associated (2.09%), Engineered (0.38%), Human (0.07%), Mammals (0.01%) |
| 4 | 673 | Metagenome (97.18%), Metatranscriptome (2.82%) | Freshwater (36.70%), Marine (26.75%), Soil (19.02%), Other Environmental (9.66%), Plants (5.05%), Host-associated (2.53%), Human (0.15%), Engineered (0.15%) |
| 5 | 107 | Metagenome (99.06%), Metatranscriptome (0.94%) | Freshwater (31.77%), Marine (19.63%), Soil (18.69%), Other Environmental (14.02%), Plants (9.35%), Host-associated (6.54%) |
| 6 | 21 | Metagenome (95.24%), Metatranscriptome (4.76%) | Freshwater (42.86%), Marine (23.81%), Host-associated (14.29%), Soil (9.52%), Human (4.76%), Other Environmental (4.76%) |
| 7 | 5 | Metagenome (100%) | Freshwater (60%), Soil (20%), Host-associated (20%) |
| 8 | 2 | Metagenome (100%) | Host-associated (100%) |
| 9 | 1 | Metagenome (100%) | Freshwater (100%) |

**Table S8**. Largest scaffolds with only novel protein genes, having six or more genes per scaffold (top 29 scaffolds from previous table).

| **Taxon OID** | **Scaffold ID** | **Scaffold Length (bps)** | **Gene count** | **Sample Type** | **Habitat** | **Scaffold Taxonomy** |
| --- | --- | --- | --- | --- | --- | --- |
| 3300020483 | Ga0207418_101678 | 1922 | 9 | MetaG | Freshwater | Viral |
| 3300027864 | Ga0209755_10550187 | 1023 | 8 | MetaG | Host-associated (others) | Viral |
| 3300028325 | Ga0268261_10145580 | 2151 | 8 | MetaG | Host-associated (others) | Eukaryotic |
| 2199352003 | 2199785623 | 1303 | 7 | MetaG | Freshwater | Unclassified |
| 3300008107 | Ga0114340_1055121 | 1726 | 7 | MetaG | Freshwater | Viral |
| 3300012209 | Ga0137379_10159237 | 2169 | 7 | MetaG | Soil | Archaeal |
| 3300017988 | Ga0181520_10039359 | 4725 | 7 | MetaG | Freshwater | Bacterial |
| 3300027864 | Ga0209755_10183635 | 2100 | 7 | MetaG | Host-associated (others) | Unclassified |
| 3300004799 | Ga0058863_11919424 | 1588 | 6 | MetaT | Rhizosphere | Bacterial |
| 3300005662 | Ga0078894_10384957 | 1268 | 6 | MetaG | Marine | Viral |
| 3300005747 | Ga0076924_1070370 | 1798 | 6 | MetaG | Marine | Archaeal |
| 3300006735 | Ga0098038_1064474 | 1305 | 6 | MetaG | Marine | Viral |
| 3300010162 | Ga0131853_10666798 | 872 | 6 | MetaG | Host-associated (others) | Unclassified |
| 3300010162 | Ga0131853_10678701 | 861 | 6 | MetaG | Host-associated (others) | Unclassified |
| 3300010162 | Ga0131853_10902540 | 694 | 6 | MetaG | Host-associated (others) | Unclassified |
| 3300012918 | Ga0137396_10151935 | 1686 | 6 | MetaG | Soil | Archaeal |
| 3300019784 | Ga0181359_1021806 | 2431 | 6 | MetaG | Soil | Viral |
| 3300019784 | Ga0181359_1040024 | 1816 | 6 | MetaG | Freshwater | Viral |
| 3300019784 | Ga0181359_1048179 | 1645 | 6 | MetaG | Freshwater | Viral |
| 3300020151 | Ga0211736_10198849 | 1305 | 6 | MetaG | Freshwater | Viral |
| 3300020151 | Ga0211736_10855783 | 1839 | 6 | MetaG | Freshwater | Viral |
| 3300020504 | Ga0208484_1002996 | 2738 | 6 | MetaG | Marine | Viral |
| 3300020695 | Ga0214190_1003563 | 2236 | 6 | MetaG | Freshwater | Unclassified |
| 3300021915 | Ga0214477_105478 | 1651 | 6 | MetaG | Freshwater | Unclassified |
| 3300022735 | Ga0214478_106373 | 2090 | 6 | MetaG | Freshwater | Unclassified |
| 3300022861 | Ga0224528_1012170 | 2512 | 6 | MetaG | Marine | Unclassified |
| 3300025141 | Ga0209756_1058795 | 1830 | 6 | MetaG | Marine | Unclassified |
| 3300028536 | Ga0137415_10132790 | 2332 | 6 | MetaG | Environmental (others) | Unclassified |
| 3300033984 | Ga0334989_0156313 | 1264 | 6 | MetaG | Freshwater | Unclassified |

**Table S9.** Properties of the top 20 longest scaffolds containing both known and novel protein genes.

| **Taxon OID** | **Scaffold ID** | **Length (bps)** | **Total Gene Count** | **Novel Gene Count** | **Taxonomy** | **Habitat** |
| --- | --- | --- | --- | --- | --- | --- |
| 3300022557 | Ga0212123_10000001 | 5,123,848 | 4,302 | 1 | Bacteria | Thermal springs |
| 3300034268 | Ga0372943_0000001 | 4,022,384 | 3,912 | 3 | Bacteria | Soil |
| 3300028794 | Ga0307515_10000001 | 4,259,510 | 3,695 | 1 | Bacteria | Plants |
| 3300025924 | Ga0207694_10000001 | 4,078,485 | 3,652 | 29 | Bacteria | Plants |
| 3300028379 | Ga0268266_10000001 | 4,040,580 | 3,628 | 1 | Bacteria | Plants |
| 3300021388 | Ga0213875_10000001 | 2,793,540 | 2,688 | 10 | Unclassified | Plants |
| 3300032770 | Ga0335085_10000001 | 3,096,523 | 2,639 | 11 | Unclassified | Soil |
| 3300028648 | Ga0268299_1000001 | 2,680,031 | 2,600 | 6 | Bacteria | Engineered |
| 3300033402 | Ga0326728_10000002 | 2,463,942 | 2,574 | 9 | Unclassified | Peat |
| 3300031898 | Ga0315312_1000001 | 2,229,620 | 2,533 | 5 | Unclassified | Freshwater |
| 3300031753 | Ga0307477_10000001 | 2,532,651 | 2,497 | 1 | Bacteria | Soil |
| 3300028028 | Ga0265292_1000001 | 2,887,874 | 2,402 | 1 | Bacteria | Engineered |
| 3300033977 | Ga0314861_0000001 | 2,159,684 | 2,366 | 3 | Unclassified | Soil |
| 3300033402 | Ga0326728_10000001 | 2,540,896 | 2,197 | 5 | Bacteria | Peat |
| 3300024353 | Ga0209979_1000001 | 2,069,247 | 2,178 | 1 | Unclassified | Marine |
| 3300027869 | Ga0209579_10000001 | 2,171,361 | 2,166 | 2 | Bacteria | Soil |
| 3300031898 | Ga0315312_1000002 | 1,945,029 | 2,126 | 2 | Unclassified | Freshwater |
| 3300031898 | Ga0315312_1000003 | 1,943,968 | 2,106 | 14 | Unclassified | Freshwater |
| 3300025226 | Ga0209674_100003 | 2,196,646 | 1,969 | 11 | Bacteria | Plants |
| 3300022557 | Ga0212123_10000002 | 2,195,071 | 1,923 | 42 | Bacteria | Thermal springs |

**Table S10**. Properties of the top 20 scaffolds containing both known and novel protein genes, that have the most novel genes.

| **Taxon OID** | **Scaffold ID** | **Length (bps)** | **Total Gene Count** | **Novel Gene Count** | **Taxonomy** | **Habitat** |
| --- | --- | --- | --- | --- | --- | --- |
| 3300025922 | Ga0207646_10000001 | 1,242,027 | 1332 | 93 | Unclassified | Soil |
| 3300023179 | Ga0214923_10000007 | 295,831 | 377 | 51 | Viruses | Freshwater |
| 3300025922 | Ga0207646_10000003 | 537,256 | 589 | 42 | Unclassified | Soil |
| 3300022557 | Ga0212123_10000002 | 2,195,071 | 1923 | 42 | Acidobacteria | Freshwater (Thermal Springs) |
| 3300031447 | Ga0272435_1000023 | 261,512 | 304 | 38 | Unclassified | Soil (Endoliths) |
| 3300027805 | Ga0209229_10000004 | 91,329 | 146 | 37 | Unclassified | Freshwater |
| 3300027763 | Ga0209088_10000013 | 171,916 | 226 | 36 | Viruses | Freshwater |
| 3300027763 | Ga0209088_10000011 | 188,747 | 273 | 36 | Viruses | Freshwater |
| 3300022524 | Ga0224534_1000001 | 452,495 | 479 | 36 | Unclassified | Peat |
| 3300007735 | Ga0104988_10971 | 52,305 | 116 | 36 | Unclassified | Freshwater |
| 3300027733 | Ga0209297_1000035 | 115,006 | 184 | 35 | Viruses | Freshwater |
| 3300027741 | Ga0209085_1000015 | 115,655 | 184 | 34 | Viruses | Freshwater |
| 3300031453 | Ga0272425_1000036 | 219,672 | 243 | 33 | Unclassified | Soil (Endoliths) |
| 3300023184 | Ga0214919_10000102 | 133,340 | 206 | 33 | Viruses | Freshwater |
| 3300023174 | Ga0214921_10000448 | 76,758 | 147 | 33 | Unclassified | Freshwater |
| 3300009154 | Ga0114963_10000010 | 115,658 | 181 | 32 | Viruses | Freshwater |
| 3300033402 | Ga0326728_10000003 | 2,112,322 | 1843 | 31 | Acidobacteriaceae | Peat |
| 3300027733 | Ga0209297_1000004 | 358,344 | 413 | 31 | Viruses | Freshwater |
| 3300023174 | Ga0214921_10000016 | 349,716 | 484 | 31 | Viruses | Freshwater |
| 3300022752 | Ga0214917_10000045 | 132,273 | 244 | 31 | Viruses | Freshwater |

**Table S11.** Properties of the top 20 metatranscriptome scaffolds containing both known and novel protein genes that have the most novel genes.

| **Taxon OID** | **Scaffold ID** | **Length (bps)** | **Total Gene Count** | **Novel Gene Count** | **Taxonomy** | **Habitat** |
| --- | --- | --- | --- | --- | --- | --- |
| 3300004139 | Ga0058897_11189883 | 15739 | 41 | 20 | Candidatus Bathyarchaeota archaeon | Terrestrial |
| 3300021169 | Ga0206687_1069781 | 6208 | 20 | 9 | Euryarchaeota archaeon | Marine |
| 3300009677 | Ga0115104_10562447 | 5257 | 12 | 8 | Unclassified | Marine |
| 3300004799 | Ga0058863_10012509 | 2901 | 11 | 7 | Bacteria | Plants |
| 3300008962 | Ga0104242_1001916 | 4124 | 16 | 7 | Alphaproteobacteria | Freshwater |
| 3300007758 | Ga0105668_1024074 | 7407 | 17 | 6 | Viruses | Marine |
| 3300008953 | Ga0104241_1001038 | 2197 | 10 | 6 | Viruses | Freshwater |
| 3300008962 | Ga0104242_1018371 | 1214 | 7 | 6 | Viruses | Freshwater |
| 3300009606 | Ga0115102_10602158 | 4432 | 13 | 6 | Euryarchaeota archaeon] | Marine |
| 3300012702 | Ga0157596_1119075 | 7291 | 15 | 6 | Unclassified | Freshwater |
| 3300018775 | Ga0188848_1000171 | 8698 | 27 | 6 | Unclassified | Marine |
| 3300020069 | Ga0197907_10727879 | 2599 | 9 | 6 | Unclassified | Soil |
| 3300024345 | Ga0255062_10000013 | 25683 | 53 | 6 | Unclassified | Mammals |
| 3300000827 | JGI12396J12026_1139132 | 1861 | 7 | 5 | Unclassified | Soil |
| 3300003153 | Ga0052192_1004136 | 2887 | 9 | 5 | Unclassified | Marine |
| 3300004763 | Ga0007746_1044746 | 4032 | 9 | 5 | Viruses | Freshwater |
| 3300004799 | Ga0058863_10092598 | 3106 | 12 | 5 | Unclassified | Human |
| 3300004799 | Ga0058863_11965738 | 1729 | 6 | 5 | Unclassified | Human |
| 3300005248 | Ga0068636_1045156 | 2280 | 6 | 5 | Unclassified | Aquatic (Thermal Springs) |
| 3300005416 | Ga0068880_1422077 | 2597 | 7 | 5 | Unclassified | Freshwater |

**Table S12.** Top-60 largest protein families

| **Family ID** | **Type** | **Size (aa)** | **Number of genes** | **Number of Scaffolds** | **Number of Datasets** | **Taxonomy** | **Habitat** | **3D Model** |
| --- | --- | --- | --- | --- | --- | --- | --- | --- |
| **F000001** | MetaG | 44 | 16856 | 16821 | 31 | Eukaryotic | Termite Gut (99%) | Yes |
| **F000002** | MetaG | 62 | 15118 | 15087 | 29 | Eukaryotic | Gutless Worms (98%) | Yes |
| **F000003** | MetaG | 44 | 14941 | 11591 | 58 | Eukaryotic | Termite Gut (98%) | Yes |
| **F000004** | MetaG | 41 | 10528 | 10511 | 21 | Eukaryotic | Termite Gut (100%) | Yes |
| **F000005** | MetaG | 73 | 10444 | 10422 | 20 | Eukaryotic | Gutless Worms (99%) | Yes |
| **F000006** | MetaG | 43 | 8944 | 8936 | 57 | Eukaryotic | Termite Gut (98%) | Yes |
| **F000007** | MetaG/  MetaT | 147 | 8691 | 8688 | 691 | Eukaryotic/Protozoa | Marine (39%) | No |
| **F000008** | MetaG | 44 | 8622 | 8611 | 32 | Eukaryotic | Termite Gut (99%) | Yes |
| **F000009** | MetaG | 104 | 8556 | 8544 | 59 | Eukaryotic | Termite Gut (98%) | Yes |
| **F000010** | MetaG | 72 | 7424 | 6352 | 12 | Eukaryotic & Bacterial | Root Nodules (65.28%) | Yes |
| **F000011** | MetaG | 41 | 7286 | 7282 | 17 | Eukaryotic | Termite Gut (99%) | Yes |
| **F000012** | MetaG | 82 | 7108 | 6917 | 26 | Eukaryotic | Gutless Worms (71%) | Yes |
| **F000013** | MetaG | 42 | 7082 | 7073 | 46 | Eukaryotic | Gutless Worms (82%) | Yes |
| **F000014** | MetaG | 42 | 6831 | 6830 | 34 | Eukaryotic | Gutless Worms (92%) | Yes |
| **F000015** | MetaG | 46 | 6687 | 6686 | 50 | Eukaryotic | Termite Gut (99%) | Yes |
| **F000016** | MetaG | 43 | 6618 | 6612 | 42 | Eukaryotic | Termite Gut (99%) | Yes |
| **F000017** | MetaG | 52 | 6504 | 6498 | 33 | Eukaryotic | Gutless Worms (60%) | Yes |
| **F000018** | MetaG | 58 | 6467 | 6460 | 41 | Eukaryotic | Termite Gut (97%) | Yes |
| **F000019** | MetaG | 54 | 6343 | 6287 | 32 | Eukaryotic | Termite Gut (99%) | Yes |
| **F000020** | MetaG/  MetaT | 187 | 6197 | 6197 | 662 | Eukaryotic/Protozoa | Marine (40%) | Yes |
| **F000021** | MetaG/  MetaT | 228 | 6082 | 6082 | 704 | Eukaryotic/Crustacea | Marine (86%) | Yes |
| **F000022** | MetaG | 42 | 6021 | 6016 | 47 | Eukaryotic | Termite Gut (98%) | Yes |
| **F000023** | MetaG/  MetaT | 236 | 5736 | 5732 | 755 | Eukaryotic/Protozoa | Marine (49%) | No |
| **F000024** | MetaG | 74 | 5722 | 5716 | 27 | Eukaryotic | Gutless Worms (74%) | Yes |
| **F000025** | MetaG/  MetaT | 184 | 5578 | 5573 | 362 | Eukaryotic/Protozoa | Soil (80%) | Yes |
| **F000026** | MetaG | 50 | 5546 | 5412 | 17 | Eukaryotic | Termite Gut (100%) | Yes |
| **F000027** | MetaG | 52 | 5489 | 5455 | 18 | Eukaryotic | Gutless Worms (99%) | Yes |
| **F000028** | MetaG | 46 | 5420 | 5417 | 24 | Eukaryotic | Termite Gut (99%) | Yes |
| **F000029** | MetaG | 100 | 5053 | 4423 | 12 | Eukaryotic/Plant | Root nodules (66%) | Yes |
| **F000030** | MetaG | 43 | 5002 | 4995 | 50 | Eukaryotic | Termite Gut (99%) | Yes |
| **F000031** | MetaG | 51 | 4897 | 4882 | 52 | Eukaryotic | Termite Gut (99%) | Yes |
| **F000032** | MetaG | 56 | 4750 | 4744 | 55 | Eukaryotic | Termite Gut (98%) | Yes |
| **F000033** | MetaG | 45 | 4610 | 4610 | 41 | Eukaryotic | Termite Gut (98%) | Yes |
| **F000034** | MetaG | 50 | 4504 | 4500 | 15 | Eukaryotic | Termite Gut (99%) | Yes |
| **F000035** | MetaG | 65 | 4444 | 4444 | 46 | Eukaryotic | Gutless Worms (75%) | Yes |
| **F000036** | MetaG/  MetaT | 134 | 4352 | 4352 | 1128 | Bacteria/Acidobacteria | Soil (12%) | Yes |
| **F000037** | MetaG/  MetaT | 84 | 4230 | 4229 | 512 | Bacteria | Soil (24%) | Yes |
| **F000038** | MetaG | 72 | 4061 | 4053 | 53 | Eukaryotic | Termite Gut (99%) | Yes |
| **F000039** | MetaG/  MetaT | 571 | 4032 | 4019 | 554 | Eukaryotic Crustacea | Marine (86%) | Yes |
| **F000040** | MetaG/  MetaT | 214 | 3998 | 3998 | 511 | Eukaryotic/Protozoa | Marine (58%) | Yes |
| **F000041** | MetaG | 39 | 3975 | 3972 | 14 | Eukaryotic | Termite Gut (100%) | Yes |
| **F000042** | MetaG/  MetaT | 69 | 3762 | 3761 | 987 | Bacteria/Acidobacteria | Soil (9%) | Yes |
| **F000043** | MetaG/  MetaT | 82 | 3655 | 3655 | 844 | Bacteria/Acidobacteria | Soil (20%) | Yes |
| **F000044** | MetaG/  MetaT | 287 | 3572 | 3572 | 505 | Eukaryotic/Crustacea | Marine (85%) | Yes |
| **F000045** | MetaG | 41 | 3463 | 3445 | 47 | Eukaryotic | Termite Gut (99%) | Yes |
| **F000046** | MetaG | 40 | 3442 | 3441 | 48 | Eukaryotic | Termite Gut (99%) | Yes |
| **F000047** | MetaG | 60 | 3366 | 3364 | 52 | Eukaryotic | Termite Gut (96%) | Yes |
| **F000048** | MetaG/  MetaT | 118 | 3365 | 3365 | 689 | Eukaryotic/Crustacea | Marine (81%) | Yes |
| **F000049** | MetaG/  MetaT | 209 | 3277 | 3277 | 657 | Eukaryotic/Crustacea | Marine (76%) | Yes |
| **F000050** | MetaG | 38 | 3273 | 3269 | 52 | Eukaryotic | Termite Gut (89%) | Yes |
| **F000051** | MetaG/  MetaT | 220 | 3266 | 3266 | 638 | Eukaryotic/Crustacea | Marine (82%) | Yes |
| **F000052** | MetaG/  MetaT | 207 | 3223 | 3223 | 556 | Eukaryotic/Crustacea | Marine (88%) | Yes |
| **F000053** | MetaG | 41 | 3216 | 3213 | 16 | Eukaryotic | Termite Gut (100%) | Yes |
| **F000054** | MetaG | 47 | 3147 | 3144 | 12 | Eukaryotic | Termite Gut (100%) | Yes |
| **F000055** | MetaG/  MetaT | 145 | 3096 | 3095 | 901 | Unclassified | Marine (33%) | Yes |
| **F000056** | MetaG | 49 | 3057 | 2971 | 9 | Unclassified | Root Nodules (60.17%) | Yes |
| **F000057** | MetaG/  MetaT | 89 | 3033 | 3031 | 624 | Viral/Phage | Marine (58%) | Yes |
| **F000058** | MetaG | 56 | 3020 | 2992 | 17 | Eukaryotic | Termite Gut (96%) | Yes |
| **F000059** | MetaG/  MetaT | 201 | 3008 | 3008 | 495 | Eukaryotic/Protozoa | Marine (57%) | Yes |
| **F000060** | MetaG/  MetaT | 194 | 2944 | 2944 | 606 | Viral/Phage | Marine (57%) | Yes |

**Table S13.** JGI Proposal Award DOIs

10.46936/10.25585/60000801

10.46936/10.25585/60007486

10.46936/10.25585/60000776

10.46936/10.25585/60001428

10.46936/10.25585/60000718

10.46936/10.25585/60000619

10.46936/jejc.proj.2014.48484/60005502

10.46936/10.25585/60007560

10.46936/10.25585/60000867

10.46936/10.25585/60000955

10.46936/10.25585/60007461

10.46936/10.25585/60000771

10.46936/10.25585/60000968

10.46936/10.25585/60000788

10.46936/10.25585/60007637

10.46936/10.25585/60001134

10.46936/10.25585/60001009

10.46936/10.25585/60000868

10.46936/10.25585/60000893

10.46936/jejc.proj.2015.49001/60005769

10.46936/10.25585/60000797

10.46936/10.25585/60001171

10.46936/10.25585/60007294

10.46936/10.25585/60001198

10.46936/10.25585/60001122

10.46936/10.25585/60007706

10.46936/jejc.proj.2015.49017/60005777

10.46936/10.25585/60000972

10.46936/10.25585/60001034

10.46936/10.25585/60001181

10.46936/jejc.proj.2014.48475/60005498

10.46936/10.25585/60007615

10.46936/10.25585/60001247

10.46936/10.25585/60001018

10.46936/10.25585/60000598

10.46936/10.25585/60007271

10.46936/10.25585/60000643

10.46936/fics.proj.2016.49483/60006003

10.46936/10.25585/60000701

10.46936/10.25585/60001099

10.46936/10.25585/60007261

10.46936/10.25585/60007995

10.46936/10.25585/60001011

10.46936/10.25585/60000799

10.46936/10.25585/60000951

10.46936/10.25585/60000711

10.46936/jejc.proj.2014.48462/60005494

10.46936/10.25585/60001414

10.46936/10.25585/60000538

10.46936/10.25585/60000876

10.46936/10.25585/60007558

10.46936/10.25585/60000575

10.46936/10.25585/60000875

10.46936/10.25585/60008073

10.46936/10.25585/60000734

10.46936/10.25585/60007713

10.46936/10.25585/60000804

10.46936/10.25585/60000745

10.46936/10.25585/60007217

10.46936/10.25585/60000756

10.46936/10.25585/60001061

10.46936/10.25585/60001132

10.46936/10.25585/60000605

10.46936/10.25585/60000895

10.46936/10.25585/60000947

10.46936/10.25585/60000753

10.46936/10.25585/60000922

10.46936/10.25585/60008081

10.46936/10.25585/60000544

10.46936/10.25585/60000811

10.46936/10.25585/60000574

10.46936/10.25585/60007723

10.46936/10.25585/60007340

10.46936/fics.proj.2017.49950/60006215

10.46936/10.25585/60007348

10.46936/10.25585/60000603

10.46936/10.25585/60007710

10.46936/10.25585/60000668

10.46936/10.25585/60000846

10.46936/10.25585/60000910

10.46936/10.25585/60001037

10.46936/10.25585/60000878

10.46936/10.25585/60000818

10.46936/10.25585/60007372

10.46936/10.25585/60000713

10.46936/10.25585/60001074

10.46936/10.25585/60000791

10.46936/10.25585/60001394

10.46936/10.25585/60001107

10.46936/10.25585/60008084

10.46936/10.25585/60000624

10.46936/10.25585/60001042

10.46936/10.25585/60000930

10.46936/10.25585/60001052

10.46936/10.25585/60000933

10.46936/10.25585/60007699

10.46936/10.25585/60000733

10.46936/fics.proj.2016.49505/60006013

10.46936/fics.proj.2016.49500/60006011

10.46936/10.25585/60000772

10.46936/10.25585/60000954

10.46936/jejc.proj.2013.48086/60005298

10.46936/fics.proj.2016.49518/60006017

10.46936/10.25585/60000573

10.46936/10.25585/60000783

10.46936/10.25585/60000715

10.46936/10.25585/60001020

10.46936/fics.proj.2016.49521/60006018

10.46936/10.25585/60007428

10.46936/10.25585/60000686

10.46936/10.25585/60000890

10.46936/10.25585/60007659

10.46936/10.25585/60000542

10.46936/10.25585/60000944

10.46936/10.25585/60007622

10.46936/10.25585/60000814

10.46936/10.25585/60000762

10.46936/10.25585/60001090

10.46936/10.25585/60007259

10.46936/10.25585/60000682

10.46936/10.25585/60007354

10.46936/10.25585/60008082

10.46936/10.25585/60000667

10.46936/10.25585/60001040

10.46936/10.25585/60007263

10.46936/10.25585/60007411

10.46936/10.25585/60000870

10.46936/10.25585/60000790

10.46936/10.25585/60007422

10.46936/10.25585/60000533

10.46936/10.25585/60000926

10.46936/fics.proj.2017.49972/60000022

10.46936/10.25585/60000920

10.46936/10.25585/60000770

10.46936/10.25585/60000936

10.46936/10.25585/60007702

10.46936/jejc.proj.2014.48473/60005497

10.46936/10.25585/60007594

10.46936/10.25585/60000806

10.46936/10.25585/60007508

10.46936/10.25585/60000747

10.46936/10.25585/60000923

10.46936/10.25585/60007430

10.46936/10.25585/60007557

10.46936/10.25585/60001100

10.46936/10.25585/60000938

10.46936/10.25585/60000758

10.46936/10.25585/60001148

10.46936/10.25585/60000820

10.46936/jejc.proj.2014.48467/60005495

10.46936/10.25585/60000684

10.46936/10.25585/60007693

10.46936/10.25585/60000874

10.46936/10.25585/60000545

10.46936/10.25585/60000730

10.46936/10.25585/60001409

10.46936/10.25585/60007606

10.46936/10.25585/60000582

10.46936/10.25585/60000751

10.46936/10.25585/60000702

10.46936/10.25585/60007685

10.46936/10.25585/60000714

10.46936/10.25585/60007585

10.46936/fics.proj.2016.49499/60006010

10.46936/10.25585/60008057

10.46936/10.25585/60000604

10.46936/10.25585/60000831

10.46936/10.25585/60007535

10.46936/fics.proj.2017.49965/60006226

10.46936/10.25585/60007636

10.46936/10.25585/60000754

10.46936/10.25585/60008379

10.46936/10.25585/60007579

10.46936/10.25585/60000621

10.46936/10.25585/60000741

10.46936/10.25585/60007726

10.46936/10.25585/60007373

10.46936/10.25585/60000813

10.46936/10.25585/60000618

10.46936/10.25585/60001027

10.46936/10.25585/60000623

10.46936/10.25585/60000567

10.46936/10.25585/60000720

10.46936/10.25585/60000697

10.46936/10.25585/60000543

10.46936/10.25585/60007339

10.46936/10.25585/60001056

10.46936/10.25585/60007402

10.46936/10.25585/60000694

10.46936/10.25585/60000787

10.46936/10.25585/60000626

10.46936/10.25585/60001141

10.46936/10.25585/60000873

10.46936/10.25585/60000564

10.46936/10.25585/60007640

10.46936/10.25585/60007267

10.46936/10.25585/60001098

10.46936/10.25585/60000625

10.46936/10.25585/60000627

10.46936/10.25585/60000967

10.46936/jejc.proj.2014.48483/60005501

10.46936/10.25585/60000795

10.46936/10.25585/60000739

10.46936/10.25585/60000779

10.46936/10.25585/60000872

10.46936/10.25585/60000857

10.46936/10.25585/60000909

10.46936/10.25585/60000748

10.46936/10.25585/60000614

10.46936/10.25585/60007683

10.46936/10.25585/60007412

10.46936/10.25585/60000611

10.46936/10.25585/60007981

10.46936/10.25585/60000796

10.46936/10.25585/60000809

10.46936/10.25585/60001413

10.46936/10.25585/60007520

10.46936/10.25585/60007517

10.46936/10.25585/60000553

10.46936/10.25585/60000792

10.46936/10.25585/60000736

10.46936/10.25585/60001004

10.46936/10.25585/60007487

10.46936/10.25585/60001066

10.46936/10.25585/60007583

10.46936/jejc.proj.2013.48085/60005297

10.46936/10.25585/60000666

10.46936/10.25585/60000726

10.46936/10.25585/60000907

10.46936/10.25585/60000871

10.46936/10.25585/60000616

10.46936/10.25585/60007369

10.46936/10.25585/60000717

10.46936/10.25585/60000700

10.46936/10.25585/60008322

10.46936/10.25585/60001035

10.46936/10.25585/60000869

10.46936/10.25585/60007481

10.46936/10.25585/60000755

10.46936/10.25585/60000622

10.46936/10.25585/60000919

10.46936/fics.proj.2017.49991/60006232

10.46936/10.25585/60001053

10.46936/10.25585/60000917

10.46936/10.25585/60000833

10.46936/10.25585/60000744

10.46936/10.25585/60007720

10.46936/10.25585/60007523

10.46936/10.25585/60007571

10.46936/10.25585/60000866

10.46936/10.25585/60000849

10.46936/10.25585/60007445

10.46936/10.25585/60000880

10.46936/10.25585/60000800

10.46936/10.25585/60000798

10.46936/10.25585/60001047

10.46936/10.25585/60000911

10.46936/10.25585/60000572

10.46936/10.25585/60001131

10.46936/10.25585/60007414

10.46936/10.25585/60007695

10.46936/10.25585/60000921

10.46936/10.25585/60000587

10.46936/10.25585/60007255

10.46936/10.25585/60007533

10.46936/10.25585/60001140

10.46936/10.25585/60008240

10.46936/10.25585/60000901

10.46936/10.25585/60000685

10.46936/10.25585/60000847

10.46936/jejc.proj.2013.48099/60005301

10.46936/10.25585/60007639

10.46936/10.25585/60007337

10.46936/10.25585/60007591

10.46936/10.25585/60000737

10.46936/10.25585/60001094

10.46936/10.25585/60007671

10.46936/fics.proj.2017.49986/60006230

10.46936/10.25585/60001045

10.46936/10.25585/60007694

10.46936/10.25585/60000648

10.46936/10.25585/60000719

10.46936/10.25585/60000578

10.46936/10.25585/60000986

10.46936/10.25585/60000888

10.46936/10.25585/60007503

10.46936/10.25585/60000929

## Supplementary References

[1. Hauser, M., Mayer, C. E. & Söding, J. kClust: fast and sensitive clustering of large protein sequence databases. *BMC Bioinformatics* **14**, 248 (2013).](https://www.zotero.org/google-docs/?UuBr4b)

[2. Edgar, R. C. Search and clustering orders of magnitude faster than BLAST. *Bioinformatics* **26**, 2460–2461 (2010).](https://www.zotero.org/google-docs/?UuBr4b)

[3. Li, W. & Godzik, A. Cd-hit: a fast program for clustering and comparing large sets of protein or nucleotide sequences. *Bioinformatics* **22**, 1658–1659 (2006).](https://www.zotero.org/google-docs/?UuBr4b)

[4. Steinegger, M. & Söding, J. MMseqs2 enables sensitive protein sequence searching for the analysis of massive data sets. *Nat Biotechnol* **35**, 1026–1028 (2017).](https://www.zotero.org/google-docs/?UuBr4b)

[5. Steinegger, M. & Söding, J. Clustering huge protein sequence sets in linear time. *Nat Commun* **9**, 2542 (2018).](https://www.zotero.org/google-docs/?UuBr4b)

[6. Jiang, P. & Singh, M. SPICi: a fast clustering algorithm for large biological networks. *Bioinformatics* **26**, 1105–1111 (2010).](https://www.zotero.org/google-docs/?UuBr4b)

[7. Blondel, V. D., Guillaume, J.-L., Lambiotte, R. & Lefebvre, E. Fast unfolding of communities in large networks. *J. Stat. Mech.* **2008**, P10008 (2008).](https://www.zotero.org/google-docs/?UuBr4b)

[8. Bader, G. D. & Hogue, C. W. An automated method for finding molecular complexes in large protein interaction networks. *BMC Bioinformatics* **4**, 2 (2003).](https://www.zotero.org/google-docs/?UuBr4b)

[9. Frey, B. J. & Dueck, D. Clustering by Passing Messages Between Data Points. *Science* **315**, 972–976 (2007).](https://www.zotero.org/google-docs/?UuBr4b)

[10. Enright, A. J., Van Dongen, S. & Ouzounis, C. A. An efficient algorithm for large-scale detection of protein families. *Nucleic Acids Research* **30**, 1575–1584 (2002).](https://www.zotero.org/google-docs/?UuBr4b)

[11. Azad, A., Pavlopoulos, G. A., Ouzounis, C. A., Kyrpides, N. C. & Buluç, A. HipMCL: a high-performance parallel implementation of the Markov clustering algorithm for large-scale networks. *Nucleic Acids Research* **46**, e33 (2018).](https://www.zotero.org/google-docs/?UuBr4b)

[12. Suzek, B. E., Wang, Y., Huang, H., McGarvey, P. B. & Wu, C. H. UniRef clusters: a comprehensive and scalable alternative for improving sequence similarity searches. *Bioinformatics* **31**, 926–932 (2015).](https://www.zotero.org/google-docs/?UuBr4b)

[13. Rost, B. Twilight zone of protein sequence alignments. *Protein Eng* **12**, 85–94 (1999).](https://www.zotero.org/google-docs/?UuBr4b)

[14. Mitchell, A. L. *et al.* MGnify: the microbiome analysis resource in 2020. *Nucleic Acids Res* **48**, D570–D578 (2020).](https://www.zotero.org/google-docs/?UuBr4b)

[15. Eddy, S. R. Accelerated Profile HMM Searches. *PLOS Computational Biology* **7**, e1002195 (2011).](https://www.zotero.org/google-docs/?UuBr4b)

[16. Bryant, P., Pozzati, G. & Elofsson, A. Improved prediction of protein-protein interactions using AlphaFold2. *Nat Commun* **13**, 1265 (2022).](https://www.zotero.org/google-docs/?UuBr4b)

[17. Clum, A. *et al.* DOE JGI Metagenome Workflow. *mSystems* **6**, e00804-20 (2021).](https://www.zotero.org/google-docs/?UuBr4b)

[18. Chan, P. P., Lin, B. Y., Mak, A. J. & Lowe, T. M. tRNAscan-SE 2.0: improved detection and functional classification of transfer RNA genes. *Nucleic Acids Research* **49**, 9077–9096 (2021).](https://www.zotero.org/google-docs/?UuBr4b)

[19. Nawrocki, E. P. & Eddy, S. R. Infernal 1.1: 100-fold faster RNA homology searches. *Bioinformatics* **29**, 2933–2935 (2013).](https://www.zotero.org/google-docs/?UuBr4b)

[20. Kalvari, I. *et al.* Rfam 14: expanded coverage of metagenomic, viral and microRNA families. *Nucleic Acids Research* **49**, D192–D200 (2021).](https://www.zotero.org/google-docs/?UuBr4b)

[21. Hyatt, D. *et al.* Prodigal: prokaryotic gene recognition and translation initiation site identification. *BMC Bioinformatics* **11**, 119 (2010).](https://www.zotero.org/google-docs/?UuBr4b)

[22. Lomsadze, A., Gemayel, K., Tang, S. & Borodovsky, M. Modeling leaderless transcription and atypical genes results in more accurate gene prediction in prokaryotes. *Genome Res* **28**, 1079–1089 (2018).](https://www.zotero.org/google-docs/?UuBr4b)

[23. Roux, S. *et al.* IMG/VR v3: an integrated ecological and evolutionary framework for interrogating genomes of uncultivated viruses. *Nucleic Acids Research* **49**, D764–D775 (2021).](https://www.zotero.org/google-docs/?UuBr4b)

[24. Ren, J. *et al.* Identifying viruses from metagenomic data using deep learning. *Quant Biol* **8**, 64–77 (2020).](https://www.zotero.org/google-docs/?UuBr4b)

[25. Pronk, L. J. U. & Medema, M. H. Whokaryote: distinguishing eukaryotic and prokaryotic contigs in metagenomes based on gene structure. *Microbial Genomics* **8**, (2022).](https://www.zotero.org/google-docs/?UuBr4b)

[26. West, P. T., Probst, A. J., Grigoriev, I. V., Thomas, B. C. & Banfield, J. F. Genome-reconstruction for eukaryotes from complex natural microbial communities. *Genome Res* **28**, 569–580 (2018).](https://www.zotero.org/google-docs/?UuBr4b)

[27. Delmont, T. O. *et al.* Functional repertoire convergence of distantly related eukaryotic plankton lineages abundant in the sunlit ocean. *Cell Genomics* **2**, 100123 (2022).](https://www.zotero.org/google-docs/?UuBr4b)

[28. Mirdita, M., Steinegger, M., Breitwieser, F., Söding, J. & Levy Karin, E. Fast and sensitive taxonomic assignment to metagenomic contigs. *Bioinformatics* **37**, 3029–3031 (2021).](https://www.zotero.org/google-docs/?UuBr4b)

[29. Chandramohan, R., Yang, C., Cai, Y. & Wang, M. D. Metagenomics for Monitoring Environmental Biodiversity: Challenges, Progress, and Opportunities. in *Health Informatics Data Analysis: Methods and Examples* (eds. Xu, D., Wang, M. D., Zhou, F. & Cai, Y.) 73–87 (Springer International Publishing, 2017). doi:10.1007/978-3-319-44981-4_5.](https://www.zotero.org/google-docs/?UuBr4b)

[30. Baltoumas, F. A. *et al.* Exploring microbial functional biodiversity at the protein family level-From metagenomic sequence reads to annotated protein clusters. *Front Bioinform* **3**, 1157956 (2023).](https://www.zotero.org/google-docs/?UuBr4b)
